# Rapid repair of human disease-specific single-nucleotide variants by One-SHOT genome editing

**DOI:** 10.1038/s41598-020-70401-7

**Published:** 2020-08-18

**Authors:** Yuji Yokouchi, Shinichi Suzuki, Noriko Ohtsuki, Kei Yamamoto, Satomi Noguchi, Yumi Soejima, Mizuki Goto, Ken Ishioka, Izumi Nakamura, Satoru Suzuki, Seiichi Takenoshita, Takumi Era

**Affiliations:** 1 Pluripotent Stem Cell Research Unit in Department of Thyroid and Endocrinology, School of Medicine, Fukushima Medical University, 1 Hikariga-oka, Fukushima, 960-1295 Japan; 2 Department of Thyroid and Endocrinology, School of Medicine, Fukushima Medical University, Fukushima, Japan; 3 Department of Cell Modulation, Institute of Molecular Embryology and Genetics (IMEG), Kumamoto University, Kumamoto, Japan; 4 Department of Dermatology, Faculty of Medicine, Oita University, Yufu, Japan; 5 Department of Microbiology, School of Medicine, Fukushima Medical University, Fukushima, Japan; 6 Office of Thyroid Ultrasound Examination Promotion, Radiation Medical Science Centre for the Fukushima Health Management Survey, Fukushima Medical University, Fukushima, Japan; 7 Fukushima Medical University, Fukushima, Japan

**Keywords:** Stem-cell research, Targeted gene repair, Mutagenesis, Personalized medicine, Medical genomics, Synthetic biology, Regenerative medicine, Stem-cell biotechnology, CRISPR-Cas9 genome editing, Genetic engineering, Genetic techniques, Cell culture, Cancer, Endocrine system and metabolic diseases, Skin diseases

## Abstract

Many human diseases ranging from cancer to hereditary disorders are caused by single-nucleotide mutations in critical genes. Repairing these mutations would significantly improve the quality of life for patients with hereditary diseases. However, current procedures for repairing deleterious single-nucleotide mutations are not straightforward, requiring multiple steps and taking several months to complete. In the current study, we aimed to repair pathogenic allele-specific single-nucleotide mutations using a single round of genome editing. Using high-fidelity, site-specific nuclease *As*Cas12a/Cpf1, we attempted to repair pathogenic single-nucleotide variants (SNVs) in disease-specific induced pluripotent stem cells. As a result, we achieved repair of the Met918Thr SNV in human oncogene *RET* with the inclusion of a single-nucleotide marker, followed by absolute markerless, scarless repair of the *RET* SNV with no detected off-target effects. The markerless method was then confirmed in human type VII collagen-encoding gene *COL7A1*. Thus, using this One-SHOT method, we successfully reduced the number of genetic manipulations required for genome repair from two consecutive events to one, resulting in allele-specific repair that can be completed within 3 weeks, with or without a single-nucleotide marker. Our findings suggest that One-SHOT can be used to repair other types of mutations, with potential beyond human medicine.

## Introduction

The human genome contains extensive variation, including an estimated 5 × 10^6^ single-nucleotide variants (SNVs) that determine how we look and function, as well as our specific disease tendencies^1–3^. Some SNVs are pathogenic and either directly or indirectly cause hereditary disorders^4–6^ such as multiple endocrine neoplasia type 2B (MEN2B)^7^ and dystrophic epidermolysis bullosa (DEB)^8^. MEN2B is an autosomal dominant syndrome characterised by thyroid, adrenal gland and neuronal tumours and skeletal abnormalities. The majority of MEN2B cases (95%) result from a single amino-acid substitution (Met918Thr) in the *RET* proto-oncogene, which is caused by a pathogenic SNV (*RET*: c.2753T>C) at the second base of the codon^7^. DEB is an inherited disease characterised by severe, recurrent skin ulcers and blistering. It is caused by genetic mutations in the human type VII collagen-encoding gene *COL7A1*, the product of which is an anchoring fibril connecting the epidermis to the dermis^8^. To model diseases such as these in vitro, disease-specific induced pluripotent stem cells (iPSCs) carrying pathogenic SNVs or other genetic mutations can be obtained from patients^9–12^. Repairing these iPSCs to generate isogenic revertant cells is a promising strategy for genome repair, and opens up new avenues for drug discovery^13,14^. However, the repair process remains problematic, and a precise and convenient genome editing procedure has not yet been developed.

Artificial genome repair and/or modification generally starts from a target-specific double-strand break generated by site-specific nucleases^15,16^. Double-strand breaks are then repaired by a cell’s own genome repair machineries^15,16^. However, most of the break sites are incorrectly repaired by non-homologous end joining, and can result in gene knockout through the generation of non-specific insertions or deletions (indels)^17,18^. If a repair template carrying a repair base is co-administrated, however, the cleavage sites can be accurately repaired via homology-directed repair (HDR)^15,16^. Site-specific nucleases, such as transcription activator-like effector nucleases or the *Streptococcus pyogenes* (*Sp*) Cas9 nuclease, are typically used as genome editing tools for human iPSCs^19–21^.

*Sp*Cas9 is a type II-A endonuclease in the class 2 clustered regularly interspaced short palindromic repeats (CRISPR)-Cas system that has been repurposed as a programmable site-specific nuclease for genome engineering^22–25^. Indeed, *Sp*Cas9 has become a popular genome editing tool for genetically modifying human pluripotent stem cells^19^. Despite its efficient cleavage activity, wild-type *Sp*Cas9 has a low DNA repair rate using HDR following plasmid-based administration. It re-cuts the repaired site because the guide RNA has a 1–2-base mismatch tolerance during sequence recognition, leading to incorrect repair by non-homologous end joining^17,18^. To prevent re-cutting, a blocking mutation must be introduced into the seed sequence of the guide RNA or into the protospacer-adjacent motif (PAM)^26–31^.

However, for “scarless” genome editing repairs with wild-type *Sp*Cas9^27,49^, each method has its obvious strengths and weaknesses. For example, CORRECT, which includes excellent tricks to prevent re-cutting of the edited target by the editing tool, can be performed even if the target recognition ability of the genome editing tool is insufficient; however, the need for two consecutive editing steps^27^. In comparison, MhAX has high genome editing efficiency but cannot achieve completely scarless editing because single-base markers are required. Further, as with the CORRECT method, MhAX editing requires two consecutive edits^49^, increasing cost and time requirements.

Another recently-identified bacterial programmable site-specific nuclease, CRISPR-Cas12a/Cpf1, is a type V-A endonuclease belonging to the class 2 CRISPR-Cas system^32^. Among identified Cas12a enzymes, those from *Acidaminococcus* sp. BV3L6 (*As*) and *Lachnospiraceae* bacterium ND2006 show strong cleavage activity in mammalian cells^32^. These Cas12a endonucleases have unique features that complement Cas9, and expand the genome editing range. First, Cas12a recognises a T-rich PAM upstream of the protospacer, whereas Cas9 recognises a G-rich PAM downstream of the protospacer^32^. Second, two PAM-interacting variants have been generated that expand the Cas12a target range^33,34^. Third, Cas12a-mediated cleavage generates a staggered cut on the PAM-distal region of the target sequence, as opposed to the PAM-proximal blunt ends generated by Cas9^32^. Finally, and perhaps most importantly, the guide or CRISPR RNA (crRNA) exhibits high-fidelity target recognition, meaning that Cas12a can precisely distinguish the target sequence at a single-base resolution^35,36^. Consequently, the resulting off-target effects are kept to a background level. These features suggest that Cas12a might be suitable for precise, disease-specific iPSC repair because its re-cut activity is low.

Cas12a has already been used to knock out pathogenic genes in cancer cells^37^, generate insertions or two-nucleotide substitutions in iPSCs^38^ and to induce exon-skipping in disease-specific iPSCs^39^. Thus, we investigated whether Cas12a could be used to carry out allele-specific single-nucleotide repair of iPSCs carrying the pathogenic SNVs found in MEN2B and DEB patients in a single round of genome editing. To accomplish this, we used an *As*Cas12a PAM variant and single-nucleotide mismatch detection polymerase chain reaction (SNMD-PCR) analysis in disease-specific iPSCs to develop a precise, convenient genome-editing procedure we have called One-SHOT (One allele-specific, single HDR and single-stranded oligodeoxynucleotide (ssODN), transient drug selection with SNMD-PCR screening). One-SHOT provides scarless single-nucleotide substitution of a pathogenic SNV in disease-specific iPSCs within 3 weeks. The final modification rate is within a practical range for hand-picking cloning. Our findings suggest that this simple, low cost procedure could be used for genome editing in a single step, drastically reducing the time currently needed for scarless SNV repair.

## Results

### Principles of One-SHOT repair of single-nucleotide mutations

**Figure 1:**
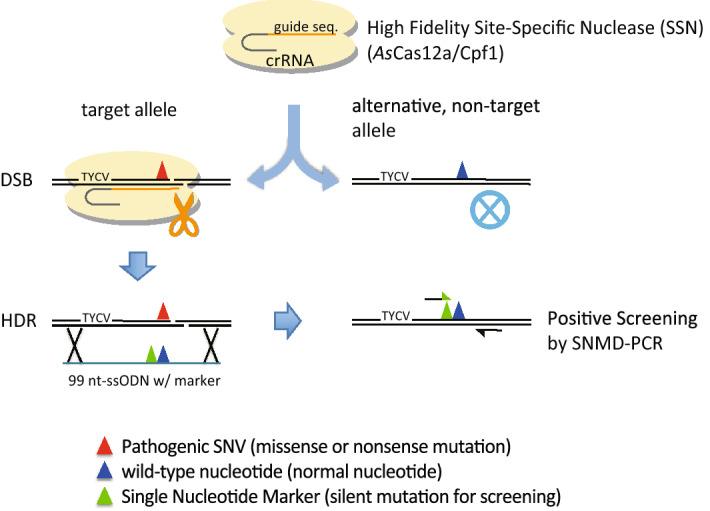
One-SHOT principles. *As*Cas12a (pale yellow) and crRNA (orange and grey lines) selectively bind to a target sequence containing a pathogenic SNV (red triangle) on the target allele. Binding leads to a double-strand break in the target sequence on the target allele (left) but not in the corresponding wild-type sequence containing the wild-type nucleotide (blue triangle) on the alternative, non-target (wild-type) allele (right). When the ssODN repair template (blue-green line) with the wild-type nucleotide (blue triangle) and a single-nucleotide marker (a silent mutation for SNMD-PCR screening, green triangle) is co-transfected with *As*Cas12a into the cells, the target site on the pathogenic allele is repaired using the template by the endogenous HDR machinery. In this case, the intended gene-edited clones are easily identified by positive screening for the single-nucleotide marker because the repaired, ex-pathogenic allele now carries the single-nucleotide marker.

*As*Cas12a is a high-fidelity RNA-guided site-specific nuclease that binds to the target genomic DNA site via a 20-nt guide sequence in the crRNA, allowing it to discriminate the target sequence at the single nucleotide level (Fig. 1). Following the addition of a crRNA designed for a specific target sequence containing a single-nucleotide mutation, *As*Cas12a selectively binds to the target sequence on the mutant allele and induces a double-strand break, leaving the wild-type sequence on the alternative allele unaffected (Fig. 1). In the presence of a ssODN wild-type sequence template, the mutant nucleotide in the target sequence can be “repaired” to the wild-type sequence via the cellular HDR machinery. To mark the repaired allele, we labelled the ssODN with a single-nucleotide marker in the vicinity of the mutant nucleotide. This label allowed us to easily identify the gene-repaired clones by allele-specific amplification^40–42^/SNMD-PCR detection of the single-nucleotide marker (Fig. 1). A complete outline of the One-SHOT workflow for SNV repair is provided in the Supplementary Information and in Supplementary Fig. S1.

### Allele-specific single-nucleotide substitution in MEN2B-specific iPSCs

Before carrying out allele-specific single-nucleotide repair of the pathogenic *RET* mutation, we assessed whether the One-SHOT approach could be used to accomplish allele-specific single-nucleotide substitution of the wild-type allele.

**Figure 2:**
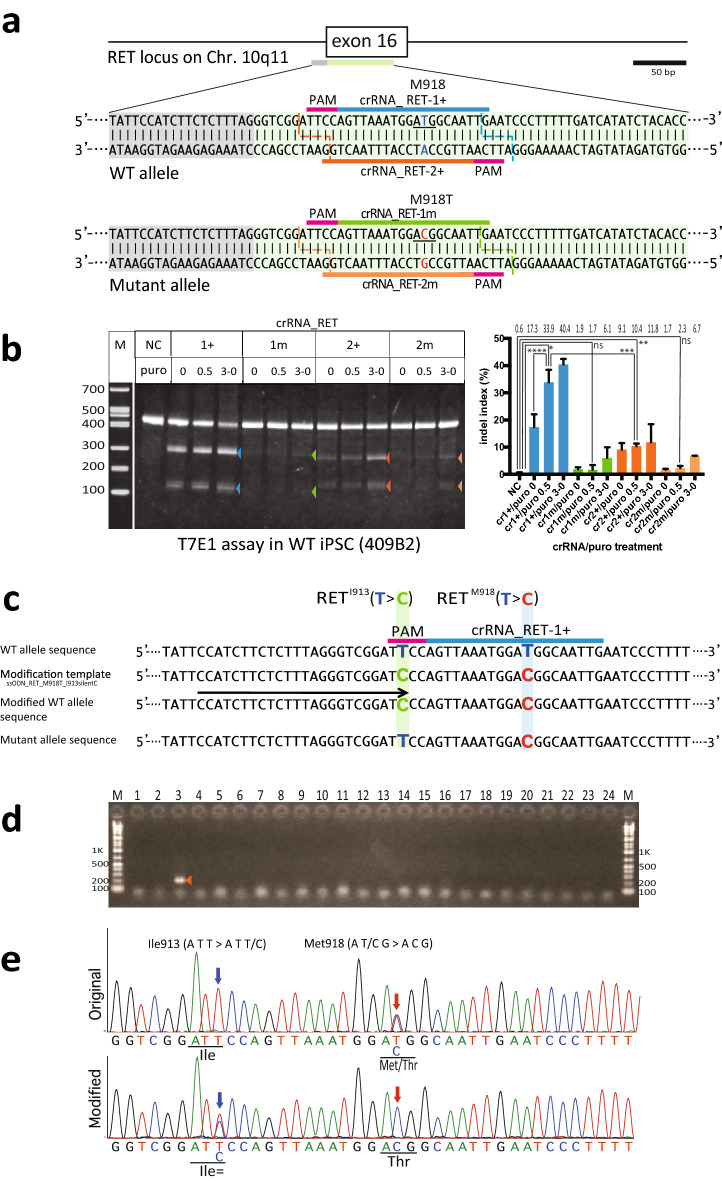
Single-nucleotide substitution of the *RET* wild-type sequence in MEN2B iPSCs. (**a**) Human *RET* locus containing the MEN2B mutation and crRNA of *As*Cas12a_RR for the mutation. Top: exon 16 of the *RET* locus. Middle: the wild-type (WT) allele sequence. Blue letters indicate the wild-type nucleotide at Met918 (underlined). Bottom: the mutant allele sequence. Red letters indicate the single missense mutation caused by a T>C substitution, producing a Met918Thr substitution (underlined). Coloured lines indicate the guide sequence template for the crRNA. The pink line indicates the *As*Cas12a_RR PAM. Coloured dashed lines indicate the sites cleaved by *As*Cas12a_RR with the corresponding crRNA. (**b**) T7E1 assay using human wild-type iPSCs (409B2) electroporated with *As*Cas12a_RR and the different crRNAs (crRNA_RET-1 + , crRNA_RET-1 m, crRNA_RET-2 + or crRNA_RET-2 m) targeting exon 16. Left: the cropped gel images. Arrowheads indicate cleaved bands. The full-length gels are presented in Supplementary Figure S7. Right: statistical analysis of the cleavage activity and specificity of *As*Cas12a_RR with the crRNAs following selection with different concentrations of puromycin. (**c**) HDR-mediated editing for generating artificial homozygous MEN2B using *As*Cas12a_RR with crRNA_RET-1 + selectively targeting *RET*_Met918 in the wild-type allele. (**d**) SNMD-PCR analysis of the first round of screening. The cropped gel image is shown here. The arrowhead indicates positive PCR amplicon (202 bp). The full-length gel is presented in Supplementary Figure S8. (**e**) Sequencing of the original and modified MEN2B iPSCs (FB4-14). Top: original sequence of *RET* exon 16 with a T > C substitution in the MEN2B mutant allele. Bottom: the modified *RET* sequence. The T > C substitution resulting in a homozygous Met > Thr substitution. Red and blue arrows indicate the positions of the pathogenic SNV and the single-nucleotide marker, respectively. Underlining indicates the codons affected by the editing. A more detailed explanation is provided in the “Extended Figure Legends” in the Supplementary Information.

We established FB4-14 human iPSCs from a patient with MEN2B using a Sendai viral vector protocol^43^. We then confirmed that the FB4-14 cells exhibited an embryonic stem cell-like morphology and expressed pluripotent gene markers, indicating that they were authentic iPSCs (Supplementary Fig. S2 and X7). To identify possible target sites for *As*Cas12a around the SNV of interest, we first searched for PAMs recognised by wild-type *As*Cas12a or the RR and RVR variants, which recognise TYCV and TATV PAMs, respectively^33,34^. We identified two PAM sites for the RR variant (TYCV, Y = C/T, V = A/C/G): TTCC, located 12-bp upstream of the target nucleotide on the sense strand, and TTCA, located 7-bp upstream of the target nucleotide on the antisense strand (Fig. 2a, magenta lines). Based on this information, we designed two pairs of crRNAs: crRNA_RET-1 + and crRNA_RET-1 m, and crRNA_RET-2 + and crRNA_RET-2 m, which contain guide sequences that specifically recognise wild-type and mutant target sequences, respectively (Fig. 2a).

To test the cleavage activity and target-recognition specificity of *As*Cas12a_RR using these crRNAs, we performed a T7E1 assay using 409B2 human iPSCs carrying the wild-type *RET* sequence in the target site (Fig. 2a, middle). The crRNAs for the wild-type sequence (crRNA_RET-1 + and crRNA_RET-2 +) each exhibited significant cleavage activity towards the wild-type target sequence (Fig. 2b; P < 0.0001 and P = 0.0246, respectively). By contrast, the crRNAs for the mutant sequence (crRNA_RET-1 m and crRNA_RET-2 m) showed extremely weak activity (Fig. 2b; P > 0.9999 for both). A more accurate ICE analysis showed no significant activity of the crRNAs on the WT allele (Supplementary Fig. X2a). These results indicate that the crRNAs for the mutant sequence do not have significant, if any, activity on the WT allele”.

However, the observed cleavage activity of *As*Cas12a_RR in conjunction with crRNA_RET-1 + was significantly higher than that with crRNA_RET-2 + (Fig. 2b; P < 0.0001, Supplementary Fig. X2a). Puromycin treatment further promoted the cleavage activity of *As*Cas12a_RR with crRNA_RET-1 + (Fig. 2b; P < 0.0001).

To test the applicability of the method to carry out allele-specific single-nucleotide substitution in human iPSCs, we attempted to replace the wild-type nucleotide (*RET*: c.2753T) at the Met918 site in the wild-type allele in FB4-14 MEN2B-iPSCs (Fig. 2c). Following electroporation of the pY211-puro vector, which expresses *As*Cas12a_RR and crRNA_RET-1 + (Fig. 2c. blue line), and a ssODN modification template (ssODN_RET_M918T_I913silentC) carrying both a variant nucleotide at Met918 and a single-nucleotide marker at Ile913 (Fig. 2c, red C and light green C, respectively) into FB4-14 cells, we conducted SNMD-PCR screening. Overall, 12/384 clones were positive for the substitution (Fig. 2c, d and GE1 in Table 1). Direct sequencing of the target sequence revealed that 7/12 clones contained the wild-type allele-specific introduction of the mutant nucleotide at the target site (T > C substitution resulting in the Met918Thr substitution; Fig. 2e, red arrow), along with the single-nucleotide marker (T > C substitution leading to a silent mutation at Ile913; Fig. 2e, blue arrow). The HDR efficiency was 1.8% (Table 1).

**Table 1:** One-SHOT and scarless One-SHOT gene editing (GE) experiments.

Gene editing #	Cell	Genotype (phenotype) Original → Destination	No. of total picked clones (TC)	No. of 1st screening- passed clones (SNMD- PCR)	No. of 2nd screening- passed clones (sequencing)	1st/TC (%)	2nd/1st (%)	2nd/TC (%)
GE1	FB4-14	RET^M918T/+^ → RET^M918T/M918T; I913_silentC/+^ (MEN2B^a^ → MEN2B homo with SN Marker)	384	12*	7	3.1	58.3	1.8
GE2	FB4-14	RET^M918T/+^ → RET^+/+; I913_silentC/+^ (MEN2B^a^ → MEN2B revertant with SN Marker)	344	17*	11	4.9	64.7	3.2
GE3	FB4-14	RET^M918T/+^ → RET^+/+; I920_silentC/+^ (MEN2B^a^ → MEN2B revertant with SN marker)	336	30*	19	8.9	63.3	5.7
GE4	FB4-14	RET^M918T/+^ → RET^+/+^ (MEN2B^a^ → MEN2B scarless revertant)	240	44**	5	18.3	11.4	2.0
GE5	B117-3	COL7A1^G2138X/+; 3,591 del.13, ins. GG/+^ → COL7A1^+/+; 3,591 del.13, ins. GG/+^ (DEB^b^ → DEB scarless revertant)	80	18**	6	22.5	33.3	7.5

After electroporation of the AsCas12a_RR expression vector and the ssODN template into the cells, the crude DNA samples from the single-cell derived colonies that expanded on the master plates were subjected to SNMD-PCR in the first screening round. For positive screening, colonies with amplifiable 150–200-bp fragments from the SNMD-PCR primer pair were the intended-clone candidates (GE1-3). For negative screening, colonies lacking PCR amplification were the intended-clone candidates (GE4 and 5). In the second screening round, we directly read the sequences around the target site of the DNA fragments amplified by Tks Gflex DNA polymerase in each sample.

*silentC* a silent mutation generated by replacement with a cytidine for SN marker.

^a^Multiple endocrine neoplasia type 2B.

^B^Dystrophic epidermolysis bullosa.

*Positive screening results.

**Negative screening results.

We then searched for off-target sequences corresponding to the target sequence using the web tool CHOPCHOP v2^44^ and detected no indels in either of the predicted two off-target sites by Sanger sequencing (Table 2, GE1) and by AmpliSeq (Supplementary Table X4). These results indicated that the One-SHOT method could be used to replace a single nucleotide in an allele-specific manner while minimising off-target effects. As in the preliminary experiment, direct sequencing analysis around the target sites revealed no duplication events in the unintended gene-edited clones, suggesting that most of the intended gene-edited clones had clonally proliferated (Supplementary Fig. S3, GE1).

**Table 2:** Off-target effects of AsCas12a_RR in gene editing experiments 1–4 (GE1-GE4).

Sample	Site	Genomic location	No. of mis- matches	Sequence^a^ (including mismatches)	Indel ratio (%)^b^
Original	RET exon 16 target1	chr10: 43121953	0	TTCCAGTTAAATGGATGGCAATTG	
GE1	Off-target 1	chr15: 91512242	3	TTCCcGTTAAtTGGtTGGCAATTG	0/7 (0%)
GE1	Off-target 2	chr4: 128631982	3	TTCCAcTTAAATGcATGGCAtTTG	0/7 (0%)
GE2	Off-target 1	chr15: 91512242	3	TTCCcGTTAAtTGGtTGGCAATTG	0/11 (0%)
GE2	Off-target 2	chr4: 128631982	3	TTCCAcTTAAATGcATGGCAtTTG	0/11 (0%)
GE3	Off-target 1	chr15: 91512242	3	TTCCcGTTAAtTGGtTGGCAATTG	0/11 (0%)
GE3	Off-target 2	chr4: 128631982	3	TTCCAcTTAAATGcATGGCAtTTG	0/11 (0%)
GE4	Off-target 1	chr15: 91512242	3	TTCCcGTTAAtTGGtTGGCAATTG	0/5 (0%)
GE4	Off-target 2	chr4: 128631982	3	TTCCAcTTAAATGcATGGCAtTTG	0/5 (0%)

After amplifying the off-target candidates (predicted by CHOPCHOP v2) from the intended gene-edited iPSC clones, we directly read the sequences around the candidate sites after Sanger sequencing with specific primers.

^a^Underline indicates the PAM of the AsCas12a_RR variant. Lower letters indicate mismatched bases in the off-target candidates, as compared with the original target sequence.

^b^Number of indel clones relative to the number of analysed clones.

### Allele-specific single-nucleotide repair of a pathogenic *RET* variant

**Figure 3:**
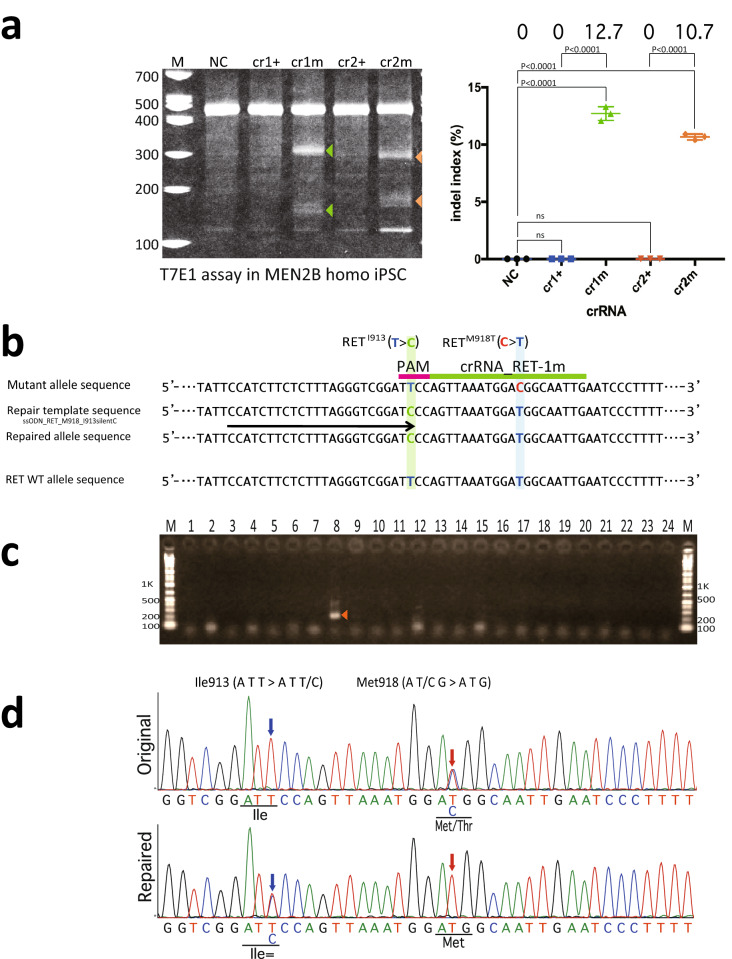
Single-nucleotide substitution in the human mutant allele in MEN2B iPSCs. (**a**) T7E1 assay in human artificial homozygous MEN2B iPSCs (FB4-14 homologous cells, generated using the scheme shown in Fig. 2) electroporated with the pY211-puro expression vector, which expresses *As*Cas12a_RR plus crRNAs. Left: The cropped gel image of the T7E1 assay. *As*Cas12a_RR with crRNA_RET-1 m and crRNA_RET-2 m, designed to target the mutant allele, exhibited significant cleavage activity on the mutant allele. Arrowheads indicate cleaved bands. *M* marker, *NC* negative control. The full-length gel is presented in Supplementary Figure S9. Right: statistical analysis of the cleavage activity and specificity. Bars indicate the mean and S.E.M. from experiments performed in biological triplicate. Data were analysed by one-way ANOVA with Tukey’s multiple comparison test. *ns* not significant. (**b**) HDR-mediated editing for repair of the MEN2B genome. The light green line indicates the target of crRNA_RET-1 m. The pink line indicates the *As*Cas12a_RR PAM. Red, blue and light green letters denote the pathogenic SNV, the wild-type nucleotide and the single-nucleotide marker, respectively. The pale blue and green columns indicate the positions of the pathogenic SNV and the single-nucleotide marker, respectively. The arrow indicates the position of the forward primer for the SNMD-PCR. (**c**) First round of SNMD-PCR screening. The arrowhead indicates the expected amplicon (202 bp). The full-length gel is presented in Supplementary Figure S10. (**d**) Sequencing chromatogram for the original and repaired MEN2B iPSCs. Top: the original sequence of *RET* exon 16 with a T>C substitution in the mutant allele. Bottom: the repaired *RET* sequence. Red and blue arrows indicate the positions of the pathogenic SNV and the single-nucleotide marker, respectively. Underlining indicates the codons affected by the editing.

To repair the pathogenic SNV (*RET*: c.2753T>C) in the mutant allele in FB4-14 cells, we first tested the cleavage activity and target recognition specificity of *As*Cas12a_RR using crRNA_RET-1 m and crRNA_RET-2 m (Fig. 2a) in a homozygous MEN2B iPSC line with mutations in *RET* exon 16 in both alleles (GE1-9, genotype: *RET*^Met918Thr/Met918Thr^; *RET*^Ile913 silentC/+^) (Fig. 2e, bottom). The T7E1 assay confirmed that the MEN2B target sequence was selectively cleaved by *As*Cas12a_RR with either crRNA_RET-1 m or crRNA_RET-2 m, but not with crRNA_RET-1 + or crRNA_RET-2 + (Fig. 3a). The ICE analysis revealed that only the *As*Cas12a_RR with crRNA_RET-1 m exhibited strong cleavage activity on the target sequence (Supplementary Fig. X2b), therefore we selected the crRNA_RET-1 m for use in subsequent experiments.

We then carried out One-SHOT repair in FB4-14 cells using *As*Cas12a_RR with crRNA1m and a ssODN repair template containing a repair nucleotide at Met918 and a single-nucleotide marker at Ile913 (Fig. 3b, T in blue and C in light green, respectively). Subsequent SNMD-PCR screening showed that 17/344 clones were positive (Fig. 3c, GE2 in Table 1), while direct sequencing confirmed that 11 of the positive clones contained the introduced wild-type nucleotide at the target site (C > T substitution), leading to a Thr918Met substitution (repair) (Fig. 3d, red arrow). These clones also contained the single-nucleotide marker (T > C substitution), leading to a silent mutation at Ile913 (Fig. 3d, blue arrow). The overall HDR efficiency was 3.2% (Table 1, GE2), and we detected no off-target effects by Sanger sequencing (Table 2, GE2) and by AmpliSeq (Supplementary Table X4). As in the preliminary experiment, direct sequencing analysis around the target sites revealed no duplication events in the unintended gene-edited clones, suggesting that most of the intended gene-edited clones had clonally proliferated (Supplementary Fig. S3, GE2).

### Allele-specific single nucleotide repair of a pathogenic variants in *RET* and *COL7A1* without a single-nucleotide marker

**Figure 4:**
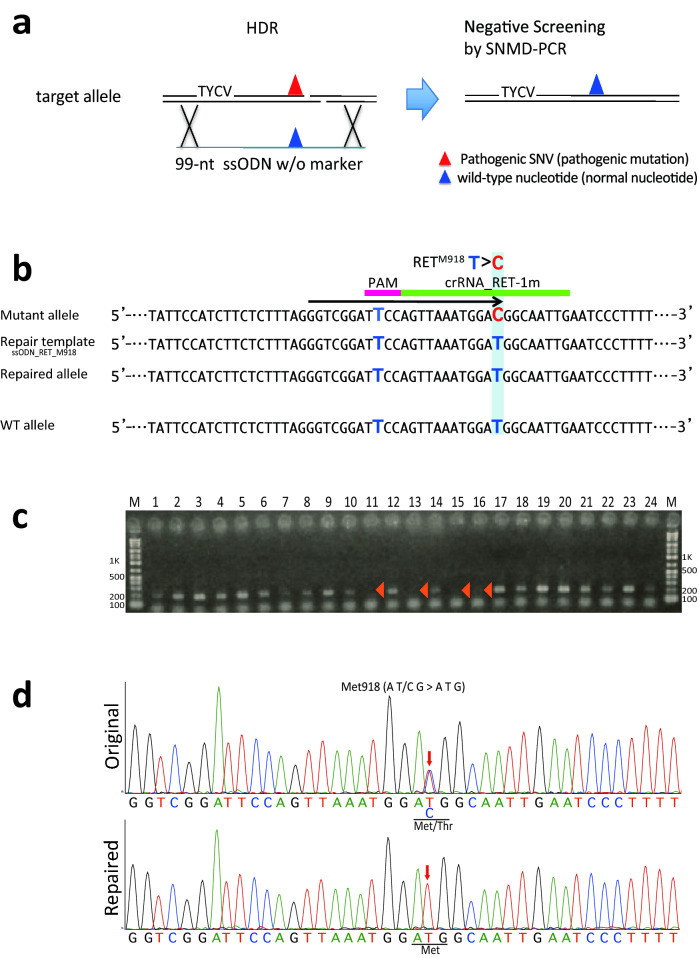
Repairing the pathogenic single-nucleotide variant (SNV) with scarless One-SHOT. (**a**) Scarless One-SHOT. The target site (left panel) on the pathogenic allele was repaired using the One-SHOT approach with a normal ssODN carrying no single-nucleotide markers. In this case, the intended gene-edited clones were identified by negative screening for the pathogenic SNV because the repaired, ex-pathogenic allele had lost the pathogenic SNV (right panel). Red and blue triangles indicate the pathogenic SNV and the wild-type nucleotide, respectively. (**b**) HDR-mediated scarless editing repair of the MEN2B genome using *As*Cas12a_RR with crRNA_RET-1 m selectively targeting the *RET*_Met918Thr site in the mutant allele (see Fig. 3b for the strategy). The PCR primer position is indicated by the black arrow. (**c**) Negative screening by SNMD-PCR as the first round of screening. The arrowheads denote the intended clones from this screening (188 bp). The full-length gel is presented in Supplementary Figure S11. (**d**) Sequencing chromatograms for the original and repaired MEN2B iPSCs. Top: the original sequence of *RET* exon 16 with a T>C substitution in the MEN2B mutant allele. Bottom: the repaired sequence of *RET* exon 16 (C>T substitution). Red arrows indicate the position of the pathogenic SNV. Underlining indicates the codons affected by the editing.

We next investigated whether the One-SHOT method could be used to repair the pathogenic SNV in *RET* without including the single-nucleotide marker, which would achieve true scarless repair. We therefore performed One-SHOT repair in the FB4-14 cells using *As*Cas12a_RR, crRNA_RET-1 m and the ssODN repair template with only a wild-type nucleotide at Met918. In the subsequent SNMD-PCR screening for the pathogenic SNV, no amplicons were obtained from repaired clones because the pathogenic SNV was lost from the mutant allele (Fig. 4a). Overall, we identified 44 negative clones by SNMD-PCR screening for the pathogenic SNV, and direct sequencing revealed that 5/44 carried only the wild-type nucleotide at Met918 (Fig. 4c,d and GE4 in Table 1). In this experiment, the overall HDR efficiency was 2.0% (Table 1, GE4), and no indels were detected in the two predicted off-target sites by Sanger sequencing (Table 2, GE4) and by AmpliSeq (Supplementary Table X4).

We next attempted to perform scarless repair of a pathogenic SNV in iPSCs derived from a patient with DEB to confirm the applicability of the approach for other hereditary diseases. We generated iPSCs from a patient with DEB (autosomal recessive compound mutation: *COL7A1*^p.Gly2138Ter/+^; *COL7A1*^c.3591del13insGG/+^) and aimed to substitute the pathogenic SNV (c.6412G > T (p.Gly2138Ter)) in exon 78 (Supplementary Fig. S4a,b). Scarless One-SHOT using *As*Cas12a_RR with crRNA_COL7A1-1 m plus the repair template scarlessly repaired the pathogenic SNV in the mutant allele (Supplementary Fig. S4c,d,e and GE5 in Table 1), with a substitution rate of 7.5%. No indels were detected in the seven predicted off-target sites (Supplementary Table S2, Supplementary Table X4). Unlike the scarless One-SHOT for *RET*_Met918Thr in FB4-14 cells (Fig. 4, GE4 in Table 1), identical sequences (~ 22%) within the target site were observed among the unintended gene-edited clones, suggesting that these clones were likely duplicated (Supplementary Fig. S3, GE5).

## Discussion

Many hereditary human diseases are caused by single-nucleotide mutations. These single-base alterations have the potential to drastically alter protein structure and function. Although most single-nucleotide mutations are completely harmless (silent), repair of pathogenic SNVs would significantly improve the quality of life and life expectancy of patients with hereditary diseases. Thus, in the present study, we investigated whether we could achieve scarless repair of pathogenic SNVs in pluripotent stem cells from patients with two different types of hereditary disease: MEN2B and DEB. More importantly, we aimed to carry out the repairs in a single step.

Using the One-SHOT approach developed in this study, we successfully repaired a *RET* gene SNV in MEN2B iPSCs with the addition of a single-nucleotide selective marker in a single step. We then confirmed that the same technique could be used to carry out scarless repair in MEN2B- and DEB-specific iPSCs without the need for the single-nucleotide marker. Scarless repair, where no trace of gene editing is left around the target sequence, is the goal of any gene editing technique because it safely repairs mutations in non-coding genomic regions without any secondary effects. In contrast, the inclusion of marker sequences during gene editing can have downstream effects. Such secondary effects include the introduction of non-coding SNVs to cryptic splice sites, causing abnormal RNA splicing^45,46^, and mutations that introduce a premature termination codon, resulting in unstable mRNA^46^. Non-coding mutations affecting regulatory elements can also interfere with gene regulation through loss of function, resulting in reduced gene expression, or gain of function, resulting in gene mis- or overexpression^47,48^. Therefore, scarless repair is crucial for maintaining genome integrity and preventing unknown secondary effects in the target gene.

Several other methods of pathogenic SNV repair have been developed, including CORRECT^26,27^ and MhAX^49^. However, all currently available methods have inherent obstacles to achieving scarless SNV repair in a fast and error-free manner. To overcome some of these obstacles, we used the *As*Cas12a nuclease, which has high-fidelity target-recognition^35,36^, circumventing the need for a blocking base to inhibit re-cutting, as is required in other methods^26,27^. We also performed SNMD-PCR-based negative screening for the pathogenic SNV, which easily detects candidate clones containing the intended alteration. As a result of these modifications, we achieved absolute scarless editing of the *RET* and *COL7A1* SNVs (see Fig. 4, Supplementary Fig. S4 and GE4 and GE5 in Table 1). Another advantage of the *As*Cas12a nuclease was the ability to carry out SNV repair in a single step because only one round of HDR is required for gene editing (Fig. 1). The One-SHOT method was used to repair the SNVs in *RET* and *COL7A1* within a 3-week period with sufficient efficiency for hand-picking. In contrast, other methods can take up to 2–3 months to generate the intended gene-edited clone because two rounds of HDR/MMEJ may be required^26,27,49^. However, similar to our approach, the CORRECT method can achieve scarless single-nucleotide substitution, thus ensuring high sequence fidelity around the target site in gene-edited cells (Fig. 4 and Supplementary Fig. S4). Conversely, MhAX leaves a silent single nucleotide mutation around the target site for use in screening^49^. Another difference is that the dsDNA template in MhAX can be randomly integrated into the genome (outside of the target site) by non-homologous end joining^50^, whereas the ssODN templates used for One-SHOT/scarless-One-SHOT and CORRECT approaches are not randomly integrated^51^. Thus, the One-SHOT method developed for SNV repair in the current study appears to have several advantages over currently available methods (see Supplementary Table S3).

In the CORRECT procedure, the cut-to-mutation distance (the distance between the CRISPR-*Sp*Cas9 cleavage site and the blocking mutation) is a crucial factor for HDR efficiency and zygosity determination^26,27^. We therefore searched for more appropriate sites for the single-nucleotide markers by first comparing the efficacies of three single-nucleotide markers set in different positions around the target site using a PCR-restriction fragment length polymorphism (RFLP) assay^52^. We found that two of the markers showed similar HDR-specific cleavage activity, while no cleavage activity was detected for the third marker (Supplementary Information and Supplementary Fig. S5), suggesting that Ile920 could be used as an alternative single-nucleotide marker. Testing of HDR efficiency in FB4-14 cells following One-SHOT repair using the alternative marker again confirmed that the single-nucleotide substitutions in the gene-edited clones were effectively detected by positive screening using SNMD-PCR for a single-nucleotide marker (Supplementary Information and Supplementary Fig. S5). We do note, however, that the efficiency of identification might depend on the position of the single-nucleotide marker and the primers used for SNMD-PCR.

Despite our success in repairing the pathogenic SNVs in a single step, the study has several limitations. The One-SHOT method only requires one PCR run, thereby reducing the time and cost compared with standard PCR–RFLP screening-based methods, which require up to three steps^52,53^. However, we found that false-positive clones are included in the population after the first SNMD-PCR screen (Supplementary Fig. X8). Therefore, we are currently designing a simple way to discriminate false clones from authentic clones using a PCR-based procedure. We also noted that the gene-edited cell lines generated by One-SHOT are not always clonal. This situation arises because high cell densities occur in the culture during puromycin selection (2 days) and in the recovery culture (1–2 days) prior to clonal expansion. However, assessment of our data suggests that a 1-day recovery culture and sufficient single-cell suspension at the reseeding stage can prevent duplication and ensure clonal establishment of the gene-edited cells. Using the current protocol, we estimate that the HDR substitution rate is 1.8–7.5%. While this is sufficient to permit a hand-picking cloning protocol, it is lower than that achieved by Cas12a in fertilised eggs from model animals^39,54^ We hoped to improve this rate by combining One-SHOT with other procedures based on alternative principles, such as introducing a blocking base into the repair template^26,27^ and/or using HDR/NHEJ modification compounds^38,63–67^. We have examined whether the modification compounds can promote HDR, however, the compounds examined in this study had no HDR-promoting effects in our experimental system (Supplementary Fig. X4).

It is important to emphasise, though, that the procedure depends on high-fidelity target recognition by the site-specific nuclease. Thus, the only enzymes appropriate for the One-SHOT procedure include high-fidelity variants of engineered *Sp*Cas9^55–58^ or naturally high-fidelity Cas9 orthologues^59–62^. Finally, while we confirmed the expression of pluripotency markers in the gene-edited clones (data not shown), we next aim to carry out functional analyses to confirm the differentiation potential of the repaired cells. Therefore, further work is needed to fine-tune the protocol and to confirm differentiation potential and functionality of the proteins in the corrected cell populations.

To increase the reliability of the One-SHOT method, it is important to show the robustness of One-SHOT and the fidelity of the repair. In order to demonstrate these issues, we performed targeted NGS-based deep AmpliSeq analysis of the target sequence. With regard to repair fidelity, the AmpliSeq analysis showed that accurate single-nucleotide substitutions were achieved by HDR that were faithful to the ssODN template and occurred at sufficient frequency (Supplementary Fig. X3a-c, 2.45–5.44%). These results suggest that the method has good repair fidelity. With regard to the robustness of One-SHOT method, the AmpliSeq analysis confirmed that single-nucleotide substitutions were reliably performed under various conditions (different targets, different templates), (Supplementary Fig. X3, Supplementary Table X4), suggesting that our genome editing method is robust.

Quality control for the gene-edited cells is quite important for research and industrial use. Here, we performed four tests (clonal purity, on-/off-target effects, karyotyping, and random integration of the plasmid vector) as a form of quality control for the gene-edited cells generated via the One-SHOT method. Our results confirmed the clonal purity of the cells (Supplementary Fig. X1) and showed that off-target effects were negligible (Supplementary Fig. X3, Supplementary Table X4). However, karyotyping (Supplementary Fig. X5) and plasmid integration analysis (Supplementary Fig. X6) uncovered low-frequency chromosomal abnormalities and low-frequency plasmid integration events. Analysis of the literature suggests that these are not problems unique to the One-SHOT method, and are also observed in other genome editing processes^70,71^. These findings suggest that strict quality control is required when creating and using gene-edited cells, depending on the purpose (basic research, drug discovery platform, cell transplantation).

In summary, repair of pathogenic SNVs causing hereditary diseases in humans using site-specific nucleases has been relatively difficult to date because of insufficient target recognition accuracy by wild-type *Sp*Cas9. By using *As*Cas12a as the genome editing tool, we successfully achieved repair of two different pathogenic SNVs in a single step. The One-SHOT procedure developed in this study is simple, fast, low-cost, efficient, and broadly applicable to genome-editing applications in basic biological and biomedical research. We anticipate that One-SHOT will become a powerful procedure for generating isogenic cells from disease-specific iPSCs and for repairing genomic mutations in somatic cells, germ-line stem cells, and iPSCs.

## Materials and methods

### FB4-14 and B117-3 iPSC clones

FB4-14 cells (MEN2B-specific iPSCs) were used in GE experiments 1–4 (GE1–4). The cells were generated from a 40-year-old male patient who carried an autosomal dominant mutation at the MEN2B disease locus. The disease is caused by a mono-allelic SNV (*RET*: c.2753T>C) in the tyrosine kinase domain of the *RET* gene at codon 918 in exon 16, resulting in a Met918Thr substitution^7^. In GE5, B117-3 cells (DEB-specific iPSCs) were used. These were generated from a 3-year-old male patient who carried DEB autosomal recessive compound mutations within the *COL7A1* gene. These mutations consisted of a mono-allelic SNV (*COL7A1*: c.6412G > T) at codon 2,138 in exon 78, causing a nonsense mutation (p.Gly2138Ter), along with a mono-allelic indel (c.3591del 13insGG) resulting in a frame-shift in exon 27. For the initial T7E1 assay, wild-type iPSCs (409B2; RIKEN BRC, Japan; clone number HPS0076) were used. iPSC clones FB4-14 and B117-3 exhibited typical iPSC properties (Supplementary Figs. S2, S4a, and X7).

### Transfection of iPSCs

iPSCs were electroporated with a specific pY211-puro vector and ssODN. Briefly, 1 × 10^6^ cells were resuspended in 100 µl of OptiMEM (Thermo Fisher Scientific, USA) containing 10 µg of pY211-puro and 15 µg of ssODN (99 nucleotides, PAGE-purified; Sigma-Aldrich, USA). The cells were then electroporated (transfer pulse, 20 V; pulse length, 50 ms; number of pulses, 5) in a 2-mm gap-size cuvette using a Super Electroporator NEPA21 Type 2 instrument (NEPA GENE, Japan). Following electroporation, cells were transferred to Matrigel-Growth Factor Reduced (GFR)-coated 24-well plates containing mTeSR1 plus CloneR medium (STEMCELL Technologies, Canada) and cultured for 16 h. The cells were then cultured in mTeSR1 plus CloneR medium containing puromycin (0.5 µg/ml) for 48 h, and subsequently transferred back into mTeSR1 plus CloneR medium for 1–2 days to recover. After recovery, the cells were singularised using TrypLE (Gibco/Thermo Fisher Scientific, USA) and seeded at a low density (500–1,000 cells per 100 mm plate) in mTeSR1 plus Clone R medium for cloning.

### Genotyping of single-cell-derived clones by SNMD-PCR and sequencing

To aid in the genotyping of single-cell-derived clones, ssODN templates for HDR were designed to introduce a single-nucleotide marker (a silent mutation) independent of the pathogenic SNV for SNMD-PCR screening. Genome-edited, single-cell-derived iPSC clones were cultured in master plates (Matrigel-GFR-coated 100-mm plates with 100 square grids (PetriSticker); Diversified Biotech Inc., USA) for 4 days in mTeSR1 plus CloneR medium, followed by 3 days in unsupplemented mTeSR1. The central portion of each colony (approximately 25–33%) was manually picked under a microscope^68^ and directly transferred to a PCR tube containing 10 µl of lysis buffer (DirectPCR Lysis Reagent Cell, Viagen Biotech, USA). The remaining colonies on the master plates were maintained for 2–3 days under the same conditions.

To identify the HDR-targeted clones by SNMD-PCR, the genomic regions surrounding the target loci were amplified using HiDi DNA polymerase^69^ (myPOLS Biotec GmbH, Germany) and the corresponding single-nucleotide marker-specific or allele-specific primer pair (Supplementary Table S1). Amplicons were analysed by 2% agarose gel electrophoresis. Gene Ladder Wide I (Nippon Gene, Japan) was used as DNA molecular size marker. The presence of the single-nucleotide marker introduced by HDR and the zygosity of the pathogenic variants in the clones were confirmed by Sanger sequencing. For these assays, 450–500-bp amplicons around the gene-edited locus were PCR-amplified using the specific primer pair and Tks Gflex DNA polymerase (Takara Bio, Japan). Sequencing was performed using a BigDye Terminator v3.1 Cycle Sequencing Kit (Thermo Fisher Scientific, USA).

### One-SHOT procedure

To perform allele-specific single-nucleotide substitution at the pathogenic SNV in the human iPSCs, we developed the One-SHOT repair method using high-fidelity RNA-guided site-specific nuclease *As*Cas12a. To carry out the repair, 1 × 10^6^ iPSCs were electroporated with a pY211-puro vector expressing *As*Cas12a_RR, crRNA corresponding to the target sequence and the puromycin resistance gene. In the positive-screening protocol for standard genome editing (standard One-SHOT), a ssODN was used to introduce the intended mutation (M) and a single-nucleotide marker (S) (the MS template). Cells transiently expressing *As*Cas12a-crRNA-puro^R^ were treated with puromycin, and surviving cells were enzymatically singularised and plated at a low density (500–1,000 cells per 100-mm plate) to permit growth of the single-cell-derived clones. The presence of the single-nucleotide marker was detected by SNMD-PCR and confirmed by Sanger sequencing. In the case of the negative-screening protocol for scarless genome editing (scarless One-SHOT), a ssODN that only contained the intended mutation (the M template) was introduced. The presence of the M mutation was negatively detected by SNMD-PCR and confirmed by Sanger sequencing. The efficiency of introducing the intended mutation by One-SHOT was determined by Sanger sequencing of the established and expanded clones. A detailed explanation of each step is provided in the Supplementary Information (Extended Materials and Methods).

### Statistical analyses

GraphPad Prism version 7 (GraphPad Software Inc., USA) was used for all statistical analyses. An analysis of variance (ANOVA) was performed on all data, followed by Tukey’s multiple comparisons test for the T7E1 assays and ICE analysis, or Sidak’s multiple comparisons test for the PCR–RFLP assay and Clonal Purity analysis.

### Ethical statement

All experiments and methods were performed in accordance with relevant guidelines and regulations. All experimental protocols were approved by the Fukushima Medical University Institutional Review Board. All experimental procedures involving human samples were approved by the following ethics committees: the Human Genome and Gene Analysis Research Committee at Fukushima Medical University (approval number 2186), the Epidemiological and General Research Committee of the Faculty of Life Science, Kumamoto University, the Human Genome and Gene Analysis Research Committee of the Faculty of Life Sciences, Kumamoto University, and the Clinical Research and Advanced Medical Technology Committee, Kumamoto University (approval numbers 318, 153 and 1,018, respectively). Blood samples (10 ml) were collected from the two patients involved in this study, and T-cells were isolated using Ficoll-Paque PREMIUM density gradient media (GE Healthcare). Informed consent was obtained from the participant or the participant’s parents prior to the study.

## Supplementary information

Supplementary file1

## Data Availability

The datasets generated during and/or analysed during the current study are available from the corresponding author on reasonable request.
