# Supplementary material for: Rapid repair of human disease-specific single-nucleotide variants by One-SHOT genome editing
Source: Sci Rep. 2020 Aug 18;10:13927. doi: 10.1038/s41598-020-70401-7 (PMC7435196; doi:10.1038/s41598-020-70401-7)
Supplement: Supplementary file 1 — Supplementary file1 [file 41598_2020_70401_MOESM1_ESM.pdf]

Supplementary Information

Rapid repair of human disease-specific single-nucleotide variants by One-SHOT genome editing

Names of Authors

Yuji Yokouchi[1,2*], Shinichi Suzuki[3], Noriko Ohtsuki[1,2], Kei Yamamoto[1,2], Satomi Noguchi[1,2], Yumi Soejima[3], Mizuki Goto[3,4], Ken Ishioka[5], Izumi Nakamura[1,2], Satoru Suzuki[6], Seiichi Takenoshita[7] and Takumi Era[1,2,3]

Names of Institutions at which the work was done

[1]Pluripotent Stem Cell Research Unit in Department of Thyroid and Endocrinology, School of Medicine, Fukushima Medical University, Fukushima, Japan;

[2]Department of Thyroid and Endocrinology, School of Medicine, Fukushima Medical University, Fukushima, Japan;

[3]Department of Cell Modulation, Institute of Molecular Embryology and Genetics (IMEG), Kumamoto University, Kumamoto, Japan;

[4]Department of Dermatology, Faculty of Medicine, Oita University, Yufu, Japan;

[5]Department of Microbiology, School of Medicine, Fukushima Medical University, Fukushima, Japan;

[6]Office of Thyroid Ultrasound Examination Promotion, Radiation Medical Science Center for the Fukushima Health Management Survey, Fukushima Medical University, Fukushima, Japan;

[7]President, Fukushima Medical University, Fukushima, Japan

Correspondence:

Yuji Yokouchi, PhD, Pluripotent Stem Cell Research Unit in Department of Thyroid and Endocrinology, School of Medicine, Fukushima Medical University, 1 Hikariga-oka, Fukushima 960-1295, Japan.

Phone: +81-24-547-1915; e-mail: yokouchy@fmu.ac.jp

Extended Results

Effect of cut-to-mutation distance on homology-directed repair (HDR) efficacy. To examine the effect of the cut-to-mutation distance on HDR efficacy, we searched for more appropriate sites for the single-nucleotide markers by comparing the efficacies of three single-nucleotide markers in different locations around the target site using a polymerase chain reaction-restriction fragment length polymorphism (PCR-RFLP) assay. For these single-nucleotide markers, we selected the third base of each codon (Ile913, Ile920 or Asp933) around the *RET*_Met918 site (Supplementary Fig. S5a). These single-nucleotide markers (T>C substitutions) produce silent mutations that generate new restriction enzyme recognition sites that can be digested with *Alw*I, *Taq*αI or *Afl*III, respectively (Supplementary Fig. S5a, underlines). We then performed One-SHOT repair assays in FB4-14 cells using *As*Cas12a_RR, crRNA_RET-1m, appropriate single-stranded oligodeoxynucleotide (ssODN) repair templates and each of the individual single-nucleotide markers. We then measured HDR-specific cleavage activity using a PCR-RFLP assay (Supplementary Fig. S5a, b). The HDR-specific cleavage activity for the Ile920 single-nucleotide marker was similar to that for the Ile913 single-nucleotide marker (21.4 % vs 19.4 %, Supplementary Fig. S5b), while no cleavage activity was detected for the Asp933 single-nucleotide marker (Supplementary Fig. S5b), suggesting that Ile920 could be used as an alternative single-nucleotide marker.

We next performed allele-specific, single-nucleotide repair in the FB4-14 cells using *As*Cas12a_RR with crRNA_RET-1m and the ssODN_Ile920_silentC marker (Supplementary Fig. S5c, d and GE3 in Table 1). The HDR efficiency with ssODN_Ile920_silentC was markedly higher than that observed using ssODN_Ile913_silentC (5.7% vs 3.2%; GE2 in Table 1), despite the similar levels of RFLP-detected cleavage activity shown by Ile913 and Ile920 (Supplementary Fig. S5b). We detected no indels in the two predicted off-target sites in any of the gene-edited clones (Table 2, GE3). These results again confirmed that the single-nucleotide substitutions in the gene-edited clones were effectively detected by positive screening using single-nucleotide mismatch detection (SNMD)-PCR for a single-nucleotide marker. Of note, the efficacy of identification might depend on the position of the single-nucleotide marker and the primers used for SNMD-PCR.

Additional Data

Additional Data 1. Measurement of the clonal purity of gene-edited clones.

Results:

When clones of gene-edited cells from a master plate are manually picked, a small number of colonies may be contaminated with parental cells. To directly calculate the contamination rate, we conducted AmpliSeq analysis of the gene-edited clones targeting parental cell-derived sequence. Because all of our gene editing experiments involved the creation of a homologous allele from the heterologous allele, counting the number of hetero-allelic sequences within the genomic DNA of a clone allowed calculation of the parental genome contamination rate.

The arithmetic mean of the contamination rates from five genome editing experiments ranged from 0.048%–0.5% (Supplementary Fig. X1c). We then conducted pairwise comparisons of each contamination rate with that of the baseline control (most frequent error reads) for that experiment. Our results showed that there were no significant differences among experiments (GE1, GE2, and GE3), or that the contamination rate was significantly smaller than that of the baseline control (GE4, $P < 0.0001$; GE5, $P = 0.001$). Thus, we concluded that the levels of parental cell contamination of the gene-edited clones were at or below the baseline level.

Discussion:

Using NGS-based clonal purity analysis, we confirmed that the rates of parental genome contamination of the gene-edited clones in this study ranged from 0.048%–0.5%. This contamination rate is sufficiently low to allow the use of the generated clones as a drug discovery platform. However, re-cloning may be required if they were to be used as a resource for cell therapy.

Additional Data 2. Inference of CRISPR edits (ICE) analysis.

Results:

To more accurately measure the SSN cleavage activity of the genome editing tools, ICE analysis was performed. The Sanger sequencing trace data were then processed to separate overlapping traces and calculate the total number of indels generated in the target sequence by SSN[1]. The output data showed a strong correlation with the results of the AmpliSeq analysis, indicating that ICE analysis is an inexpensive alternative to AmpliSeq analysis[1].

The SSN activities of the four genome editing tools (*As*Cas12a_RR with crRNA_RET-1+, crRNA_RET-1m, crRNA_RET-2+, or crRNA_RET-2m) at the RET_M918 site in the wild-type sequence (RET_M918 site) were 69%, 0%, 4.5%, and 0%, respectively (Supplementary Fig. X2a).

*As*Cas12a_RR with crRNA_RET-1+ or crRNA_RET-2+, designed for the wild-type sequence, showed significant cleavage activity compared with the negative control (one-way ANOVA and Turkey's multiple comparisons test; N = 4; P < 0.0001, P = 0.0004, respectively). In particular, the activity of *As*Cas12a_RR with crRNA_RET-1+ was very strong, with an activity level more than 10 times that of *As*Cas12a_RR with crRNA_RET-2+. crRNA_RET-1m and crRNA_RET-2m, designed for the mutant sequence, showed no cleavage activity in the wild-type sequence (N = 4; P > 0.999). Comparison of the cleavage activities of the two paired crRNAs (crRNA_RET-1+ and 1m, or crRNA_RET-2+ and 2m) revealed a significant difference between them (N = 4; P <0.0001, P = 0.0004, respectively).

In the mutant sequence (RET_M918T site), the observed activity of the four genome editing tools was the opposite of that observed in the normal sequence (0%, 18%, 0%, and 0%, respectively; Supplementary Fig. X2b). Specifically, crRNA_RET-1m, designed for the mutated sequence, showed significant cleavage activity (one-way ANOVA and Turkey's multiple comparisons test; N = 4; P < 0.0001), while crRNA_RET-1+ and crRNA_RET-2+, designed for the normal sequence, showed no cleavage activity (N = 4; P > 0.999). Comparison of the cleavage activities of the paired crRNAs (crRNA_RET-1+ and crRNA_RET-1m) revealed a significant difference between them (N = 4; P < 0.0001).

The activities of the six genome editing tools (*As*Cas12a_RR with crRNA_COL7A1-1+, 1m, 2+, 2m, 3+, or 3m) targeting the COL7A1_exon78 site in the wild-type allele were 22.5%, 0%, 5.5%, 0%, 0%, and 0%, respectively, with the cleavage activities of crRNA_COL7A1-1+ and 2+, which were designed for the wild-type sequence, significantly increased compared with the negative control (one-way ANOVA and Turkey's multiple comparisons test; N = 4; P < 0.0001, Supplementary Fig. X2c). crRNA_COL7A1-1m, 2m, and 3m, designed for the mutant sequence, showed no cleavage activity (N = 4; P > 0.999). Comparison of the cleavage activities of the two paired crRNAs (crRNA_COL7A1+ and 1m, or crRNA_COL7A1-2+ and 2m) revealed a significant difference between them (N = 4; P < 0.0001). These results indicate that *As*Cas12a_RR with the crRNA recognizes a single base mismatch in the target sequence and has high-fidelity site-specific cleavage activity at the target sequence, as shown in the T7E1 assay.

Additional Data 3-1. On-target effects.

Results:

To investigate the effects of genome editing on the target sequence in HDR situations in more detail, we performed AmpliSeq analysis of the target sequence in genomic DNA from disease-specific iPS

cells following introduction of a genome editing toolset for repair or modification (plasmid and ssODN, Supplementary Table X4). We then examined indels and single-nucleotide variations at the edited target site.

Our results showed that deletions occurred in the target allele in addition to single-base substitutions (Supplementary Fig. X3). In the case of modification of the wild-type allele, only the wild-type sequence was cleaved and contained deletions (**a**. GE1re. Rows 3, 6, 7, 8, 9, and 10. Sequence containing wild-type nucleotide in blue). In the case of repair of the mutant allele, only the mutant sequence was cleaved and contained deletions (**b–d**, GE2re–GE4re. Sequence containing the mutant nucleotide shown in red). We could not determine which of the sequences (mutant or wild-type) was more commonly cleaved for GE5re because the base used to distinguish between the normal/mutant cleavage site was located in the hot-spot of the indels (**e**, GE5re).

Interesting results were also obtained with respect to the abundance of the target sequence and its non-target sequence. In the case of editing with a SN-marker, the abundance of a non-target sequence containing a single nucleotide difference compared with the target was close to the theoretical value of 50% (Supplementary Fig. X3, GE1re, 52.7%; GE2re, 52.4%; GE3re, 54.3%). However, the abundance of the target sequence decreased significantly (GE1re, 18.3%; GE2re, 18.6%; GE3re, 22.2%). This indicated that only the target sequence is selectively cut.

Additional Data 3-2. Off-target effects.

Results:

To determine whether *As*Cas12a_RR with crRNA_RET-1m shows on-target cleavage activity in a HDR situation for repair of RET_M918T (SSN activity at the target sequence was confirmed by T7E1 assay and ICE analysis), three genome editing tool sets (pY211-puro-crRNA_RET-1m plus ssODNs for repair: ssODN_RET_M918_I913silentC, ssODN_RET_M918_I920silentC, or ssODN_RET_M918) were independently introduced into FB4-14 iPSC (GE2re, GE3re, and GE4re, respectively). The indel rate (generated by NHEJ after cleavage) in the target sequence (RET_MEN2B_Target) was then calculated by AmpliSeq analysis. Although the proportion of indels in the negative control cells (untransfected cells) was 0.7% (Supplementary Table X4, S5), the indel rates in GE2re, GE3re, and GE4re were 21.2%, 12%, and 11.5%, respectively (S2, S3, and S4). As was observed in the T7E1 assay and ICE analysis, these results indicate that selective cleavage at the target sequence by *As*Cas12a_RR with crRNA_RET-1m occurs frequently in HDR situations.

To determine the off-target effects of *As*Cas12a_RR with crRNA_RET-1m, we next performed AmpliSeq analysis using two predicted off-target candidates (RET_Off-target1 and RET_Off-target2).

For RET_Off-target1, the indel rate in the negative control was 0.3% (Supplementary Table X4, S10), whereas those in GE2re, GE3re, and GE4re were 0.5%, 0.4%, and 0.4%, respectively (S7, S8, and S9). For the RET_Off-target2 assay, the indel rate in the negative control was 0.9% (S15), whereas those in GE2re, GE3re, and GE4re were 0.9%, 1.0%, and 1.0%, respectively (S12, S13, and S14). These results suggested that the off-target effects of *As*Cas12a_RR with crRNA_RET-1m are only at a background level. In addition, similar results were obtained for the other two tools (*As*Cas12a_RR with crRNA_RET1+ and *As*Cas12a_RR with crRNA_COL7A1-1m) used in the HDR experiments (Supplementary Table X4).

In summary, these results strongly suggest that the three genome-editing tools used in this study (*As*Cas12a_RR with crRNA_RET-1m, *As*Cas12a_RR with crRNA-RET1+, and *As*Cas12a_RR with crRNA_COL7A1-1m) do not have any off-target effects in a HDR situation.

Additional Data 4. RFLP assay to assess HDR efficiency.

Results:

Several HDR/NHEJ modification compounds that can promote HDR have been reported[2-7]. To estimate whether these reagents could promote HDR in the One-SHOT method, the effects of these compounds on HDR efficiency were evaluated by PCR-RFLP analysis. Eight compounds (Brefeldin A, L-755,507, SCR7 pyrazine, Ku-0060648, NU7441, Nocodazole, VE822, and AZD7762) and two combinations (SCR7 + Ku-0060648 and VE822 + AZD7762) were examined (Supplementary Fig. X4); however, none of the compounds or combinations had a positive effect on HDR efficiency (N =3, $P > 0.05$). The results indicated that these low-molecular-weight compounds do not promote HDR in our system.

Additional Data 5. Karyotyping with chromosomal G-band analysis of the gene-edited clones.

Results and Discussion:

To confirm whether our genome editing tool causes chromosomal aberrations, karyotyping with chromosomal G-band analysis was performed (see Extended Materials and Methods). The parental cells (FB4-14, MEN2B-specific iPSC; B117-3, DEB-specific iPSC) exhibited normal chromosome structure prior genome editing (Supplementary Fig. X5a, f). Normal chromosome structure was also observed in all four FB4-14-derived gene-edited clones (Supplementary Fig. X5, b-e). However, only three of the four B117-3-derived gene-edited clones exhibited normal chromosome structure, with the fourth clone containing an additional fragment (Supplementary Fig. X5, g-j).

These results indicate that, in most cases, our genome editing protocol does not cause

chromosomal abnormalities. However, abnormalities do occur, albeit infrequently. An unexpected chromosomal truncation is induced by CRISPR-Cas9 system in some conditions[8], suggesting the abnormalities might be general phenomena in gene-editing. Therefore, if these cells are used clinically, they should be karyotyped prior to use to confirm a normal chromosome structure.

Additional Data 6. Analysis of plasmid integration.

Results:

The use of a plasmid vector to deliver gene editing tools results in the possibility that plasmid-derived sequences may be integrated into the genome of gene-edited cells during the editing process. To determine whether plasmid integration occurred using our method, a highly sensitive two-stage PCR assay was designed to detect the presence of plasmid-derived sequences in gene-edited cells (Supplementary Fig. X6a). First, to evaluate the sensitivity of the designed primer pairs, PCR was performed using a pY211-puro dilution series. Three of the plasmid-specific primer sets showed a high level of sensitivity, with products of the correct sizes amplified in reactions containing template at a concentration of 10 aM (nine molecules of DNA per reaction tube). However, none of the primer pairs reliably amplified the correct products at a template concentration of 1 aM (about one molecule per reaction tube). This may partly be attributed to the fact that when dispensing a solution containing a very low concentration of a molecule into a number of separate chambers, the distribution of that molecule in the chambers follows Poisson distribution[9], and chance of detection is only about 0.63. No amplification was observed at template concentrations below 0.1 aM (Supplementary Fig. X6b).

Next, to clarify whether pY211-puro-derived sequences were integrated into the gene-edited clones during editing, the three primer pairs were used to screen 25 different gene-edited clones for the presence of plasmid sequences. Primers designed to amplify the human β-actin gene were also included as a positive control for the presence of human DNA and as a reaction control, and amplification was observed from all 25 samples (Supplementary Fig. X6c, row beta-actin). To screen for the presence of sequences derived from pY211-puro, we then performed PCR assays using the three plasmid-specific primer pairs (Supplementary Fig. X6a). The results showed various levels of random integration depending on the sequence: none (puroR: 0/25), and low frequency (AsCas12a-2: 5/25, AsCas12a-8: 8/25) (Supplementary Fig. X6c). In addition, products of the correct sizes were amplified using all three primer pairs with 100 aM or 10 aM pY211-puro template DNA (positive control) (Supplementary Fig. X6c. P1, P2). These results indicate that the plasmid DNA is partially integrated into the gene-edited clones using our method with low frequency. Therefore, when a plasmid-based platform is used for genome editing, random plasmid integration should be examined in each genome-edited clone.

Additional Data 7. Fluorescence immunostaining for pluripotent gene markers in parental iPS cells.

Results:

To determine the subcellular localization of the four pluripotent gene markers (NANOG, OCT4/POU5F1, SSEA-4, and TRA-1-60) in parental iPS cell lines (FB4-14 and B117-3), fluorescence immunostaining with DAPI was performed. In both cell lines, transcription factors NANOG and OCT4/POU5F1 were localized in DAPI-positive cell nuclei. In contrast, the cell surface markers SSEA-4 and TRA-1-60 were broadly distributed (Supplementary Fig. X7).

Additional Data 8. Details of secondary screening-passed gene-edited clones generated by standard/scarless One-SHOT.

Results and Discussion:

To assess the results of the secondary screening of the standard One-SHOT clones, we aligned the Sanger sequencing data from each of the gene-edited clones (Supplementary Fig. 8a). Clones that were positive in the primary screening included those that had been repaired (11/17, Supplementary Fig. X8a, row 2). This suggests that SNMD-PCR-based screening for the SN marker is an efficient way of detecting the positive clones. In addition, there were two further classes of clones that were identified as positive in the primary screening: those that were repaired but contained a deletion (Supplementary Fig. X8a, row 3 and 4), and those that contained a simple deletion (Supplementary Fig. X8a, rows 5 and 6). It is reasonable that the repaired clones that contained a deletion were identified in the primary screening because they contain the SN marker (Supplementary Fig. X8a, Note). How the clones containing a simple deletion were identified is less clear, but is probably the result of miss-picking.

We next aligned the Sanger sequencing data for the gene-edited clones generated using the scarless One-SHOT method (Supplementary Fig. 8b). Repaired clones were included amongst those identified as negative in the primary screening (five clones, Supplementary Fig. X8b, row 2). This shows that negative screening by SNMD-PCR for the SN marker is useful for the detection of clones containing the intended gene edit. However, four additional classes of clones were identified as negative by the primary screening: group (1), identical to the original cell (seven clones, Supplementary Fig. X8b, row 1); group (2), clones containing deletions in both alleles (two clones, Supplementary Fig. X8b, row 3); group (3), clones containing deletions in the target allele containing the forward primer binding site for SNMD-PCR (six clones, Supplementary Fig. X8b, rows 4–9); and group (4), clones containing deletions in the target allele without the forward primer

binding site (Supplementary Fig. X8b, rows 10–13). Identification of clones from groups 2 and 4 as negative clones is reasonable because these clones do not have the forward primer binding site (Supplementary Fig. X8b, Note). The misidentification of clones from groups 1 and 3 as containing the intended gene edit might be caused by false-negatives in SNMD-PCR screening, which can be caused by low concentrations of template DNA.

Extended Materials and Methods

Design and construction of the *As*Cas12a_RR plasmid and crRNA. To generate an all-in-one vector for the *As*Cas12a_RR variant carrying a puromycin resistance gene, we started with the pY211 mammalian expression vector containing expression cassettes for *As*Cas12a_RR cDNA and the crRNA backbone[10] (a gift from Dr Feng Zhang; Addgene plasmid number 89352). For our study, we replaced the 3×HA tag[11] with a 3×HA tag, T2A peptide cDNA[12,13] and SGFP2 cDNA[14]. Briefly, 3×HA and T2A fragments were constructed by annealing ssODNs to each, while SGFP2 cDNA was amplified from pSGFP2-C1 (a gift from Dr Dorus Gadella; Addgene plasmid number 22881). pY211 was then cleaved using restriction enzymes *Bam*HI and *Eco*RI and all fragments were fused using a Clontech In-Fusion HD Cloning Kit (Takara Bio, Japan), generating plasmid pY211-T2G. cDNA corresponding to a puromycin resistance gene[15] was then PCR-amplified from vector pSIH-H1-Puro (a gift from Dr Frank Sinicrope; Addgene plasmid number 26597) and ligated into pY211-T2G at the *Spe*I and *Eco*RI sites. The final all-in-one vector was designated pY211-puro (Supplementary Fig. S6).

crRNAs were manually designed as previously described[10,16]. The crRNA guide sequence templates, which targeted *RET* exon 16 or *COL7A1* exon 78 (Supplementary Table S1), were cloned into pY211-puro digested with *Bbs*I, as described previously[10].

ssODN repair template design. The 99-nt ssODN templates used for repair/modification (PAGE-purified; Sigma-Aldrich, USA) included 5′-flanking and 3′-flanking 49-bp homologous genomic sequences centred on the target nucleotide (pathogenic SNV or the wild-type nucleotide), which contained the predicted CRISPR-Cas12a_RR cleavage site and/or silent mutations for use as single-nucleotide markers (Supplementary Table S1). Silent mutations were selected by consulting the Codon Usage Database (https://www.kazusa.or.jp/codon/).

Gene editing. To generate revertant and homozygous *RET*$^{\mathrm{Met918Thr/+}}$ iPSC lines, the FB4-14 cells were electroporated with pY211-puro-crRNA_RET-1m/ssODN and pY211-puro-crRNA_RET-1+/ssODN combinations, respectively (as described in Figs. 2, 3 and 4, Supplementary Fig. S5 and Supplementary Table S1). To generate the revertant COL7A1$^{\mathrm{G2138X/+}}$ iPSC lines, B117-3 cells were electroporated with a pY211-puro-crRNA_COL7A1-1m/ssODN combination (as described in Supplementary Fig. S4 and Supplementary Table S1). Electroporated cells were selected on puromycin-supplemented plates, followed by single-cell-derived clonal colony

formation, SNMD-PCR and sequence analysis, as described below. In total, five or six iPSC lines per genotype were isolated and characterised.

Generation of induced pluripotent stem cells (iPSCs) and quality checking. iPSCs were generated from human T-cells using a CytoTune-iPS 2.0 Sendai Reprogramming Kit (Thermo Fisher Scientific, USA), according to the manufacturer's protocol[17]. Briefly, human T-cells isolated from a peripheral blood sample were seeded at a density of 1.5×10^6 cells/well in a 6-well plate coated with anti-CD3 (eBioscience) 1 day prior to infection. Next, 3×10^5 cells were infected with recombinant Sendai virus (SeV) vectors carrying reprogramming factors at a multiplicity of infection of 10. After 2 days of culture, the infected cells were harvested and re-plated at a density of 2×10^4 cells per 100-mm dish onto mitomycin C (MMC)-treated mouse embryonic fibroblasts (MEFs), which acted as feeder cells. From 20–23 days post-infection, colonies were picked and re-cultured in human iPSC medium (detailed below). To remove the SeV, the iPSCs (passage 1) were cultured at 38°C for 3 days.

To confirm the quality of the newly-established iPSCs (FB4-14 and B117-3 cells), the expression of five pluripotency genes (*NANOG*, *REX1*, *SALL4, GDF3* and *DNMT3B*) and four Yamanaka factors (*OCT4 (also known as POU5F1)*, *SOX2*, *KLF4* and *C-MYC*) was examined by RT-PCR with a pre-designed primer set (Supplementary Table S1). Total RNA, which was purified using Sepasol Super G Reagent (Nacalai Tesque, Japan), was transcribed into cDNA using Superscript III (Invitrogen, USA) and random primers (Invitrogen). RT-PCR was performed using QuickTaq (TOYOBO, Japan), as previously described[18]. Primers used for amplification of *OCT4/POU5F1*, *SOX2*, *KLF4* and *C-MYC* were designed using Vector NTI (Thermo Fisher Scientific) to allow detection of the expression of the endogenous genes, but not the transgenes. To detect SeV DNA, nested RT-PCR was performed. The primer sequences are provided in Supplementary Table S1. The expression levels of the pluripotency markers (*NANOG*, *OCT4/POU5F1*, *SSEA4* and *TRA-1-60*) were also confirmed by immunofluorescence. The absence of mycoplasma contamination was confirmed using a MycoAlert Mycoplasma Detection Kit (Lonza, Switzerland).

iPSC culture. iPSCs were maintained on MMC-treated MEFs plated in cell culture plates coated with 0.1% gelatine and cultured in iPSC medium at 37ºC with 5% CO_2 (Soga et al. 2015). This medium consisted of DMEM/F12 (Sigma-Aldrich) supplemented with 20% KNOCKOUT serum replacement (Invitrogen), 2 mM L-glutamine (Thermo Fisher Scientific),

0.1 mM nonessential amino acids (Sigma-Aldrich), 0.1 mM 2-mercaptoethanol (Sigma-Aldrich), 0.5% penicillin and streptomycin (Nacalai Tesque, Japan) and 5 ng/ml basic fibroblast growth factor (Wako Chemicals, Japan). Prior to transfection, the iPSCs were transferred to Matrigel-GFR (Corning Inc., USA)-coated cell culture plates and cultured in MEF-conditioned iPSC medium containing 10 μM ROCK inhibitor (Wako Chemicals) for 2 days. Finally, the cells were grown in mTeSR1 medium (STEMCELL Technologies, Canada) in cell culture plates coated with Matrigel-GFR at 37°C with 5% CO_2.

Cell staining, immunocytochemistry and microscopy. Alkaline phosphatase staining[19] was performed using a Leukocyte Alkaline Phosphatase Kit (Sigma-Aldrich). Immunocytochemistry was performed as previously described[2]. In some experiments, nuclei were stained with 1 mg/ml DAPI (Nakarai, Japan). Stained cells were viewed under an all-in-one BZ-8100 microscope (Keyence, Japan) and images were acquired using the imaging software provided (Keyence). Adobe Photoshop was used to adjust the contrast and add scale bars to the images.

Antibodies. The following antibodies were used for immunocytochemistry: anti-Oct-3/4 (1:1000; sc-5279, Santa Cruz Biotechnology, USA), anti-TRA-1-60 (1:1000; MAB4360, Millipore), anti-SSEA-4 (1:1000; MAB4304, Millipore), anti-Nanog (1:1000; #4903, Cell Signaling Technology, USA), anti-mouse IgG (1:1000; AF488-labeled goat anti-mouse IgG, A11029, Molecular Probes, USA), and anti-rabbit IgG (1:1000; AF488-labeled donkey anti-rabbit IgG, A21206, Molecular Probes).

DNA extraction. Genomic DNA was extracted using a proteinase K-based protocol. Briefly, cell samples were resuspended in 10 μl of lysis buffer (DirectPCR Lysis Reagent Cell, Viagen Biotech, USA) and incubated at 55°C for 12 h. Proteinase K was inactivated by heat-treatment at 85°C for 45 min.

T7E1 assay. A T7E1 assay[20] for enzyme mismatch cleavage analysis was performed using an EnGen Mutation Detection Kit according to the manufacturer's instructions (New England Biolabs, USA), with minor modifications. Briefly, genomic DNA was extracted from gene-edited iPSCs (as described in Materials and Methods) and used as template to amplify 450–500-bp fragments from around the gene-edited locus using AmpliTaq Gold 360 Master Mix (Thermo Fisher Scientific), which were then column purified. The amplicons were annealed and digested

with T7 endonuclease I, and the digested DNA was separated on a premade polyacrylamide gel (SuperSep Ace 5-20% 13 well; FUJI FILM, Japan). Gene Ladder Wide I (Nippon Gene, Japan) was used as DNA molecular size marker. After SYBR Gold staining, gel images were captured by digital camera (CANON, Japan). Densitometry was performed using ImageJ32[21]. Indel quantification was based on the relative intensities of bands using the formula: $1-(\sqrt{1-(b+c)/(a+b+c)})$, where a is the intensity of the undigested PCR product and b and c are the intensities of the cleavage products[22].

PCR-RFLP assay. For the PCR-RFLP assay[23], 450–500-bp fragments from around the gene-edited locus were PCR-amplified from the genomic DNA of the gene-edited iPSCs using AmpliTaq Gold 360 Master Mix (Thermo Fisher Scientific). The amplicons were digested with restriction enzymes (*Alw*I, *Taqα*I or *Afl*III) and then separated on a premade polyacrylamide gel (SuperSep Ace 5-20%, 13 well, FUJI FILM). Gene Ladder Wide I (Nippon Gene, Japan) was used as DNA molecular size marker. After staining, imaging and densitometry analysis, cleavage activity was quantified from the relative intensities of the bands using the formula: $1-(\sqrt{1-(b+c)/(a+b+c)})$, where a is the intensity of the undigested PCR product and b and c are the intensities of the cleavage products[24].

Equipment and Settings. For image processing and densitometry analysis, ImageJ32 on MacOS 10.6.8 was used. Pre-processing of digital images was performed as the following: images were rotated as the following command line (Image> Transform> Rotate), being converted to grayscale (16-bit) as the following command line (Image> Type> 16 bit). Next, Background Subtraction was performed as the following command line (Background… : Process> Subtract Background) with the following setting (Rolling ball radius = 50 pixels)). Densitometry analysis was performed as the following command line (Analyze> Gels> Gel Analyzer Options…) with the following setting (Vertical scale factor: 1.0, Horizontal scale factor: 1.0, Uncalibrated OD: ON, Label with percentage: ON, Inverted peaks: Off).

Off-target analysis. Gene-edited, disease-specific iPSCs were tested for off-targeting events as predicted for each guide RNA using CHOPCHOP version 2, a web-based CRISPR design tool designed by Eivind Valen's laboratory[25] (http://chopchop.cbu.uib.no). All off-target sites were profiles using CHOPCHOP ver. 2 (two targets each in GE1–GE4, seven targets in GE5). The genomic DNA region surrounding each off-target site was PCR-amplified (Supplementary

Table S1), sequenced and compared against the latest RefSeq[26,27] human genome assembly (Dec. 2013, GRCh38/hg38) using the UCSC Genome Browser[28,29] (http://genome.ucsc.edu/).

Clonality analysis. To investigate the clonality of the gene-edited clones, the sequences of the unintended gene-edited clones that passed the first round of screening were analysed. The unintended gene-edited clones contained some indels caused by NHEJ after gene-editing. The composition of the unintended gene-edited clones indirectly indicates the clonality of the intended edited clones. The sequencing reads from the unintended gene-edited clones were manually separated into single reads for the wild-type/mutant alleles and indel-containing alleles. Reads containing indels were analysed for their distribution by manually aligning them against the reference sequence (Supplementary Fig. S3).

One-SHOT experimental workflow. *DesignandtestingofCRISPRRNAs:* the genomic sequence around the target site in the intended cell was first verified by direct sequencing (Supplementary Fig. S1a-1). Templates for the guide crRNA sequences were then designed (Supplementary Fig. S1a-2). The dsODN templates for crRNAs were inserted into the *As*Cas12a_RR all-in-one expression vector (Supplementary Fig. S1a-3, Supplementary Fig. S6) and *As*Cas12a_RR cleavage activity was tested by T7E1 assay using wild-type iPSCs (Supplementary Fig. S1a-3).

HDR: a ssODN for the repair/modification template was designed based on the determined sequence (Supplementary Fig. S1a-4). Prior to genome editing, the intended iPSCs were acclimated to feeder-free culturing conditions in chemically-defined mTeSR1 medium (Supplementary Fig. S1a-5). The genome editing tools (all-in-one vector expressing *As*Cas12a_RR, crRNA and the puromycin resistance gene, along with the ssODN template) were introduced into the intended iPSCs by electroporation (Supplementary Fig. S1a-6). To concentrate the electroporated cells, the cells were treated with puromycin-containing medium for 48 h then cultured in mTeSR1 plus CloneR recovery medium for 1–2 days (Supplementary Fig. S1a-7). After puromycin selection, the cells were dissociated into single cells, sparsely plated at a density of 500–1,000 cells per 100-mm plate (Supplementary Fig. 1a-8) and cultured for 7–8 days until single-cell-derived colonies formed on master plates. Plates with a square grid were used to record the location of the colonies on the plates for subsequent experiments (Supplementary Fig. 1b).

Screening for intended clones: To extract DNA from each colony, the central portion

of the colony was picked using a clean 10-μl pipette tip[30] (Fig. 1a-9). In the first round of screening, the candidate clones were selected by positive screening via SNMD-PCR (Fig. 1a-10). Clones that passed the first round of screening were picked from the master plates and maintained separately. In the second round of screening, the sequence around the target site of each clone that passed the first round was determined by direct sequencing (Fig. 1a-10). Finally, the identity and the clonality of the expanded clones was reconfirmed by direct sequencing (Fig. 1a-11).

Additional Data 1 & 3.

AmpliSeq analysis. Genomic DNA was extracted from ~2×10^3 to 2×10^4 cells from each cell line using a DNeasy Blood & Tissue kit (Qiagen, Germany). In the primary PCR, fragments containing the target sequences were amplified as per the manufacturer's instructions using Tks Gflex DNA polymerase (Takara Bio, Japan) and primary extended primer pairs (Supplementary Table X1). These amplicon-specific primers each included a 5′ sequence tag (forward primer: TCGTCGGCAGCGTCAGATGTGTATAAGAGACAG; reverse primer: GTCTCGTGGGCTCGGAGATGTGTATAAGAGACAG) used to anneal Nextera XT index primers[31] (Illumina, USA). Thermal cycler parameters were as follows: 35 cycles of 98°C for 10 seconds, 60°C for 15 seconds, and 68°C for 30 seconds. The sizes of expected products ranged from 362–414 bp. The resulting amplicons were purified using Agencourt AMPure XP magnetic beads (Beckman Coulter, USA) and the FastGene MagnaStand 96 system (Nippon Genetics, Japan).

In the second PCR[31], Illumina dual index and sequencing adaptors were attached to the PCR products using a Nextera XT Index Kit (Illumina). The thermal cycler parameters for the second PCR were as follows: 95°C for 3 minutes, 8 cycles of 95°C for 30 seconds, 55°C for 30 seconds, and 72°C for 30 seconds, and a final extension at 72°C for 1 minute. The resulting amplicons were purified using magnetic beads, and the concentration of the purified products was determined by real-time quantitative PCR using the LightCycler Nano system (Roche, Germany) and a GenNext NGS Library Quantification Kit (TOYOBO, Japan). The purified products were then mixed at equimolar concentrations to generate a 4-nM library pool. The amplicon libraries were subjected to 2 × 250 bp paired-end sequencing using the MiSeq system (Illumina) and a MiSeq v2 Reagent Kit[31] (500 cycles; Illumina).

Bioinformatics analysis. Bioinfomatics analysis of the AmpliSeq data was performed by Rhelixa Inc. (Tokyo, Japan, https://www.rhelixa.com/). The resulting FASTQ sequencing data were then analysed as follows. First, the quality of the sequence reads was checked using FastQC[32] (ver.

0.11.7), and low-quality sequence reads were removed using Trimmomatic[33] (ver. 0.36). Finally, the quality of the trimmed sequence reads was confirmed using FastQC (ver. 0.11.7). The remaining sequence reads were mapped against the reference genome (hg38) using Bowtie2[34] (ver. 2.3.4.2). After extracting unique reads, the mapping domains and read counts were calculated using BEDTools[35] (ver. 2.27.1). Based on the overlaps between the sequenced paired-end reads, contigs were generated by merging the reads using FLASH[36] (min. overlap = 30; max. mismatch density = 0). Low-quality sequences were then trimmed using Trimmomatic and eliminated from the analysis based on the following criteria: (a) shorter than 300 bp, (b) average quality score < 30 in a sliding window. Reads containing indels and SNPs were then counted and outputted as csv files using CRIS.py (ver. 2)[37].

Based on the CRIS.py analysis, the ratios of each of the top 10 reads were calculated and the data converted into FASTA format. An alignment diagram was generated by aligning the converted sequence reads and presenting them together with each read ratio using MEGA X[38]. For the clonal purity analysis, sequence contamination from the parental cell line was determined and shown at the bottom of the diagram together with the read ratio.

To assess the clonal purity of the gene-edited cells, read counts and frequency calculations for each sequence were performed using CRIS.py. The inputted reference and flanking sequences are shown in Supplemental Table X2. The significance of the parental allele ratio determined by CRIS.py analysis was then examined using GraphPad Prism 7 (GraphPad Software Inc., USA). To assess on-/off-target effects in gene-edited cells, the ratios of indels determined by CRIS.py analysis were manually corrected and summarised in Supplementary Table X4.

Additional Data 2.

Inference of CRISPR edits (ICE) analysis. To examine the SSN activity of the four genome editing tools at the wild-type RET_M918 site, 10 μg of each of the genome editing constructs pY211-puro-crRNA_RET-1+, pY211-puro-crRNA_RET-1m, pY211-puro-crRNA_RET-2+, or pY211-puro-crRNA_RET-2m were electroporated into aliquots of normal iPS cells (409B2) (1×10^6 cells per aliquot) (Group A). To examine the SSN activity of the four genome-editing tools at the mutant RET_M918T site, 1×10^6 MEN2B-homo iPS cells (GE1-9) were electroporated with 10 μg of the genome editing constructs pY211-puro-crRNA_RET-1+, pY211-puro-crRNA_RET-1m, pY211-puro-crRNA_RET-2+, or pY211-puro-crRNA_RET-2m (Group C). To examine the SSN activity of the six genome-editing tools at the wild-type COL7A1_DEB_exon78 site, 1×10^6 normal iPS cells (409B2) were electroporated with 10 μg of the genome editing constructs pY211-puro-crRNA_COL7A1-1+,

pY211-puro-crRNA_COL7A1-1m, pY211-puro-crRNA_COL7A1-2+, pY211-puro-crRNA_COL7A1-2m, pY211-puro-crRNA_COL7A1-3+, or pY211-puro-crRNA_COL7A1-3m (Group B). As a negative control, non-electroporated, non-puromycin-treated cells were cultured simultaneously in each experiment.

At 16 hours post-electroporation, all cell cultures were treated with puromycin (0.5 µg/mL in mTeSR1 + CloneR) for 48 hours, followed by 24 hours in recovery culture (mTeSR1 + CloneR) before being collected. Genomic DNA was extracted from the cell pellets using a DNeasy Blood & Tissue kit (Qiagen).

Primer pairs designed for Sanger sequencing (Supplementary Table 1) were also used to amplify the RET_target sequence from genomic DNA extracted from cells from groups A and C and the COL7A1_DEB_target sequence from genomic DNA extracted from cells from Group B. Sanger sequencing was then performed using an ABI Prism 3130×1 DNA sequencer and a BigDye Terminator v3.1 Cycle Sequencing Kit (Thermo Fisher Scientific/Applied Biosystems, USA) according to the manufacturer's instructions.

ICE analysis was then performed by sequential processing of abi files using the dedicated ICE website (https://ice.synthego.com/). The correlation of the three elements (guide sequence, control file, experiment file) used in this analysis is shown in Supplementary Table X3. The total indel ratio determined by ICE analysis was statistically analysed using GraphPad Prism 7 (GraphPad Software Inc.).

Additional Data 4.

Assay of HDR/NHEJ modification compounds on HDR efficiency. pY211-puro with crRNA_RET-1m (10 µg) and ssODN_RET_M918_I913_silentC repair template (15 µg) were electroporated into 1 × 10^6 FB4-14 cells (MEN2B-specific iPSC) (for detailed conditions, see Extended Materials and Methods), which were then seeded into a 24-well plate at a concentration of 1.67 × 10^5 cells/well in mTeSR1 + CloneR basal medium. At 4 h post-electroporation, the cell culture medium was replaced with basal medium containing HDR/NHEJ modifiers dissolved in DMSO (0.1 µM brefeldin A (Cayman Chemical, USA), 1 µM L-755,507 (Cayman Chemical), 1 µM SCR7 pyrazine (TOCRIS, USA), 1 µM Ku-0060648 (Cayman Chemical), 1 µM NU7441 (TOCRIS), 0.1 µM Nocodazole (AdipoGen, USA), 1 µM VE822 (Axon Medchem, The Netherlands), 0.3 µM AZD7762 (TOCRIS), SCR7 + Ku-0060648 (1 µM each), or VE822 + AZD7762 (1 µM and 0.3 µM, respectively)) and cells were cultured for a further 44 hours. After washing with mTeSR1, basal medium containing 0.5 µg/mL puromycin was added to each well and plate were incubated for 16 hours, followed by a recovery period of 24 hours in unsupplemented basal medium.

For the PCR-RFLP assay, genomic DNA was extracted from cell samples using a DNeasy Blood & Tissue kit (Qiagen, Germany). Using the RET_target-specific primer pair designed for the T7E1 assay, fragments containing the RET_M918 site were amplified using an AmpliTaq Gold 360 Master Mix (Thermo Fisher Scientific, USA). The PCR products were purified using a MinElute PCR Purification Kit (Qiagen, Germany) and a 200-ng aliquot of each purified product was digested with AlwI for 1 hour. The digests were then subjected to sodium dodecyl sulphate polyacrylamide gel electrophoresis, stained, and photographed (for details, see Extended Materials and Methods). The band intensities on the gel were quantified using ImageJ32. Statistical analysis and graph construction were performed using GraphPad Prism 7 (GraphPad Software Inc., USA).

Additional Data 5.

Karyotyping. Karyotyping with chromosomal G-band analysis of the gene-edited clones was performed by NIHON GENE RESEARCH LABORATORIES Inc. (Sendai, Miyagi, Japan, http://www.ngrl-japan.com).

Additional Data 6.

Analysis of plasmid integration. We designed three primer pairs targeting functional regions of the pY211-puro vector (5′ and 3′ regions of *As*Cas12a_RR cDNA, cDNA corresponding to the puromycin resistance gene, Supplementary Fig. X6a), along with a human β-actin-specific primer pair (positive control), using Vector NTI (Thermo Fisher Scientific, USA). The sizes of the resulting amplicons were in the range of 350–478 bp (Supplementary Fig. X6). To determine the limits of detection of each of the primer pairs, a 10-fold dilution series (100 fM to 0.01 aM) was prepared from a 100-pM pY211-puro stock solution. Using this dilution series as a template, the pY211-puro-specific primer pairs were evaluated by PCR in 15-µL reaction mixes containing KAPATaq Extra HS ReadyMix + dye (2×) (Roche/KAPABIOSYSTEMS, USA), 1.5 µL of template DNA, and 0.75 µL of each primer. Using this method, 1 aM of DNA corresponds to approximately one molecule per reaction tube. A highly sensitive two-stage PCR program was then used to test primer sensitivity. For program R54A60, the first step was as follows: 94°C for 2 minutes, followed by 23 cycles of a ramp-down protocol consisting of 94°C for 20 seconds, 65°C for 15 seconds, and 68°C for 15 seconds (ramp rate: −0.5°C per cycle). The second step was a standard PCR assay consisting of 14 cycles of 94°C for 15 seconds, 60°C for 15 seconds, and 72°C for 15 seconds, with a final extension at 72°C for 1 minute. For program R54A54, the first step consisted of 94°C for 2 minutes, followed by a 23-cycle ramp-down protocol

of 94°C for 20 seconds, 65°C for 15 seconds, and 68°C for 15 seconds (ramp rate: −0.5°C per cycle), while the standard PCR step consisted of 14 cycles at 94°C for 15 seconds, 54°C for 15 seconds, and 72°C for 15 seconds, with final extension at 72°C for 1 minute, was conducted. R60A54 was used to amplify the amplicon of puroR. R54A54 was used to amplify amplicons of AsCas12a-1, and AsCas12a-8, and β-Actin.

To assess plasmid integration, 20 ng of genomic DNA extracted from the gene-edited clones was amplified using the four-stage PCR protocol described above. β-actin was used as a positive control for the detection of human genomic DNA. The primer pairs used in this assay are shown in Supplementary Table X5.

Extended Figure Legends

Figure 2. Single-nucleotide substitution of the *RET* wild-type sequence in MEN2B induced pluripotent stem cells (iPSCs). (**a**) Human *RET* locus containing the MEN2B mutation (*RET*: c.2753T>C (p.Met918Thr)), an autosomal-dominant heterozygous mutation in exon 16, and *As*Cas12a_RR CRISPR RNAs (crRNAs) used to repair the mutation. Top: exon 16 of the *RET* locus on chromosome 10q11. Underlining, the corresponding sequences in the middle and bottom rows (intron, grey; exon, pale green). Middle: the wild-type allele sequence. Blue letters, the wild-type nucleotide at Met918 (underlined). Bottom: the mutant allele sequence. Red letters, the single missense mutation caused by a T>C substitution (c.2753T>C, blue to red), producing a Met918Thr substitution (underlined). Coloured lines, the guide sequence template for the crRNAs (crRNA_RET-1+, light blue; crRNA_RET-1m, light green; crRNA_RET-2+, orange; and crRNA_RET-2m, pale orange). Pink line, *As*Cas12a_RR protospacer-adjacent motif (PAM). Coloured dashed lines, the cleavage sites generated by *As*Cas12a_RR with the corresponding crRNAs. (**b**) T7E1 assay using human wild-type iPSCs (409B2) electroporated with *As*Cas12a_RR and the different crRNAs (crRNA_RET-1+, crRNA_RET-1m, crRNA_RET-2+, or crRNA_RET-2m) targeting exon 16. Left: the cropped gel image of T7E1 assay. Arrowheads, cleaved bands. M, marker; NC, negative control. The full-length gel is presented in Supplementary Figure S7. In addition, to give priority to perform quantitative comparison on the same gel, electrophoresis of DNA size marker was performed on independent gel. Right: statistical analysis of the cleavage activity and specificity of *As*Cas12a_RR with the crRNAs after selection with different concentrations of puromycin (0, 0.5 or 3–0 µg/ml. 3–0: 3 µg/ml for the first 24 h and 0 µg/ml for the last 24 h). Bars, S.E.M. (n = 3). One-way ANOVA with Tukey's multiple comparison test: *Comparison of crRNA_RET-1+ with negative control (NC) ($P < 0.0001$); **Comparison of crRNA_RET-2+ with NC. $P < 0.05$; ***Comparison between crRNA_RET-1+ and crRNA_RET-2+ ($P < 0.0001$); ****The effect of puromycin treatment ($P < 0.0001$); ns, not significant. (**c**) Homology-directed repair-mediated editing to generate artificial homozygous MEN2B alleles using *As*Cas12a_RR and crRNA_RET-1+, which selectively targets *RET*_Met918 in the wild-type allele. Light blue line, the targeting location of crRNA_RET-1+; pink line, *As*Cas12a_RR PAM. Red, blue and light green letters denote the MEN2B pathogenic single-nucleotide variant (SNV), the wild-type original nucleotide and the single-nucleotide marker used for single-nucleotide mismatch detection polymerase chain reaction (SNMD-PCR) screening, respectively. Pale blue and green columns, the positions of the

pathogenic SNV and the single-nucleotide marker. The arrow indicates the forward primer position for the SNMD-PCR. (**d**) SNMD-PCR analysis of the first round of screening. The cropped gel image is shown here. The arrowhead shows positive PCR amplicon (202-bp product). The full-length gel is presented in Supplementary Figure S8. (**e**) Sequencing of the original and modified MEN2B iPSCs (FB4-14). Top: wild-type *RET* exon 16 sequence containing a T>C substitution in the MEN2B mutant allele that generates a heterozygous Met>Thr substitution. Bottom: the modified *RET* sequence. The T>C substitution resulting in a homozygous Met>Thr substitution. Red and blue arrows indicate the positions of the pathogenic SNV and the single-nucleotide marker, respectively. Underlining indicates the codons affected by the editing.

Supplementary Figure Legends

Supplementary Figure S1. (**a**) Experimental flow of the One-SHOT protocol. A detailed explanation of each step is provided in the Extended Materials and Methods above. This illustration was drawn with reference to Kwart et al.[39] (**b**) Colony mapping. For the single-cell-derived colonies cultured in the master plate (left, 100-mm plate with grid), the position, size, morphology and identification number are recorded on the map (right). Based on this map, the appropriate colonies are selected and sampled. Following single-nucleotide mismatch detection polymerase chain reaction screening and sequencing, the passed clones are identified based on their map positions.

Supplementary Figure S2. Establishment and quality check of MEN2B-specific induced pluripotent stem cells (iPSCs). (**a**) T-cells were isolated from a patient with MEN2B and then infected with a recombinant Sendai viral vector carrying Yamanaka reprogramming factors. iPSCs were then established on mitomycin C-treated mouse embryonic fibroblasts (feeder cells). The Sendai virus was not detected in the established cell line. FB4-14 cells were classified as true iPSCs based on the following: continuous expression (determined by reverse-transcription polymerase chain reaction (RT-PCR)) of four Yamanaka factors (**a**); expression of typical pluripotency markers (determined by RT-PCR and immunofluorescence) (**a** and **b**); and round-shaped colonies composed of high-density epithelial cells (**b**). (**b**) All established iPSCs were alkaline phosphatase-positive. In (**a**), images cut from different parts of the same gel are displayed as a group. The original images are shown in Supplementary Figure X9. Abbreviations. PC, positive control. DW, double distilled water.

Supplementary Figure S3. Composition of clones that passed the first-round screening. Sanger sequencing around the target sequence was performed to determine the composition of each population of the clones that passed the first round of screening. The clones were then classified into five groups: 1) Intended, repaired/modified clones (blue); 2) non-edited, original clones (red); 3) unintended edited clones, non-duplicated (green); 4) unintended edited clones, duplicated (purple); 5) unread clones (grey). The percentage of intended clones was ~60% in three standard one-SHOT experiments (GE1, GE2 and GE3) and 10–30% in the scarless One-SHOT experiments (GE4 and GE5). For scarless One-SHOT, non-edited original clones were mixed in the populations (red). In experiments GE3 and GE5, ~23% of clones were unintended, duplicated

clones (purple).

Additional Explanations of Classification.

(blue) *Intended, repaired, or modified clones* refers to an edited clone in which the target was precisely cleaved by a genome editing tool, and where subsequent HDR using ssODN template resulted in the intended genome edit (repair or modification). Specifically, it refers to row 2 in Supplementary Fig. X8a and X8b.

(red) An *original unedited clone* is a clone that has the same alleles as the parental strain. Specifically, it refers to row 1 in Supplementary Figure X8a and X8b.

(green/purple) *Unintended edited clone* refers to an edited clone (= clone processed using a genome editing tool) in which a unintended indel is generated near the target as a result of target cleavage activity by the genome editing tool. Specifically, it refers to row 3-6 in Supplementary Figure X8a and row 3-13 in Supplementary Figure X8b.

(green) Refers to the clone that is not duplicated in the unintended edited clone. In other words, a clone whose indel is unique and has no other clones with the same sequence. This specifically refers to row 3, 4, and 6 in Supplementary Figure X8a and row 4-13 in Supplementary Figure X8b. Row5 in Supplementary Figure X8a was exceptionally classified as not-duplicated clone, because it was presumed that the deletion was located at the cleavage site by Cas12a-crRNA_RET-1m and was originally abundant.

(purple) Refers to the duplicate clone in the unintended edited clones (i.e., there is another clone with the same sequence as the indel). Specifically, it refers to row 3 in Supplementary Figure X8b.

(grey) An *unreadable clone* is a clone whose sequence was unreadable by Sanger Sequencing as a result of multiple overlapping wave traces.

Supplementary Figure S4. Mono-allele-specific single-nucleotide substitution at other pathogenic single-nucleotide variants (SNVs) in DEB-specific induced pluripotent stem cells (iPSCs) (B117-3) by scarless One-SHOT. (**a**) Quality check of DEB-specific iPSCs (B117-3) by RT-PCR and Fluorescent immunostaining. On the right side of this figure, images cut from different parts of the same gel are displayed as a group. The original images of the RT-PCR are shown in Supplementary Figure X9. (**b**) Design of *As*Cas12a_RR CRISPR RNAs (crRNAs) for the COL7A1 exon 78 target site in B117-3 cells (*COL7A1*$^{\text{p.Gly2138Ter/+; c.3591del13ins GG/+}}$) and the single-stranded oligodeoxynucleotide (ssODN) repair template. Top: location of the target site in the *COL7A1* locus. Middle and bottom: the genomic sequence around the target sites. The red and

blue letters indicate the pathogenic SNV and the wild-type nucleotide for DEB, respectively. Underlining indicates the codons affected by the SNV. The pink lines indicate the *As*Cas12a_RR protospacer-adjacent motifs. The blue lines indicate the crRNA guide sequences for the wild-type target sequence. The light blue lines indicate the crRNA guide sequences for the mutant target sequence. The grey lines show the fourth candidate, which was not used because the 20th nucleotide of this guide sequence exhibited base-mismatch tolerance. The bottom line shows the ssODN repair template for single-nucleotide substitutions. (**c**) T7E1 assay of *As*Cas12a_RR crRNAs for *COL7A1*$^{p.Gly2138Ter}$ in wild-type iPSCs (409B2). Among the three crRNA candidates, crRNA_COL7A1-1+ had the strongest cleavage activity for the wild-type target sequence. Left: the red arrowheads indicate the cleaved fragments as determined by T7E1. Right: statistical analysis of the cleavage activity and specificity. The black, blue and light blue dots indicate the negative control (NC), crRNAs for the wild-type target and the crRNAs for the mutant target, respectively. Bars represent mean and S.E.M. from experiments performed in biological triplicate. Data were analysed by one-way ANOVA with Tukey's multiple comparison test. ns, not significant. (**d**) SNMD-PCR screening. The arrowheads denote the intended clones from this screening (224 bp). (**e**) Typical ABI sequence data obtained before and after the gene-editing in B117-3 cells. The red arrows indicate the position of the SNV. Underlining indicates the codons affected by the editing.

Supplementary Figure S5. Marker locations in the human *RET* mutant allele. (**a**) Alternative single-nucleotide marker sites for single-nucleotide mismatch detection polymerase chain reaction (SNMD-PCR)-based screening to identify gene-edited clones. Candidate sites were designed for homology-directed repair-mediated editing. The green line shows the target of crRNA_RET-1m, while the pink line represents the *As*Cas12a_RR protospacer-adjacent motif. Red, blue and light green letters denote the MEN2B pathogenic single-nucleotide variant (SNV), the wild-type original nucleotides and the single-nucleotide markers for SNMD-PCR screening, respectively. Pale blue and green columns show the positions of the pathogenic single-nucleotide variant and the single-nucleotide markers used for screening. Underlining denotes the restriction enzyme cleavage sites. The arrow indicates the position of the forward SNMD-PCR primer. (**b**) PCR-restriction fragment length polymorphism (RFLP) assay using human MEN2B induced pluripotent stem cells (iPSCs) (FB4-14). Top: PCR-RFLP assay. Blue arrowhead shows the original amplicons (474 bp). Red arrowheads show the cleaved bands after *Alw*I, *Taq*αI and *Afl*III digestion. M, marker; NC, negative control (mock transfected). Bottom: quantitative PCR-RFLP

assay analysis. Bars represent mean and S.E.M. from experiments performed in biological triplicate. Data were analysed by one-way ANOVA with Sidak's multiple comparisons test. ns, not significant. (**c**) First round of SNMD-PCR screening. Arrowheads indicate positive bands from the SNMD-PCR screening (183 bp). (**d**) Sequencing chromatograms from the original and repaired MEN2B iPSCs. Top: the original *RET* sequence with a T-to-C substitution in the MEN2B mutant allele. Bottom: the repaired *RET* sequence. Red and blue arrows indicate positions of the pathogenic SNV and the single-nucleotide marker, respectively. The underlining indicates the codons affected by the editing.

Supplementary Figure S6. pY211-puro vector map. The pY211-puro vector is derived from pY211, an all-in-one vector carrying expression cassettes for the *As*Cas12a/Cpf1_RR variant and the CRISPR RNA. In the pY211-puro vector, the HA×3 tag has been replaced with a fragment containing HA×3, T2A and the puromycin resistance gene.

Supplementary Figure S7. The full-length images of Figure 2b. Top: the main portion. Bottom: the marker.

Supplementary Figure S8. The full-length gel image of Figure 2d.

Supplementary Figure S9. The full-length gel image of Figure 3a.

Supplementary Figure S10. The full-length gel image of Figure 3c.

Supplementary Figure S11. The full-length gel image of Figure 4c.

Supplementary Figures Legends for Additional Data

Supplementary Figure X1. Workflow and statistical analyses used to measure the clonal purity of single-cell-derived gene-edited clones. During clone picking, parental cells containing heterologous sequences in the target region may contaminate gene-edited cells containing homologous sequences. These contaminating heterologous sequences can easily be detected by AmpliSeq analysis. (**a**) Workflow of AmpliSeq analysis. AmpliSeq analysis proceeded as follows: i) deep sequencing of target reads using the MiSeq system; ii) pre-processing of FASTQ files (quality control, trimming, and mapping); iii) generation of contigs and extraction of high-quality reads; iv) enumeration of reads containing indels and SNPs; v) calculation of the ratios of the top 10 reads and generation of an alignment diagram. (**b**) Example of CRIS.py analysis results. The core sequence (PAM + crRNA guide sequence template) and its flanking sequences are shown. Top, reference sequence. Middle, top 10 read sequences with the read ratios. Bottom, read sequence contamination from parental cells with the read ratio. Arrowhead, substitution. Red open box, insertion. Hyphen, deletion. Black open box, core sequence. Yellow open box, unique nucleotide in the parental sequence. White open box, single-nucleotide marker. *, Reference sequence. **, Identical to reference sequence. ***, Repair template-derived sequence. ****, Most frequent error = base-line control. *****, Contaminated sequence derived from parental cells. The criteria used to determine whether a certain substitution/deletion/insertion was an error were as follows: 1) whether the target mutant (or normal) nucleotide is repaired (or modified), and 2) the presence or absence of the single-nucleotide marker is not important. However, if substitutions or indels occurred unintentionally at a low frequency, it was determined that the errors occurred during the sequencing and downstream data processing. (**c**) Statistical analyses of clonal purity based on the ratio of the allele derived from the parental cell line in each genome-editing experiment. The rate of parental contamination was determined from the data using CRIS.py analysis. Pairwise analysis was then used to compare the determined rate to that of the most frequent error as a base-line control. Bars represent mean and S.E.M. from experiments (N = 5). Data were analysed by one-way ANOVA with Sidak's multiple comparison test. ns, not significant. Abbreviations: GE, genome editing experiment; BLC, base-line control. For more information, see Additional Data 1.

Supplementary Figure X2. Results of Inference of CRISPR edits (ICE) analysis. (**a**) SSN activity of *As*Cas12a_RR with four crRNAs (RET-1+, RET-1m, RET-2+, or RET-2m) at the RET_M918 site (WT) in normal iPSC (409B2). (**b**) SSN activity of *As*Cas12a_RR with the four

crRNAs at the RET_M918T site (mutant) in MEN2B-homo iPSC (GE1-9). (**c**) SSN activity of *As*Cas12a_RR with six crRNAs (COL7A1-1+, COL7A1-1m, COL7A1-2+, COL7A1-2m, COL7A1-3+, or COL7A1-3m) at the COL7A1_DEB_exon78 site (WT) in normal iPSC (409B2). Horizontal axis, crRNAs. The vertical axis shows the ratios of the total number of indels generated by the SSN activity of each genome-editing tool at the corresponding target sequence. Bars represent mean and S.E.M. from experiments performed in quadruplicate (two independent cell pools and two independent Sanger sequencing analyses). Data were analysed by one-way ANOVA with Tukey's multiple comparison test. ns, not significant. For more information, see Additional Data 2.

Supplementary Figure X3. Detailed examination of on-target cleavage by AsCas12a with templates in HDR situations. Each section shows the central 60 nucleotides of the reference sequence at the top, followed by the top 10 sequence reads obtained by AmpliSeq along with the percentages of each of the variations. (**a**) GE1re, for modification (editing toolset: pY211-puro-crRNA_RET-1+ and ssODN_RET_M918T_I913silentC). (**b**) GE2re, for repair (editing toolset: pY211-puro-crRNA_RET-1m and ssODN_RET_M918_I913silentC). (**c**) GE3re, for repair (editing toolset: pY211-puro-crRNA_RET-1m and ssODN_RET_M918_I920silentC). (**d**) GE4re, for scarless repair (editing toolset: pY211-puro-crRNA_RET-1m and ssODN_RET_M918). (**e**) GE5re, for scarless repair (editing toolset: pY211-puro-crRNA_COL7A1-1m and ssODN_COL7A1_G2138). In the reference sequence, the PAM are shown in red, while the guide-sequence templates for crRNAs are shown in cyan. In the top 10 sequence reads, wild-type nucleotides, mutant nucleotides, and single nucleotide markers are shown in blue, red, and light green, respectively. Abbreviations: Ref, reference sequence; Ref*, reference sequence with SNV (mutant nucleotide); WT, wild-type allele; Mut, mutant allele; Mod, modified sequence; Rep, repaired sequence; Comp, complementary strand of the reference sequence. Hyphen, deletion. A hyphen with arrow indicates a deletion which has an insertion. Arrowheads, predicted cleavage pattern by *As*Cas12a with the crRNAs. For more information, see Additional Data 3-1.

Supplementary Figure X4. Effects of HDR/NHEJ modification compounds on HDR efficiency. (**a**) Cropped image of a gel showing the products of the PCR-RFLP assay. Lane 1, negative control (basal medium); lane 2, DMSO (0.08%); lane 3, Brefeldin A (0.1 μM*); lane 4, L-755,507 (1 μM); lane 5, SCR7 pyrazine (1 μM); lane 6, KU60648 (1 μM); lane 7, NU7441 (1 μM); lane 8, SCR7 + KU60648 (1 μM each); lane 9, Nocodazole (0.1 μM*); lane 10, VE822 (1 μM); lane 11, AZD7762 (0.3 μM*); lane 12, VE822 + AZD7762 (1 μM and 0.3 μM, respectively). Arrow, original amplicons;

arrowheads, cleaved fragments. *In these experiments, the doses of the reagents were reduced because preliminary experiments showed that the original doses were toxic to the iPS cells. (**b**) Statistical analysis of the PCR-RFLP data. The horizontal axis indicates the administered compound. The vertical axis indicates cleavage activity (%). Bars represent mean and S.E.M. from experiments performed in biological triplicate. Data were analysed by one-way ANOVA with Tukey's multiple comparison test. ns, not significant. *, $P < 0.001$. For more information, see Additional Data 4.

Supplementary Figure X5. Karyotyping with chromosomal G-band analysis of the gene-edited clones. Each panel shows a typical result of G-band analysis with a summary shown below. (**a**) FB4-14, wild-type MEN2B-specific iPSC. (**b**) GE2-33, FB4-14 revertant with SN marker. (**c**) GE2-46, FB4-14 revertant with SN marker. (**d**) GE4-95, FB4-14 revertant without SN marker (scarless). (**e**) GE4-128, FB4-14 revertant without SN marker (scarless). (**f**) B117-3, wild-type DEB-specific iPSC. (**g**) GE5-54, B117-3 revertant without SN marker (scarless). (**h**) GE5-65, B117-3 revertant without SN marker (scarless). The arrow indicates the position of the additional fragment. (**i**) GE5-72, B117-3 revertant without SN marker (scarless). (**j**) GE5-73, B117-3 revertant without SN marker (scarless). For more information, see Additional Data 5.

Supplementary Figure X6. Analysis of plasmid integration in the gene-edited clones. (**a**) Map showing the positions of primer pairs used to detect sequences derived from pY211-puro. Small arrows indicate the positions of the primers. (**b**) Assessment of the limits of detection of the primers used to detect pY211-puro. Labels of the horizontal axis indicate: top row, number of target DNA molecules per reaction tube; second row, concentration of pY211-puro template. Labels on the vertical axis indicate the target gene/region. The arrows indicate bands corresponding to the correct product from each reaction. At 1 aM, one reaction tube is estimated to contain about one molecule of the amplicon. (**c**) Plasmid integration assay. The numbers above the lanes indicate the ID numbers of the edited clones, while the target genes/regions are indicated on the vertical axis. Abbreviations: GE, gene editing; PC1, positive control 1 (90 molecules/tube); PC2, positive control 2 (9 molecules/tube); PC3, positive control 3 (0.9 molecule/tube); NC, negative control (UltraPure DNase/RNase-Free Distilled Water (Thermo Fisher Scientific/Invitrogen, USA)). **, strong band; *, weak band. For more information, see Additional Data 6.

Supplementary Figure X7. Fluorescence immunostaining for pluripotent gene markers in parental cells. (**a**) Fluorescence immunostaining for pluripotent gene markers in FB4-14 cells. 1st

raw: fluorescence immunostaining of markers using Alexa488-conjugated secondary antibodies. 2nd raw: DAPI staining. 3rd raw: phase-contrast image. Bar = 100 μm. (**b**) Fluorescence immunostaining for pluripotent gene markers in B117-3 cells. 1st raw: fluorescence immunostaining of markers using Alexa488-conjugated secondary antibodies. 2nd raw: DAPI staining. 3rd raw: phase-contrast image. Bar = 100 μm. For more information, see Additional Data 7.

Supplementary Figure X8. Examples of sequences around the target site of the gene-edited clones as determined by direct Sanger sequencing (secondary screening). Figures show the sequence of the target site, the classification and number of the classification, and the ID numbers of the clones. (**a**) Results of GE2, repair of MEN2B with a SN marker (One-SHOT). The results were classified into four classes: original (row 1), repaired (row 2), repaired but containing a deletion (rows 3 and 4), and simple deletion (rows 5 and 6). The red C indicates the mutant nucleotide in the mutant allele. A green C indicates the single nucleotide marker derived from the repair template (ssODN_RET_M918_I913silentC) and the 3′-terminus end nucleotide of the forward primer for SNMD-PCR. (**b**) Results of GE4, repair of MEN2B without a SN marker (scarless One-SHOT). The results were classified as: original (row 1), repaired (row 2), and deletion (rows 3–13). The results classified as "deletion" were divided into three further subclasses: deletion in both alleles (row 3), deletion in the target allele containing the forward primer binding site for SNMD-PCR (rows 4–9), and deletion in the target allele without the forward primer binding site for SNMD-PCR (rows 10–13). A red C indicates the mutant nucleotide in the mutant allele and the 3′-terminus end nucleotide of the forward primer for negative SNMD-PCR. Abbreviations: WT, wild-type allele; target, target allele; N, numbers of the clones. The blue T indicates the wild-type nucleotide in the wild-type allele. The red C indicates the mutant nucleotide in the mutant allele. Small letters indicate an insertion. For more information, see Additional Data 8.

Supplementary Figure X9. Expression of SeV markers and typical pluripotency markers determined by RT-PCR in FB4-14 and B117-3. The size of each amplicon is as follows : SeV-1st (181 bp), SeV-nested (148 bp), OCT4/POU5F1 (144 bp), SOX2 (151 bp), KLF4 (357 bp), C-MYC (328 bp), NANOG (391 bp), REX1 (306 bp), SALL4 (138 bp), GDF3 (631 bp), DNMT3B (242 bp), and β-ACTIN (455 bp). Abbreviations. M, marker. PC, positive control (cDNA of SeV-infected monocytes for SeV-1st and SeV-nested. cDNA of normal iPSC (409B2) for other targets). DW, distilled water.

References

1. Hsiau, T. *et al*. Inference of CRISPR Edits from Sanger Trace Data. *bioRxiv* (2018). doi: 10.1101/251082.
2. Yu, C. *et al*. Small Molecules Enhance CRISPR Genome Editing in Pluripotent Stem Cells. *Cell Stem Cell* **16**, 142-147 (2015).
3. Lin, S. *et al*. Nocodazole Enhanced homology-directed human genome engineering by controlled timing of CRISPR/Cas9 delivery. *eLife* **3**, e04766 (2014).
4. Chu, V. T. *et al.* Increasing the efficiency of homology-directed repair for CRISPR-Cas9-induced precise gene editing in mammalian cells. *Nat. Biotechnol.* 33, 543-548 (2015).
5. Maruyama, T. *et al*. Increasing the efficiency of precise genome editing with CRISPR-Cas9 by inhibition of nonhomologous end joining. *Nat. Biotechnol*. 33, 538-542 (2015).
6. Robert, F. *et al*. Pharmacological inhibition of DNA-PK stimulates Cas9-mediated genome editing. *Genome Med*. 7, 93 (2015).
7. Ma, X. *et al*. Small molecules promote CRISPR-Cpf1-mediated genome editing in human pluripotent stem cells. *Nat. Commun*. **9**, 1303 (2018).
8. Cullot, G. *et al.* CRISPR-Cas9 genome editing induces megabase-scale chromosomal truncations. *Nat. Commun*. **10**, 1136 (2019).
9. Poisson, P. S. D. Recherches sur la probabilité des Jugements*, Des Regles Generares du Calcul des Probabilites, Bacheliar, Impremeur-Libraire* (1857).
10. Gao, L. *et al*. Engineered Cpf1 variants with altered PAM specificities. *Nat. Biotechnol.* **35,** 789–792 (2017).
11. Kumar, A. *et al*. High-throughput methods for the large-scale analysis of gene function by transposon tagging. *Methods Enzymol.* **328,** 550–574 (2000).
12. de Felipe, P. *et al*. *E unum pluribus*: multiple proteins from a self-processing polyprotein. *Trends Biotechnol.* **24,** 68–75 (2006).
13. Kim, J. H. *et al*. High cleavage efficiency of a 2A peptide derived from porcine teschovirus-1 in human cell lines, zebrafish and mice. *PLoS One* **6,** e18556 (2011).
14. Kremers, G. J. *et al*. Improved green and blue fluorescent proteins for expression in bacteria and mammalian cells. *Biochemistry* **46,** 3775–3783 (2007).
15. Perez-Gonzalez, J. A., Vara, J. & Jimenez, A. The mechanism of resistance to

puromycin and to the puromycin-precursor O-demethyl-puromycin in *Streptomyces alboniger*. *J. Gen. Microbiol.* **131,** 2877–2883 (1985).

16. Zetsche, B. *et al*. Cpf1 is a single RNA-guided endonuclease of a class 2 CRISPR-Cas system. *Cell* **163,** 759–771 (2015).
17. Fujie, Y. *et al*. New type of Sendai virus vector provides transgene-free iPS cells derived from chimpanzee blood. *PLoS One* **9,** e113052 (2014).
18. Soga, M. *et al*. HPGCD outperforms HPBCD as a potential treatment for Niemann-Pick disease type C during disease modeling with iPS cells. *Stem Cells* **33,** 1075–1088 (2015).
19. Takahashi, K. *et al*. Induction of pluripotent stem cells from adult human fibroblasts by defined factors. *Cell* **131,** 861–872 (2007).
20. Vouillot, L., Thelie, A. & Pollet, N. Comparison of T7E1 and surveyor mismatch cleavage assays to detect mutations triggered by engineered nucleases. *G3 (Bethesda)* **5,** 407–415 (2015).
21. Schneider, C. A., Rasband, W. S. & Eliceiri, K. W. NIH Image to ImageJ: 25 years of image analysis. *Nat. Methods* **9,** 671–675 (2012).
22. Ran, F. A. *et al*. Genome engineering using the CRISPR-Cas9 system. *Nat. Protoc.* **8,** 2281–2308 (2013).
23. Nomura, N. *et al*. PCR-RFLP: a new HLA-DNA typing method tested in bone marrow transplantation. *Transplant Proc.* **23,** 431–433 (1991).
24. Ran, F. A. Adaptation of CRISPR nucleases for eukaryotic applications. *Anal. Biochem.* **532,** 90–94 (2017).
25. Labun, K. *et al*. CHOPCHOP v2: a web tool for the next generation of CRISPR genome engineering. *Nucleic Acids Res.* **44,** W272–276 (2016).
26. Maglott, D. R. *et al*. NCBI's LocusLink and RefSeq. *Nucleic Acids Res.* **28,** 126–128 (2000).
27. O'Leary, N. A. *et al*. Reference sequence (RefSeq) database at NCBI: current status, taxonomic expansion, and functional annotation. *Nucleic Acids Res.* **44,** D733–745 (2016).
28. Kent, W. J. *et al*. The human genome browser at UCSC. *Genome Res.* **12,** 996–1006 (2002).
29. Tyner, C. *et al*. The UCSC Genome Browser database: 2017 update. *Nucleic Acids Res.* **45,** D626–D634 (2017).

30. Li, H. L. *et al*. Efficient genomic correction methods in human iPS cells using CRISPR-Cas9 system. *Methods* **101,** 27–35 (2016).
31. Uemura, M. *et al.* Obesity and mental health improvement following nutritional education focusing on gut microbiota composition in Japanese women: a randomised controlled trial. *Eur J Nutr*. **58**, 3291-3302 (2019).
32. Andrews, S. FastQC: a quality control tool for high throughput sequence data. (2010). http://www.bioinformatics.babraham.ac.uk/projects/fastqc
33. Bolger, A. M. *et al*. Trimmomatic: A flexible trimmer for Illumina Sequence Data. *Bioinformatics*, btu170 (2014).
34. Langmead, B. & Salzberg, S. Fast gapped-read alignment with Bowtie 2. *Nat. Methods* **9**, 357-359 (2012).
35. Quinlan, A. R. & Hall, I. M. BEDTools: a flexible suite of utilities for comparing genomic features. *Bioinformatics* **26**, 841–842 (2010).
36. Magoč, T. & Salzberg, S. L. FLASH: fast length adjustment of short reads to improve genome assemblies. *Bioinformatics* 27, 2957–2963 (2011).
37. Connelly, J. P. & Pruett-Miller, S. M. CRIS.py: A Versatile and High-throughput Analysis Program for CRISPR-based Genome Editing. *Sci. Rep.* **9**, 4194 (2019).
38. Kumar, S. *et al*. MEGA X: Molecular Evolutionary Genetics Analysis across Computing. *Mol. Biol. Evol.* 35, 1547–1549 (2018).
39. Kwart, D. *et al*. Precise and efficient scarless genome editing in stem cells using CORRECT. *Nat. Protoc*. **12,** 329–354 (2017).

Supplementary Tables

pY211-puro construction	Forward (ID#)	Forward (SEQ)	Reverse (ID#)	Reverse (SEQ)
HAx3	1806	GCAAAAAAGAAAAAGGATCCTACCCATACGATGTTCCAGATTACG	1807	CCCCTGCCCTCGCCGGAGCCGCTAGCGGCATAGTCGGGGACATCATATG
T2A	1801	GGCTCCGGCGAGGGCAGGGGAAGTCTTTTGACATGCGGGGACGTGGAGGAAAATCCCGGCCCA	1802	TGGGCCGGGATTTTCCTCCACGTCCCCGCATGTCAAAAGACTTCCCCTGCCCTCGCCGGAGCC
SGFP2 amplification	1808	TGGAGGAAAATCCCGGCCCAACTAGTGTGAGCAAGGGCGAGGAGCTGTT	1809	ATCAGCGAGCTCTAGGAATTCTTAGTCGACCTTGTACAGCTCGTCCATGCCGAGA
puromycin-resistant gene amplification	1835	TCCCGGCCCAACTAGTACCGAGTACAAGCCCACGGTG	1836	CGAGCTCTAGGAATTCTCAGGCACCGGGCTTGCGGGT
pY211 sequencing after In-Fusion	1738	TGGCCTACATCCAGGAGCTG	1753	TGGCACCTTCCAGGGTCAAG
pY211 sequencing after In-Fusion	1814	TACCTGAGCACCCAGTCCAA		
pY211 sequencing after puroR insertion	1752	CTCCAATCAGGACTGGCTGG	1753	TGGCACCTTCCAGGGTCAAG

crRNA guide sequence template, and gene-editing template

RET exon16 M918	Forward (ID#)	Forward (SEQ)	Reverse (ID#)	Reverse (SEQ)
pY211puro-RET_M918_Tg1+	1757	agatAGTTAAATGGATGGCAATTG	1758	aaaaCAATTGCCATCCATTTAACT
pY211puro-RET_M918_Tg1m	1763	agatAGTTAAATGGACGGCAATTG	1764	aaaaCAATTGCCGTCCATTTAACT
pY211puro-RET_M918_Tg2+	1759	agatATTGCCATCCATTTAACTGG	1760	aaaaCCAGTTAAATGGATGGCAAT
pY211puro-RET_M918_Tg2m	1765	agatATTGCCGTCCATTTAACTGG	1766	aaaaCCAGTTAAATGGACGGCAAT
guide sequence template sequencing	1754	ACTATCATATGCTTACCGT		
RET_M918 target site PCR amplification	1751	GGGAAGCACTGCTCTGCACTAC	1747	TGCTCAGGGCCAGTGCAATT
RET_M918 target site sequencing	1744	GTGTGTGGCCAGTTCTGTGC		

ssODN for RET_M918 I913silentC (WT)	1854	TTATTCCATCTTCTCTTTAGGGTCGGATCCCAGTTAAATGGATGGCAATTGAATCCCTTTTTGATCATATCTACACCACGCAAAGTGATGTGTAAGTGT		
ssODN for RET_M918T I913silentC (Mut)	1855	TTATTCCATCTTCTCTTTAGGGTCGGATCCCAGTTAAATGGACGGCAATTGAATCCCTTTTTGATCATATCTACACCACGCAAAGTGATGTGTAAGTGT		
ssODN for RET_M918 I920silentC (WT)	1857	TTATTCCATCTTCTCTTTAGGGTCGGATTCCAGTTAAATGGATGGCAATCGAATCCCTTTTTGATCATATCTACACCACGCAAAGTGATGTGTAAGTGT		
ssODN for RET_M918 (WT)	1794	TTATTCCATCTTCTCTTTAGGGTCGGATTCCAGTTAAATGGATGGCAATTGAATCCCTTTTTGATCATATCTACACCACGCAAAGTGATGTGTAAGTGT		
RET_I913 SNMD-PCR primer WT	1791	CCATCTTCTCTTTAGGGTCGGATT	1747	TGCTCAGGGCCAGTGCAATT
RET_I913 SNMD-PCR primer Mut	1856	CCATCTTCTCTTTAGGGTCGGATC	1747	TGCTCAGGGCCAGTGCAATT
RET_M918 SNMD-PCR primer WT	1748	GGGTCGGATTCCAGTTAAATGGAT	1747	TGCTCAGGGCCAGTGCAATT
RET_M918 SNMD-PCR primer Mut	1749	GGGTCGGATTCCAGTTAAATGGAC	1747	TGCTCAGGGCCAGTGCAATT
RET_I920 SNMD-PCR primer WT	1859	GGATTCCAGTTAAATGGATGGCAATC	1747	TGCTCAGGGCCAGTGCAATT
RET_I920 SNMD-PCR primer Mut	1860	GATTCCAGTTAAATGGACGGCAATC	1747	TGCTCAGGGCCAGTGCAATT
RET_M918 off-target1 amplification	1863	TTCATTTACAGGCGTACTTCG	1865	ATGACTACCGGTTTCCCAAT
RET_M918 off-target2 amplification	1878	CGCCTGTAATCCCAGTTACT	1879	AAAGAGCTATGGTTCCTTGCTCTG
RET_M918 off-target1 sequencing	1871	ACTACCGGTTTCCCAATGAA		
RET_M918 off-target2 sequencing	1873	GTTACTCAGGAGGCTGAGGC		
COL7A1 exon78 G2138	Forward (ID#)	Forward (SEQ)	Reverse (ID#)	Reverse (SEQ)
pY211puro-COL7A1_G2138_Tg1+	1893	agatGGCTCTCCCCGGTCTCCTTT	1905	aaaaAAAGGAGACCGGGGAGAGCC
pY211puro-COL7A1_G2138_Tg1m	1899	agatGGCTCTCCCCGGTCTCATTT	1908	aaaaAAATGAGACCGGGGAGAGCC
pY211puro-COL7A1_G2138_Tg2+	1895	agatCGGTCTCCTTTGATGCCTGG	1906	aaaaCCAGGCATCAAAGGAGACCG

pY211puro-COL7A1_G2138_Tg2m	1901	agatCGGTCTCATTTGATGCCTGG	1909	aaaaCCAGGCATCAAATGAGACCG
pY211puro-COL7A1_G2138_Tg3+	1897	agatGGTCTCCTTTGATGCCTGGC	1907	aaaaGCCAGGCATCAAAGGAGACC
pY211puro-COL7A1_G2138_Tg3m	1903	agatGGTCTCATTTGATGCCTGGC	1910	aaaaGCCAGGCATCAAATGAGACC
COL7A1_G2138 target site PCR amplification	1882	TCTGTGGATGAGCCAGGTCCTG	1886	CCTTAGTTTCCCAGTTCCAACTTCC
COL7A1_G2138 target site sequencing	1888	GGTGACCAAGGTCCCAAAGG		
ssODN for COL7A1_G2138 (WT)	1911	CTTACCGGGTTGCCGTCCTGACCCCTCGGTC CAGGCTCTCCCCGGTCTCCTTTGATGCCTGG CACACCCTGAAGGCAGAGTGTCGTGCCCTGA GCCCCC		
ssODN for COL7A1_G2138X (Mut)	1912	CTTACCGGGTTGCCGTCCTGACCCCTCGGTC CAGGCTCTCCCCGGTCTCATTTGATGCCTGG CACACCCTGAAGGCAGAGTGTCGTGCCCTGA GCCCCC		
COL7A1_G2138 SNMD-PCR primer WT	1913	GGGTGTGCCAGGCATCAAAG	1886	CCTTAGTTTCCCAGTTCCAACTTCC
COL7A1_G2138 SNMD-PCR primer Mut	1914	GGGTGTGCCAGGCATCAAAT	1886	CCTTAGTTTCCCAGTTCCAACTTCC
COL7A1_G2138 off-target1 amplification	1918	TCACGGAGGCAGGACCATC	1919	GGGCAGACATGAGGTGATCCAT
COL7A1_G2138 off-target2 amplification	1920	CCTAGTCACGGAGGCAGGACC	1921	TGGGCAGACATGAGGTGATCC
COL7A1_G2138 off-target3 amplification	1922	CCGGCACTGGCAGGCTTT	1923	CGGAACTGTGGGAGGCAAG
COL7A1_G2138 off-target4 amplification	1924	TCATAGTGATAGAAGCATCCCTCACA	1925	AACAGAGCAAGACTCTGTCTCTAAAATAAA
COL7A1_G2138 off-target5 amplification	1926	AGCTTTGGAAGCACCATGAGG	1927	GCATGGGGTGATGGGAACCT
COL7A1_G2138 off-target6 amplification	1928	GTTGATCTGAAGAACCATGGGAGC	1929	ACTACTGGTCCCTGGGGTCTGT
COL7A1_G2138 off-target7 amplification	1930	AGGCTCCATCTCCTCAGTGCA	1931	CAGTTTTGTGTCACTCCCTCAGGT
COL7A1_G2138 off-target1 sequencing	1932	ACCATCCACCAGCATCCCTG		
COL7A1_G2138 off-target2 sequencing	1937	AGGACCATCCACCAGCATCC		
COL7A1_G2138 off-target3 sequencing	1940	AACGGAACTGTGGGAGGCAAGG		
COL7A1_G2138 off-target4 sequencing	1941	AAAAATGAGGCAGATAGAAA		
COL7A1_G2138 off-target5 sequencing	1944	GCGTGTGTGCAGGGGTCAAA		
COL7A1_G2138 off-target6 sequencing	1947	ATATTTGCGAAGATCTGAGC		
COL7A1_G2138 off-target7 sequencing	1952	TGCAAAGCCCTGCCTGACCT		

iPSC marker	Forward (ID#)	Forward (SEQ)	Reverse (ID#)	Reverse (SEQ)
SeV	1401	GGATCACTAGGTGATATCGAGC	1402	ACCAGACAAGAGTTTAAGAGATATGTATC
nested	1403	TCGAGCCATATGACAGCTCG	1404	GAGATATGTATCCTTTTAAATTTTCTTGTCTTC TTG
OCT3/4	1405	GACAGGGGGAGGGGAGGAGCTAGG	1406	CTTCCCTCCAACCAGTTGCCCCAAAC
SOX2	1407	GGGAAATGGGAGGGGTGCAAAAGAGG	1408	TTGCGTGAGTGTGGATGGGATTGGTG
KLF4	1409	GATTACGCGGGCTGCGGCAAAACCTACACA	1410	TGATTGTAGTGCTTTCTGGCTGGGCTCC
c-MYC	1411	GCGTCCTGGGAAGGGAGATCCGGAGC	1412	TTGAGGGGCATCGTCGCGGGAGGCTG
NANOG	1413	CAGCCCCGATTCTTCCACCAGTCCC	1414	CGGAAGATTCCCAGTCGGGTTCACC
GDF3	1415	CTTATGCTACGTAAAGGAGCTGGG	1416	GTGCCAACCCAGGTCCCGGAAGTT
REX1	1417	CAGATCCTAAACAGCTCGCAGAAT	1418	GCGTACGCAAATTAAAGTCCAGA
SALL4	1419	AAACCCCAGCACATCAACTC	1420	GTCATTCCCTGGGTGGTTC
DNMT3B	1421	TGCTGCTCACAGGGCCCGATACTTC	1422	TCCTTTCGAGCTCAGTGCACCACAAAAC
β-ACTIN	1423	CAACCGCGAGAAGATGAC	1424	AGGAAGGCTGGAAGAGTG

Supplementary Table S1. Primers and ssODNs used in this study.

[a]All primers are written from 5’ to 3’.

[b] CRISPR RNA guide sequence templates are denoted in uppercase.

[c]BbsI sites for CRISPR RNA template construction in pY211-puro are denoted in lower-case.

[d] Underlines show substituted bases.

Sample	Site	Genomic location	No. of mis-matches	Sequence [a] (including mismatches)	Indel ratio (%)[b]
original	COL7A1 exon 78 target1	chr3: 48574512	0	TCCAGGCTCTCCCCGGTCTCCTTT	
GE5	off-target 1	chr11: 61210396	3	TTCAGGgcCTCCCCGGTCTCCcTT	0/6 (0 %)
GE5	off-target 2	chr11: 61229225	3	TTCAGGgcCTCCCCGGTCTCCcTT	0/6 (0 %)
GE5	off-target 3	chr12: 47759197 (- strand)	3	TCCCcGCTCTCCCCGGTtTCCTTg	0/6 (0 %)
GE5	off-target 4	chr20: 17295309 (- strand)	3	TCCAGGCTCTgCCCaGTCTCCTTg	0/6 (0 %)
GE5	off-target 5	chr3: 48580596	3	TCCAGGaTCTCCCtGGTCTCCcTT	0/6 (0 %)
GE5	off-target 6	chr3: 141471637	3	TTCAGGCTtTgCaCGGTCTCCTTT	0/6 (0 %)
GE5	off-target 7	chr6_Kl270802v1_ alt:69348	3	TTCGGGCTCTtCCCtGTCTCCTTc	0/6 (0 %)

Supplementary Table S2. Summary of off target-effects generated by AsCpf1_RR in B117-3 cells.

[a]PAMs in the original target site and the putative off-target sites are underlined. The lower letters indicate mismatched bases.

[b]Off-target regions were amplified from the genomic DNA of the intended gene-edited clones of the iPSCs, and directly sequenced. The number of indel clones relative to the number of analyzed clones are indicated.

Evaluation items	One-SHOT	scarless One-SHOT	CORRECT (2017)	MhAX (2018)
Time for editing[a]	3 weeks	3 weeks	2 months	2 months
Cost[b]	low	low	high	high
Simplicity: template design[c]	○ (codon degeneracy for generating silent mutation)	◎ (simple replacement)	△ (codon degeneracy, blocking base and the distance effect)	△ (codon degeneracy and MMEJ）
Simplicity: template construction[d]	◎ (ssODN)	◎ (ssODN)	◎ (ssODN)	△ (dsDNA from 3 fragments)
Simplicity: 1st screening[e]	◎ (SNMD-PCR)	◎ (SNMD-PCR)	△ (PCR-RFLP)	△ (PCR-RFLP)
Quality: scar around target[f]	△ (SNS)	◎ (scarless)	◎ (scarless)	△ (SNS)
Quality: off-target[g]	◎ (BGL)	◎ (BGL)	△ (significant)	△(dsDNA insertion by NHEJ)
Applicable genome region[h]	△ (CDS only）	◎（All）	◎（All）	△（CDS only）
Substitution rate[i]	2-6 %	2-8 %	1st round: 2-3 %<br>2nd round: 1-17 %	1st round: N.D.<br>2nd round: 11-36%

Supplementary Table S3. Comparison of One-SHOT, scarless One-SHOT, CORRECT and MhAX.

[a]Time required to identify the intended genome-edited iPSCs after electroporation.

[b]The cost is estimated based on the time to establish gene-edited clones.

[c]Words/phrases in parentheses indicate the knowledge required for designing the templates.

[d]Words in parentheses indicate the material and/or method for template generation.

[e]Words/phrases in parentheses indicate the method for the first round of screening.

[f]Words/phrases in parentheses indicate the degree: SNS: single nucleotide substitution; scarless: no substitution.

[g]Words/phrases in parentheses indicate the degree and additional genomic disturbance. BGL: background level.

[h]CDS: coding sequence; All: coding and noncoding sequence of the genome.

[i]Percentage of intended clones/ first screening -passed clones.

Amplicon Name/ Size	Chromosomal location of CORE sequence	CORE sequence*	Primer ID#	Extended Primer Sequence** (Top: Foward/ Bottom: Reverse)	Amplicon Terminal Sequence (Top: Start/ Bottom: Stop)
RET_MEN2B_Target	chr10: 43121953	TTCCAGTTAAATGGATGGCAATTG	2043	TCGTCGGCAGCGTCAGATGTGTATAAGAGACAG CCATGCATAGGGAAGCACTGC	CCATGCATAGGGAAGCACTGC
377 bps			2044	GTCTCGTGGGCTCGGAGATGTGTATAAGAGACAG CCACACTTACACATCACTTTGCGTG	CACGCAAAGTGATGTGTAAGTGTGG
RET_Off_Target1	chr15: 91512242	CAATTGCCAACCAATTAACGGGAA	2045	TCGTCGGCAGCGTCAGATGTGTATAAGAGACAG TTCATTTACAGGCGTACTTCG	TTCATTTACAGGCGTACTTCG
364 bps			2066	GTCTCGTGGGCTCGGAGATGTGTATAAGAGACAG CCTTGAACCTATCTCTTGCTGCTTTT	AAAAGCAGCAAGAGATAGGTTCAAGG
RET_Off_Target2	chr4: 128631982	CAAATGCCATGCATTTAAGTGGAA	2047	TCGTCGGCAGCGTCAGATGTGTATAAGAGACAG CGCCTGTAATCCCAGTTACT	CGCCTGTAATCCCAGTTACT
362 bps			2048	GTCTCGTGGGCTCGGAGATGTGTATAAGAGACAG AAAGAGCTATGGTTCCTTGCTCTG	CAGAGCAAGGAACCATAGCTCTTT
COL7A1_DEB_Target	chr3: 48574512	TCCAGGCTCTCCCCGGTCTCCTTT	2051	TCGTCGGCAGCGTCAGATGTGTATAAGAGACAG GAGCCGGGCAGCAATGGT	GAGCCGGGCAGCAATGGT
375 bps			2052	GTCTCGTGGGCTCGGAGATGTGTATAAGAGACAG CCAACTTCCCCTCCACCCA	TGGGTGGAGGGGAAGTTGG
COL7A1_DEB_Off_Target1	chr11: 61210396	TTCAGGGCCTCCCCGGTCTCCCTT	2053	TCGTCGGCAGCGTCAGATGTGTATAAGAGACAG TCACGGAGGCAGGACCATC	TCACGGAGGCAGGACCATC
378 bps			2054	GTCTCGTGGGCTCGGAGATGTGTATAAGAGACAG GGGCAGACATGAGGTGATCCAT	TGGATCACCTCATGTCTGCCC
COL7A1_DEB_Off_Target2	chr11: 61229225	TTCAGGGCCTCCCCGGTCTCCCTT	2055	TCGTCGGCAGCGTCAGATGTGTATAAGAGACAG CCTAGTCACGGAGGCAGGACC	CCTAGTCACGGAGGCAGGACC
384 bps			2056	GTCTCGTGGGCTCGGAGATGTGTATAAGAGACAG TGGGCAGACATGAGGTGATCC	GGATCACCTCATGTCTGCCCA
COL7A1_DEB_Off_Target3	chr12: 47759197 (-)	(+)CAAGGAAACCGGGGAGAGCGGGGA	2057	TCGTCGGCAGCGTCAGATGTGTATAAGAGACAG CCGGCACTGGCAGGCTTT	CCGGCACTGGCAGGCTTT
372 bps		(-) TCCCCGCTCTCCCCGGTTTCCTTG	2058	GTCTCGTGGGCTCGGAGATGTGTATAAGAGACAG CGGAACTGTGGGAGGCAAG	CTTGCCTCCCACAGTTCCG
COL7A1_DEB_Off_Target4	chr20: 17295309 (-)	(+) CAAGGAGACTGGGCAGAGCCTGGA	2059	TCGTCGGCAGCGTCAGATGTGTATAAGAGACAG TCATAGTGATAGAAGCATCCCTCACA	TCATAGTGATAGAAGCATCCCTCACA
353 bps		(-) TCCAGGCTCTGCCCAGTCTCCTTG	2068	GTCTCGTGGGCTCGGAGATGTGTATAAGAGACAG AAAAATTAGGGACAAATCTGTAGAAGTAGC	GCTACTTCTACAGATTTGTCCCTAATTTTT
COL7A1_DEB_Off_Target5	chr3: 48580596	TCCAGGATCTCCCTGGTCTCCCTT	2060	TCGTCGGCAGCGTCAGATGTGTATAAGAGACAG AGCTTTGGAAGCACCATGAGG	AGCTTTGGAAGCACCATGAGG
383 bps			2061	GTCTCGTGGGCTCGGAGATGTGTATAAGAGACAG GCATGGGGTGATGGGAACCT	AGGTTCCCATCACCCCATGC
COL7A1_DEB_Off_Target6	chr3: 141471637	TTCAGGCTTTGCACGGTCTCCTTT	2062	TCGTCGGCAGCGTCAGATGTGTATAAGAGACAG GTTGATCTGAAGAACCATGGGAGC	GTTGATCTGAAGAACCATGGGAGC
414 bps			2063	GTCTCGTGGGCTCGGAGATGTGTATAAGAGACAG ACTACTGGTCCCTGGGGTCTGT	ACAGACCCCAGGGACCAGTAGT
COL7A1_DEB_Off_Target7	chr6_Kl270802v1_alt:69348	TTCGGGCTCTTCCCTGTCTCCTTC	2064	TCGTCGGCAGCGTCAGATGTGTATAAGAGACAG AGGCTCCATCTCCTCAGTGCA	AGGCTCCATCTCCTCAGTGCA
389 bps			2065	GTCTCGTGGGCTCGGAGATGTGTATAAGAGACAG CAGTTTTGTGTCACTCCCTCAGGT	ACCTGAGGGAGTGACACAAAACTG

Supplementary Table X1. Data set for AmpliSeq analysis.

Note. *: predicted by CHOPCHOP v2. **: underline, sequence tag for primary PCR of AmpliSeq analysis.

Target	seq_start	seq_end	test_list1	test_list2	ref_seq
RET_MEN2B_Target	AGAGAGTTAG	GTAAGTGTGG	TTCCAGTTAAATGGATGGCAATTG	TTCCAGTTAAATGGATGGCAATCG	CCATGCATAGGGAAGCACTGCTCTGCACTACCAGCAGGCCTGTGGCATGTGACAAGCTGGCCCTGTGTGCCTGTGGGTGGGCAGCTGACTCCCGCCAGCATCTCAGCAATCCACAGGAGGTTCAGGCTGGAGCTCCAGCCCCTTCAAAGATGTGTGTGGCCAGTTCTGTGCCCAGGAGTGTCTACAGCACTCCTCTGGTTACTGAAAGCTCAGGGATAGGGCCTGGCCTTCTCCTTTACCCCTCCTTCCTAGAGAGTTAGAGTAACTTCAATGTCTTTATTCCATCTTCTCTTTAGGGTCGGATTCCAGTTAAATGGATGGCAATTGAATCCCTTTTTGATCATATCTACACCACGCAAAGTGATGTGTAAGTGTGG
RET_Off_Target1	TGGAAACCAA	AACCCAATCA	CAATTGCCAACCAATTAACGGGAA		TTCATTTACAGGCGTACTTCGCAAGGGAATTAGCTCACTACAATTTGGCTTATTTTGTGCCCGAACTTAGCTTTTCCACACTCTTTTCTTCTATGAATATGAAAAGATGAAATTTAAGACTCTATAGCTGGAAAGGGCCCAGGGACACTTTTCACAAATGCTGAATATTTCACTCACTGTCAACTCTCTACCTTGGAAACCAAATCAAAATGCAACAATGAATCAACTACATCAAAAAATTCAGGTCAATTGCCAACCAATTAACGGGAACATCTGGTTATTTTGACATCTGGTCAAAACAGTTGACCGTTACAACCCAATCAGCATCAAAAATCTGATTCCATAATAAAAGCAGCAAGAGATAGGTTCAAGGT
RET_Off_Target2	GACTGAGACT	AACATACATA	CAAATGCCATGCATTTAAGTGGAA		CGCCTGTAATCCCAGTTACTCAGGAGGCTGAGGCAGGAGAATTGCTTGAACCCGGGAGGCAGAGGTTGCAGTGAGCCTAGATCACACCATTGCACTCCAGCCTGGGCAACAGACTGAGACTCCATCTCAAAAACAACAACAACAACAAAACATTAAATAGTATACAAATGCCATGCATTTAAGTGGAACACAGTGCCAACACATTACCAATTATTCCAAGCACATACATGAAACATACATATAGAAAGTATTGTTTCCAAATTGGAATGTGCATTCTACAAGGAAATATTTAAATCAAAATGAAATAGCGGGGCTTCATCCTTCTTTGGACAGTACTTTAAAGCAGAGCAAGGAACCATAGCTCTTT
COL7_DEB_Target	AGGGACTGGG	CCACACATGT	AAAGGAGACCGGGGAGAGCCTGGA	AAATGAGACCGGGGAGAGCCTGGA	GAGCCGGGCAGCAATGGTGACCAAGGTCCCAAAGGAGACAGGGTGAGGCCTCTCTCCACCCTTCCATAGAGTCCCCCTCCTTTCTGGGGGCACACTAGAGGTGGTGTGCATATGCACCTGGGCACGTGGCAGGGACTGGGGGCTCAGGGCACGACACTCTGCCTTCAGGGTGTGCCAGGCATCAAAGGAGACCGGGGAGAGCCTGGACCGAGGGGTCAGGACGGCAACCCGGTAAGTCCTTGCCCAACAGCCACACATGTGCAAGAAGGTGGCTCTCACATGTATTGTCCTGTGTGCAGGGCTGGGGTCTGTACTGCCTGGGACTGTCTGGGTCCTGACTCTGTCTAGGGGGATGGTGGGTGGAGGGGAAGTTGG
COL7_DEB_Off_Target1	GTGGGGCAAA	CGTGCTCAGT	TTCAGGGCCTCCCCGGTCTCCCTT	TTCGGGGCCTCCCCTGTCTCCCTG	TCACGGAGGCAGGACCATCCACCAGCATCCCTGGGGAGAGCTGGGGGACCCACCTGTCCACGCATCTGACAATTTAAGGGGCCGGTGAACCAGCACACGCTCCTGCCACTCGAGGAAACATGTCACTTTCCGTCTAGAAGTGGGGCAAAATGGCTAAGAAATTGTGTGAAGTCACTTCATTCCACCCTCCCCTTCAGGGCCTCCCCGGTCTCCCTTGCAGCTTCATTTCCACGCTCTTCTTCCCCAGCTAGCCTGCTAGCGTGCTCAGTGGTGTGGGTTTTCCCAAGAATGAACAATTTCCCAGGACAGAGACTTTCAGTACTAAAACTGGGACCAGCCCAGGCAAACCAGGACACATGGATCACCTCATGTCTGCCC
COL7_DEB_Off_Target2	GTGGGGCAAA	CGTGCTCAGT	TTCAGGGCCTCCCCGGTCTCCCTT	TTCGGGGCCTCCCCTGTCTCCCTG	CCTAGTCACGGAGGCAGGACCATCCACCAGCATCCCTGGGGAGAGCTGGGGGACCCACCTGTCCACGCATCTGACAATTTAAGGGGCCGGTGAACCAGCACACGCTCCTGCCACTCGAGGAAACATGTCACTTTCCGTCTAGAAGTGGGGCAAAATGGCTAAGAAATTGTGTGAAGTCACTTCATTCCACCCTCCCCTTCAGGGCCTCCCCGGTCTCCCTTGCAGCTTCATTTCCACGCTCTTCTTCCCCAGCTAGCCTGCTAGCGTGCTCAGTGGTGTGGGTTTTCCCAAGAATGAACAATTTCCCAGGACAGAGACTTTCAGTACTAAAACTGGGACCAGCCCAGGCAAACCAGGACACATGGATCACCTCATGTCTGCCCA
COL7_DEB_Off_Target3	CCGGCGGTGA	TGGGGCCGCT	CAAGGAAACCGGGGAGAGCGGGGA		CCGGCACTGGCAGGCTTTGGAGTCCGCACCCGGGGGTACCAGAGTGGACATCCGCCTGAGCCCAGAGTATCCCCAGGGGGCAGGAGCGGCCCCGAAGTGTGTGGAGGAGGCCGCAACGGCCGGGGTGTCAGGGATCCGGCGGTGAGTGGCTGCACTGGCCGCCACGTGGCCGGGAGGAGAAATGCAAACAAGGAAACCGGGGAGAGCGGGGAGGGGGTGAGTCACTGCGGCCTCACCCGGGAGGGAAATCCGGGCTGGGGCCGCTTCCCGGCTGGGGGTGTCTGCCTCCTGTTTCGGTTCAGGGTCTCCAGCCGCCCGGGGGAGGGAATAGGCGGCTTTCCCGCGGGGAATCCCGCTGCCTTGCCTCCCACAGTTCCGTTCC
COL7_DEB_Off_Target4	AGTTTAATGC	ACCGTGCAAT	CAAGGAGACTGGGCAGAGCCTGGA		TCATATCATAGTGATAGAAGCATCCCTCACATTTCACTGACATTCAAAATTGTAATAATCTTCCCTGCATTTTCAGTTGTTTCAGTGAGGTCTGCATTTATACGCAGTTAACACACACACACACACACACACACACACACACCTCTCTCAAGAGTTTAATGCATGCCAGATATTGTTTTAAATGCCATTATTACCACTTCCCAAGCAAGGAGACTGGGCAGAGCCTGGAAGCCAACCCAGACAGTCTGATTCAGAGCCAGGAACACTCTACCACCGTGCAATACCAGCATTTGTTCTTTACGCTCAGCTGGGTCTCCTATTTCAAGTTGCTACTTCTACAGATTTGTCCCTAATTTTT
COL7_DEB_Off_Target5	GTTAAGGTTG	CAGCTGGCCT	TCCAGGATCTCCCTGGTCTCCCTT		AGAGCTTTGGAAGCACCATGAGGACTCATGGGAATGTTGGTAGCCTTCAGATGCGTGTGTGCAGGGGTCAAAGGAAGTGAAGATTGGGAGGGTTTAGCATTACAGGGTTGGGGGGTAGGATCAGGTATTGGGAATTGGCTGGTTGGAGGGTTAAGGTTGGGGTGAGGAGTCATAGGCTGGGACTCACATTTCGTCCATCCTCTCCAGGATCTCCCTGGTCTCCCTTTTCACCCACAGGCCCCCGAACTCCAGGTGCCCCCTAAGAAGAGCAGCTGGCCTGAGACAGACCCTCCCAATATTTTGCAGGTGCCCCTATGACCCGCTACACTGCCCCAGGTTCCCCATTACTCCAAAATCCACATCAGAGGTTCCCATCACCCCATGC
COL7_DEB_Off_Target6	TTTTGCTCTC	CCGCAGCCAG	TTCAGGCTTTGCACGGTCTCCTTT		GTTGATCTGAAGAACCATGGGAGCATTGTCCACAATATTTGCGAAGATCTGAGCCGTCAGGTCCTTGATGGTCTTAAAGTAATGGCCCCAGTCTCTGATCTGGGGTCCCTTCTTCTCCAGGTGCTCCCGGATTTTGCTCTCCAGCTTCTGGTTCTCTGTCTTCAGGTAGGAGGCCAGGCAGTTGTTCAGGCTTTGCACGGTCTCCTTTTCATTTTAGATGCCTCCCATTCTTACTAGACCCCCGGCCATCCCCGCAGCCAGGCCCCTGGGCCCCAAGCCACCCCGGAAGCTGATGGAGTGGGACACAGAGATCCAGAAACCAAAGCCCCCCCATGGCTGCATAGACACTGGCCATGCTGCTGACCAGCCAGGTGCTGTAGCTGGGTGCCTGGACAGACCCCAGGGACCAGTAGT
COL7_DEB_Off_Target7	TTGTTACTAG	GAGGCAGGAA	TTCGGGCTCTTCCCTGTCTCCTTC		AGGCTCCATCTCCTCAGTGCAAATGCAAAGCCCTGCCTGACCTCCCAGGTTGATTTCCCCAAAGAAAAGACACTCCACAAGGCCCAGCAGGGTGCAGCTCTGCACTCTGCATGCCGCGCCTCCCCACCCACCTTTGTTACTAGGAAACATCGACCTCCCTTTGGCTACCTGGTAGACCTTTGCCTGTTCGGGCTCTTCCCTGTCTCCTTCAGGTGTCAACTGATACCTCTCCCCTGCCTCCCATACACAGAGTGAGGCAGGAACCACACTCCCAGGGTGGACCAGAGCTGGCCGAGTCTTTTGCAGCTTCCTACTGGGCAGGGGACCCACACCTGCTCATCAGAAATTGTTGCTTGGAATGTGATACCTGAGGGAGTGACACAAAACTG

Supplementary Table X2. Data set for Bioinfomatics analysis.

Gene Editing Condition					Sanger Sequencing		ICE Analysis							
SEQ No.	Group	Sample Well ID No.	Cell	Plasmid (crRNA)	Read?	Noise by Indel	Name of ICE data file	Guide Seq. used in ICE	ab1 file (Control)	ab1 file (Experiment)	Indel (%)	R^2	KO-Score	Trace Condition
1	Group A	A-3	409B2 (WT)	pY211-puro-crRNA_RET-1+	y	+++	200301_1s	RET-1+	200228_#9	200228_#1	67	0.95	32	OK
1b	do.	do.	do.	do.	y	+++	200317_1bs	do.	200305_#9	200305_#1	68	0.96	32	OK
2	do.	A-4	do.	do.	y	+++	200301_2s	do.	200228_#9	200228_#2	70	0.95	33	OK
2b	do.	do.	do.	do.	y	+++	200317_2bs	do.	200305_#9	200305_#2	71	0.96	33	OK
3	do.	A-7	do.	pY211-puro-crRNA_RET-1m	y	-	200301_3s	do.	200228_#9	200228_#3	0	1	ND	OK
3b	do.	do.	do.	do.	y	-	200317_3bs	do.	200305_#9	200305_#3	0	1	ND	OK
4	do.	A-8	do.	do.	y	-	200302_4s	do.	200228_#9	200228_#4	0	1	ND	OK
4b	do.	do.	do.	do.	y	-	200317_4bs	do.	200305_#9	200305_#4	0	1	ND	OK
5	do.	A-11	do.	pY211-puro-crRNA_RET-2+	y	+	200302_5s	RET-2+	200228_#9	200228_#5	3	0.99	ND	OK
5b	do.	do.	do.	do.	y	+	200317_5bs	do.	200305_#9	200305_#5	3	0.99	ND	OK
6	do.	A-12	do.	do.	y	+	200302_6s	do.	200228_#9	200228_#6	6	0.99	ND	OK
6b	do.	do.	do.	do.	y	+	200317_6bs	do.	200305_#9	200305_#6	6	0.99	ND	OK
7	do.	A-15	do.	pY211-puro-crRNA_RET-2m	y	-	200306_7s	do.	200228_#9	200306_#1	0	1	ND	OK
7b	do.	do.	do.	do.	y	-	200317_7bs	do.	200305_#9	200305_#7	0	1	ND	OK
8	do.	A-16	do.	do.	y	-	200306_8s	do.	200228_#9	200306_#2	0	1	ND	OK
8b	do.	do.	do.	do.	y	-	200317_8bs	do.	200305_#9	200305_#8	0	1	ND	OK
9	do.	A-19	do.	NC	y	-	200301_9s	RET-1+	200228_#9	200228_#9	0	1	ND	OK
9b	do.	do.	do.	do.	y	-	200317_9bs	do.	200305_#9	200305_#9	0	1	ND	OK
10	do.	A-20	do.	do.	y	-	200301_10s	do.	200228_#9	200228_#10	0	0.88	ND	OK
10b	do.	do.	do.	do.	y	-	200317_10bs	do.	200305_#9	200305_#10	0	1	ND	OK
11	Group C	C-3	GE3-9 (MEN2B homo)	pY211-puro-crRNA_RET-1+	y	-	200301_11s	RET-1m	200228_#19	200228_#11	0	1	ND	OK
11b	do.	do.	do.	do.	y	-	200317_11bs	do.	200305_#19	200305_#11	0	1	ND	OK
12	do.	C-4	do.	do.	y	-	200301_12s	do.	200228_#19	200228_#12	0	1	ND	OK
12b	do.	do.	do.	do.	y	-	200317_12bs	do.	200305_#19	200305_#12	0	1	ND	OK
13	do.	C-7	do.	pY211-puro-crRNA_RET-1m	y	++	200306_13s	do.	200228_#19	200306_#3	20	0.98	12	OK
13b	do.	do.	do.	do.	y	++	200317_13bs	do.	200305_#19	200305_#13	21	0.98	12	OK
14	do.	C-8	do.	do.	y	++	200306_14s	do.	200228_#19	200306_#4	16	0.98	7	OK
14b	do.	do.	do.	do.	y	++	200317_14bs	do.	200305_#19	200305_#14	16	0.98	7	OK
15	do.	C-11	do.	pY211-puro-crRNA_RET-2+	y	-	200301_15s	RET-2m	200228_#19	200228_#15	0	1	ND	OK
15b	do.	do.	do.	do.	y	-	200317_15bs	do.	200305_#19	200305_#15	0	1	ND	OK
16	do.	C-12	do.	do.	y	-	200301_16s	do.	200228_#19	200228_#16	0	1	ND	OK
16b	do.	do.	do.	do.	y	-	200317_16bs	do.	200305_#19	200305_#16	0	1	ND	OK
17	do.	C-15	do.	pY211-puro-crRNA_RET-2m	y	+	200301_17s	do.	200228_#19	200228_#17	0	1	ND	OK
17b	do.	do.	do.	do.	y	+	200317_17bs	do.	200305_#19	200305_#17	0	1	ND	OK
18	do.	C-16	do.	do.	y	+	200302_18s	do.	200228_#19	200228_#18	0	1	ND	OK
18b	do.	do.	do.	do.	y	+	200317_18bs	do.	200305_#19	200305_#18	0	1	ND	OK
19	do.	C-19	do.	NC	y	-	200301_19s	RET-1m	200228_#19	200228_#19	0	1	ND	OK
19b	do.	do.	do.	do.	y	-	200317_19bs	do.	200305_#19	200305_#19	0	1	ND	OK
20	do.	C-20	do.	do.	y	-	200301_20s	do.	200228_#19	200228_#20	0	1	ND	OK
20b	do.	do.	do.	do.	y	-	200317_20bs	do.	200305_#19	200305_#20	0	1	ND	OK
21	Group B	B-3	409B2 (WT)	pY211-puro-crRNA_COL7A1-1+	y	++	200301_21s	COL7A1-1+	200228_#33	200228_#21	23	0.94	22	OK
21b	do.	do.	do.	do.	y	++	200317_21bs	do.	200305_#33	200305_#21	23	0.94	22	OK
22	do.	B-4	do.	do.	y	++	200301_22s	do.	200228_#33	200228_#22	21	0.95	16	OK
22b	do.	do.	do.	do.	y	++	200317_22bs	do.	200305_#33	200305_#22	22	0.95	17	OK
23	do.	B-7	do.	pY211-puro-crRNA_COL7A1-1m	y	-	200301_23s	do.	200228_#33	200228_#23	0	1	ND	OK
23b	do.	do.	do.	do.	y	-	200317_23bs	do.	200305_#33	200305_#23	0	1	ND	OK
24	do.	B-8	do.	do.	y	-	200301_24s	do.	200228_#33	200228_#24	0	1	ND	OK
24b	do.	do.	do.	do.	y	-	200317_24bs	do.	200305_#33	200305_#24	0	1	ND	OK
25	do.	B-11	do.	pY211-puro-crRNA_COL7A1-2+	y	+	200302_25s	COL7A1-2+	200228_#33	200228_#25	6	0.99	6	OK
25b	do.	do.	do.	do.	y	+	200317_25bs	do.	200305_#33	200305_#25	6	0.99	6	OK
26	do.	B-12	do.	do.	y	+	200302_26s	do.	200228_#33	200228_#26	5	0.99	5	OK
26b	do.	do.	do.	do.	y	+	200317_26bs	do.	200305_#33	200305_#26	5	0.99	5	OK
27	do.	B-15	do.	pY211-puro-crRNA_COL7A1-2m	y	-	200301_27s	do.	200228_#33	200228_#27	0	1	ND	OK
27b	do.	do.	do.	do.	y	-	200317_27bs	do.	200305_#33	200305_#27	0	1	ND	OK
28	do.	B-16	do.	do.	y	-	200301_28s	do.	200228_#33	200228_#28	0	1	ND	OK
28b	do.	do.	do.	do.	y	-	200317_28bs	do.	200305_#33	200305_#28	0	1	ND	OK
29	do.	B-19	do.	pY211-puro-crRNA_COL7A1-3+	y	+	200302_29s	COL7A1-3+	200228_#33	200228_#29	0	1	ND	OK
29b	do.	do.	do.	do.	y	+	200317_29bs	do.	200305_#33	200305_#29	0	1	ND	OK
30	do.	B-20	do.	do.	y	+	200302_30s	do.	200228_#33	200228_#30	0	1	ND	OK
30b	do.	do.	do.	do.	y	+	200317_30bs	do.	200305_#33	200305_#30	0	1	ND	OK
31	do.	B-23	do.	pY211-puro-crRNA_COL7A1-3m	y	-	200301_31s	do.	200228_#33	200228_#31	0	1	ND	OK
31b	do.	do.	do.	do.	y	-	200317_31bs	do.	200305_#33	200305_#31	0	1	ND	OK
32	do.	B-24	do.	do.	y	-	200302_32s	do.	200228_#33	200228_#32	0	1	ND	OK
32b	do.	do.	do.	do.	y	-	200317_32bs	do.	200305_#33	200305_#32	0	1	ND	OK
33	do.	B-27	do.	NC	y	-	200301_33s	COL7A1-1+	200228_#33	200228_#33	0	1	ND	OK
33b	do.	do.	do.	do.	y	-	200317_33bs	do.	200305_#33	200305_#33	0	1	ND	OK
34	do.	B-28	do.	do.	y	-	200301_34s	do.	200228_#33	200228_#34	0	1	ND	OK
34b	do.	do.	do.	do.	y	-	200317_34bs	do.	200305_#33	200305_#34	0	1	ND	OK

Supplementary Table X3. Summary of ICE analysis. The gene editing conditions, the results of Sanger sequencing, and the results of ICE analysis are shown. Guide sequences used in ICE analysis: RET-1+, AGTTAAATGGATGGCAATTG; RET-1m, AGTTAAATGGACGGCAATTG; RET-2+, ATTGCCATCCATTTAACTGG; RET-2m, ATTGCCGTCCATTTAACTGG; COL7A1-1+, GGCTCTCCCCGGTCTCCTTT; COL7A1-2+, CGGTCTCCTTTGATGCCTGG; COL7A1-3+, GGTCTCCTTTGATGCCTGGC. Abbreviations: do., ditto (the same as above); NC, negative control; ND, not determined; +++, strong; ++, obvious; +, weak; −, negative.

Serial No. (S_)	Experiment	Gene Editing ID	Purpose (Gene Editing)	Cell	Plasmid	crRNA	Template	Target	Read Counts	Total_Indel (%)	Substituted with the template (%)	Identical to WT allele (%)	Identical to mutant allele (%)
1	3_On target effect	GE1re	modification	FB4-14	pY211-puro-crRNA_RET-1+	RET-1+	ssODN_RET_M918T_I913silentC	RET_MEN2B_Target	5271	21.1	2.54	18.3*	52.7
2	3_On target effect	GE2re	repair	FB4-14	pY211-puro-crRNA_RET-1m	RET-1m	ssODN_RET_M918_I913silentC	RET_MEN2B_Target	5803	21.2	2.45	52.4	18.6*
3	3_On target effect	GE3re	repair	FB4-14	pY211-puro-crRNA_RET-1m	RET-1m	ssODN_RET_M918_I920silentC	RET_MEN2B_Target	3051	12	5.44	54.3	22.0*
4	3_On target effect	GE4re	repair (scarless)	FB4-14	pY211-puro-crRNA_RET-1m	RET-1m	ssODN_RET_M918	RET_MEN2B_Target	4067	11.5	ND	63.1**	18.6*
5	3_On target effect	GEre_NC	negative control	FB4-14	NT	-	NT	RET_MEN2B_Target	7917	0.7	ND	47.9	45.3
6	3_Off target effect	GE1re	modification	FB4-14	pY211-puro-crRNA_RET-1+	RET-1+	ssODN_RET_M918T_I913silentC	RET_Off_Target1	110324	0.3	NA	NA	NA
7	3_Off target effect	GE2re	repair	FB4-14	pY211-puro-crRNA_RET-1m	RET-1m	ssODN_RET_M918_I913silentC	RET_Off_Target1	14238	0.5	NA	NA	NA
8	3_Off target effect	GE3re	repair	FB4-14	pY211-puro-crRNA_RET-1m	RET-1m	ssODN_RET_M918_I920silentC	RET_Off_Target1	8397	0.4	NA	NA	NA
9	3_Off target effect	GE4re	repair (scarless)	FB4-14	pY211-puro-crRNA_RET-1m	RET-1m	ssODN_RET_M918	RET_Off_Target1	23588	0.4	NA	NA	NA
10	3_Off target effect	GEre_NC	negative control	FB4-14	NT	-	NT	RET_Off_Target1	96062	0.3	NA	NA	NA
11	3_Off target effect	GE1re	modification	FB4-14	pY211-puro-crRNA_RET-1+	RET-1+	ssODN_RET_M918T_I913silentC	RET_Off_Target2	23997	1.1	NA	NA	NA
12	3_Off target effect	GE2re	repair	FB4-14	pY211-puro-crRNA_RET-1m	RET-1m	ssODN_RET_M918_I913silentC	RET_Off_Target2	109744	0.9	NA	NA	NA
13	3_Off target effect	GE3re	repair	FB4-14	pY211-puro-crRNA_RET-1m	RET-1m	ssODN_RET_M918_I920silentC	RET_Off_Target2	31391	1	NA	NA	NA
14	3_Off target effect	GE4re	repair (scarless)	FB4-14	pY211-puro-crRNA_RET-1m	RET-1m	ssODN_RET_M918	RET_Off_Target2	21316	1	NA	NA	NA
15	3_Off target effect	GEre_NC	negative control	FB4-14	NT	-	NT	RET_Off_Target2	34181	0.9	NA	NA	NA

Serial No. (S_)	Experiment	Gene Editing ID	Purpose (Gene Editing)	Cell	Plasmid	crRNA	Template	Target	Read Counts	Total_Indel (%)	Substituted with the template (%)	Identical to WT allele (%)	Identical to mutant allele (%)
16	3_On target effect	GE5re	repair (scarless)	B117-3	pY211-puro-crRNA_COL7A1-1m	COL7A1-1m	ssODN_COL7A1_G2138	COL7A1_DEB_Target	1220	15.7	ND	51.4	30.0*
17	3_On target effect	GE5re_NC	negative control	B117-3	NT	-	NT	COL7A1_DEB_Target	9512	0.5	ND	48.6	46.8
18	3_Off target effect	GE5re	repair (scarless)	B117-3	pY211-puro-crRNA_COL7A1-1m	COL7A1-1m	ssODN_COL7A1_G2138	COL7A1_Off_Target1	3148	0.6	NA	NA	NA
19	3_Off target effect	GE5re_NC	negative control	B117-3	NT	-	NT	COL7A1_Off_Target1	20918	0.5	NA	NA	NA
20	3_Off target effect	GE5re	repair (scarless)	B117-3	pY211-puro-crRNA_COL7A1-1m	COL7A1-1m	ssODN_COL7A1_G2138	COL7A1_Off_Target2	34171	0.6	NA	NA	NA
21	3_Off target effect	GE5re_NC	negative control	B117-3	NT	-	NT	COL7A1_Off_Target2	11550	0.5	NA	NA	NA
22	3_Off target effect	GE5re	repair (scarless)	B117-3	pY211-puro-crRNA_COL7A1-1m	COL7A1-1m	ssODN_COL7A1_G2138	COL7A1_Off_Target3	7225	1.2	NA	NA	NA
23	3_Off target effect	GE5re_NC	negative control	B117-3	NT	-	NT	COL7A1_Off_Target3	5747	1.5	NA	NA	NA
24	3_Off target effect	GE5re	repair (scarless)	B117-3	pY211-puro-crRNA_COL7A1-1m	COL7A1-1m	ssODN_COL7A1_G2138	COL7A1_Off_Target4	2575	0	NA	NA	NA
25	3_Off target effect	GE5re_NC	negative control	B117-3	NT	-	NT	COL7A1_Off_Target4	2867	0	NA	NA	NA
26	3_Off target effect	GE5re	repair (scarless)	B117-3	pY211-puro-crRNA_COL7A1-1m	COL7A1-1m	ssODN_COL7A1_G2138	COL7A1_Off_Target5	73949	0.8	NA	NA	NA
27	3_Off target effect	GE5re_NC	negative control	B117-3	NT	-	NT	COL7A1_Off_Target5	857	0.7	NA	NA	NA
28	3_Off target effect	GE5re	repair (scarless)	B117-3	pY211-puro-crRNA_COL7A1-1m	COL7A1-1m	ssODN_COL7A1_G2138	COL7A1_Off_Target6	5557	0.5	NA	NA	NA
29	3_Off target effect	GE5re_NC	negative control	B117-3	NT	-	NT	COL7A1_Off_Target6	837	0.5	NA	NA	NA
30	3_Off target effect	GE5re	repair (scarless)	B117-3	pY211-puro-crRNA_COL7A1-1m	COL7A1-1m	ssODN_COL7A1_G2138	COL7A1_Off_Target7	814	0.4	NA	NA	NA
31	3_Off target effect	GE5re_NC	negative control	B117-3	NT	-	NT	COL7A1_Off_Target7	801	0.2	NA	NA	NA

Supplementary Table X4. Examination of on- and off-target effects by AmpliSeq analysis. Top, examinations of the genome editing toolsets for RET_MEN2B target (RET_M918 site) and the two predicted off-target sites. Bottom, examinations of the genome editing toolset for COL7A1_exon78_DEB target (COL7A1_G2138 site) and the predicted seven off-target sites. Abbreviations: WT, wild-type; GE, gene-editing experiment; NC, negative control; NT, not transfected; ND, not detectable; NA, not applicable; *, decrease ; **, increase. Note. See Extended Results for results regarding *As* Cas12a_RR_RET_tgt1m.

***As* Cas12a_RR with crRNA_RET-1+ in a homology-directed repair (HDR) situation for RET_M918 modification**

To demonstrate the target cleavage activity of *As* Cas12a_RR with crRNA_RET-1+ in a homology-directed repair (HDR) situation for RET_M918 modification, a genome editing tool set (pY211-puro-crRNA_RET-1+ plus ssODN_RET_M918T_I913silentC for modification) was introduced into FB4-14 MEN2B-specific iPSC (GE1re). The indel rate in the target sequence (RET_MEN2B_Target) was then calculated by AmpliSeq analysis. While the indel rate in the negative control was 0.7% (S5), the rate in GE1re cells was 21.1% (S1), indicating that selective cleavage of the target sequence occurs frequently. Next, to determine the off-target effects of *As* Cas12a_RR with crRNA_RET-1+, we performed AmpliSeq analysis of two off-target candidates (RET_Off-target1 and RET_Off-target2). For RET_Off-target1, the indel rate in the negative control was 0.3% (S10), whereas that in GE1re was 0.3% (S6). For RET_Off-target2, the indel rate in the negative control was 0.9% (S15), with a rate of 1.1% observed in GE1re (S11). These results indicate that the off-target effects of *As* Cas12a_RR with crRNA_RET-1+ are at or below the background level.

***As* Cas12a_RR with crRNA_COL7A1-1m in a HDR situation for COL7A1_DEB repair**

To demonstrate the target cleavage activity of *As* Cas12a_RR with crRNA_COL7A1-1m in a HDR situation for COL7A1_DEB repair, one genome editing tool set (pY211-puro with crRNA_COL7A1-1m plus ssODN_COL7A1_G2138 for repair) was introduced into B117-3 DEB-specific iPSC (GE5re). The indel rate in the target sequence (COL7A1_DEB_Target) was then calculated using AmpliSeq analysis. While the indel rate in the negative control was 0.5% (S17), the rate in GE5re cells was 15.7% (S16), suggesting that selective cleavage of the target sequence by *As* Cas12a_RR with crRNA_COL7A1-1m occurs frequently. AmpliSeq analysis was then performed using seven off-target candidates (COL7A1-Off-target1 to Off-target7) to determine the off-target effects of *As* Cas-12a _RR with crRNA_COL7A1-1m occurs frequently. AmpliSeq analysis was then performed using seven off-target candidates (COL7A1-Off-target1 to Off-target7) to determine the off-target effects of *As* Cas12a_RR with crRNA_COL7A1-1m. In all cases, the cleavage activities in the off-target sequences were similar to the negative control level (S18-S31), confirming that the off-target effects of *As* Cas12a_RR with crRNA_COL7A1-1m are at or below the background level.

Serial No.	ID No.	Name	Sequence	Length	Amplicon size (bp)	Amplicon Location on the Plasmid
1	2011	Cas12a-2Fwd	TCTCCGGCTTTTATGAGAACAGG	23	382	1898-2279
2	2012	Cas12a-2Rev	GCGATGATGTGGGCTGTCTCA	21		
3	2023	Cas12a-8Fwd	CGGCAGATACCGGGACCTGTAT	22	382	4902-5283
4	2024	Cas12a-8Rev	GTCCTGATTGGAGATGCCGTT	21		
5	2080	puroFwd2	ACGCGCCACACCGTCGAT	18	478	5617-6094
6	2081	puroRev2	ACCAGGTGCGCGGTCCTTC	19		
7	2086	ACTBgen_Fwd1	TGAGACCTTCAACACCCCAGC	21	350	NA
8	2089	ACTBgen_Revcom	CGTAGCTCTTCTCCAGGGAGGA	22		

Supplementary Table X5. Primer set for plasmid integration analysis.
Abbreviation: NA, not associated.

a

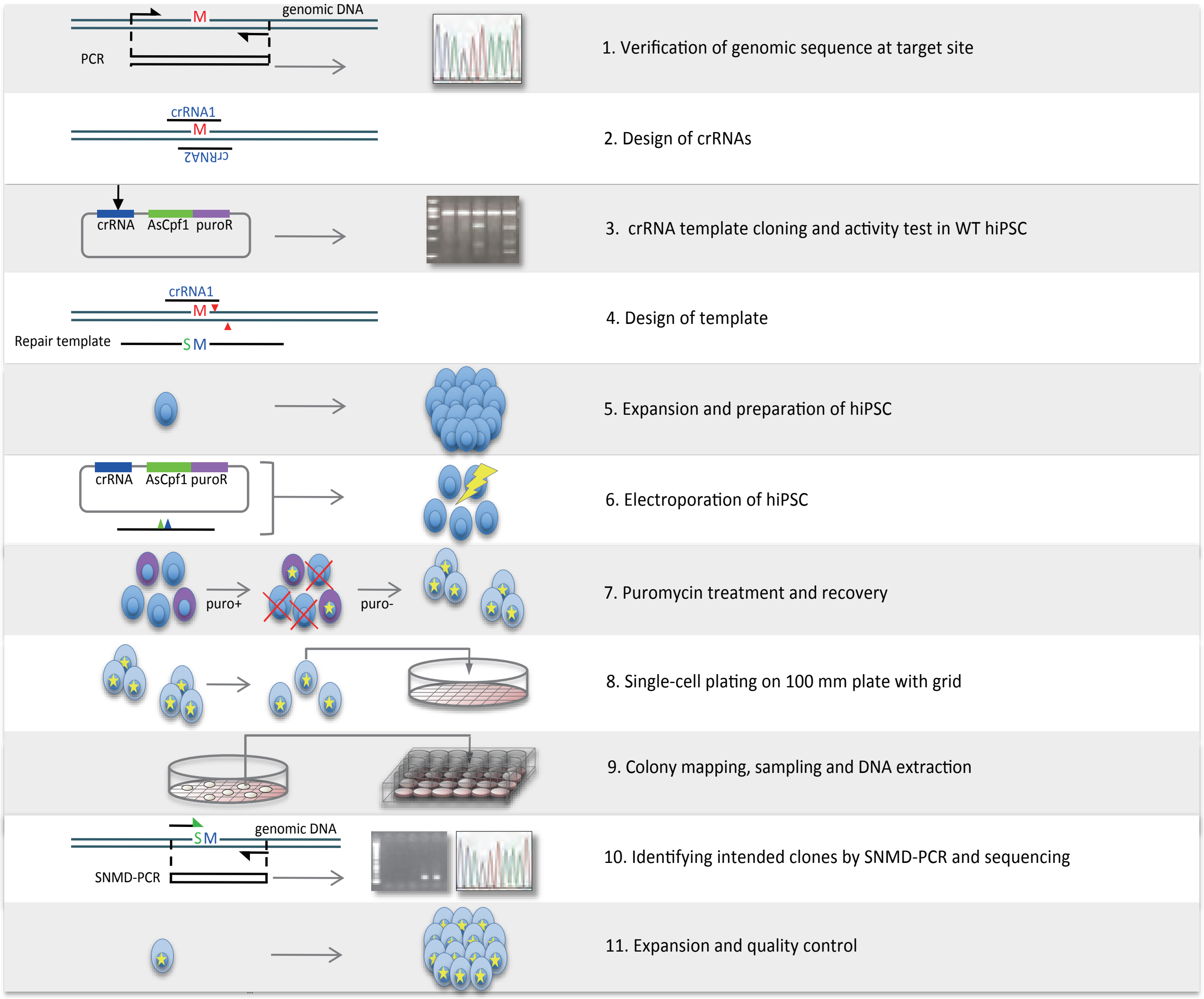

b

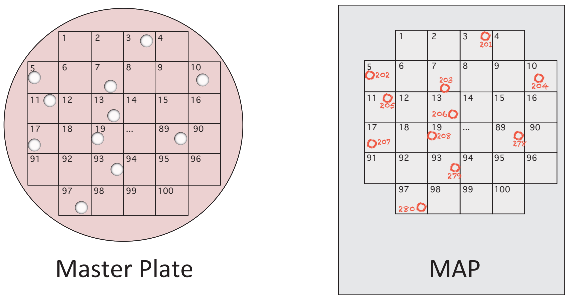

Supplementary Figure S1.

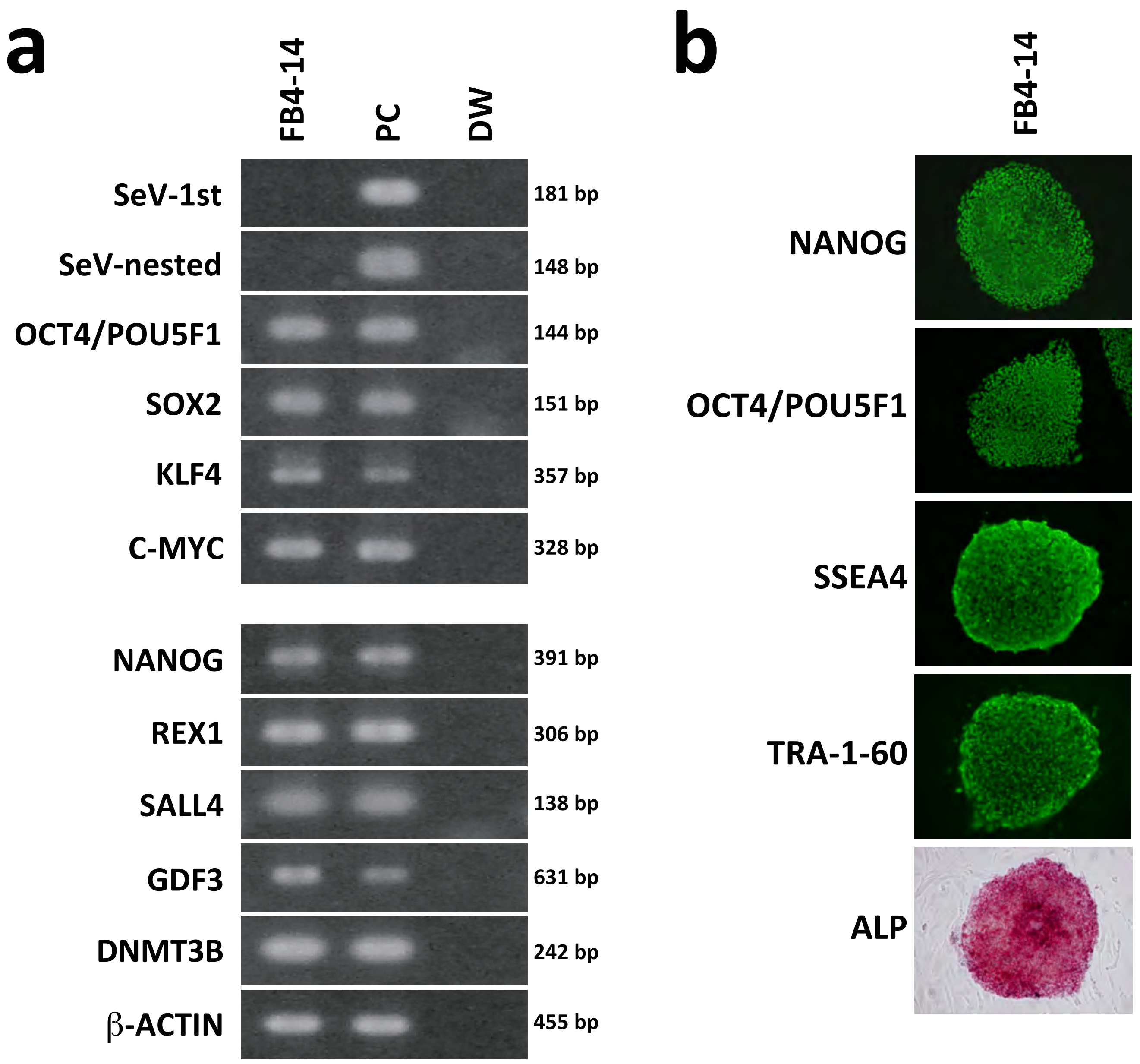

Supplementary Figure S2.

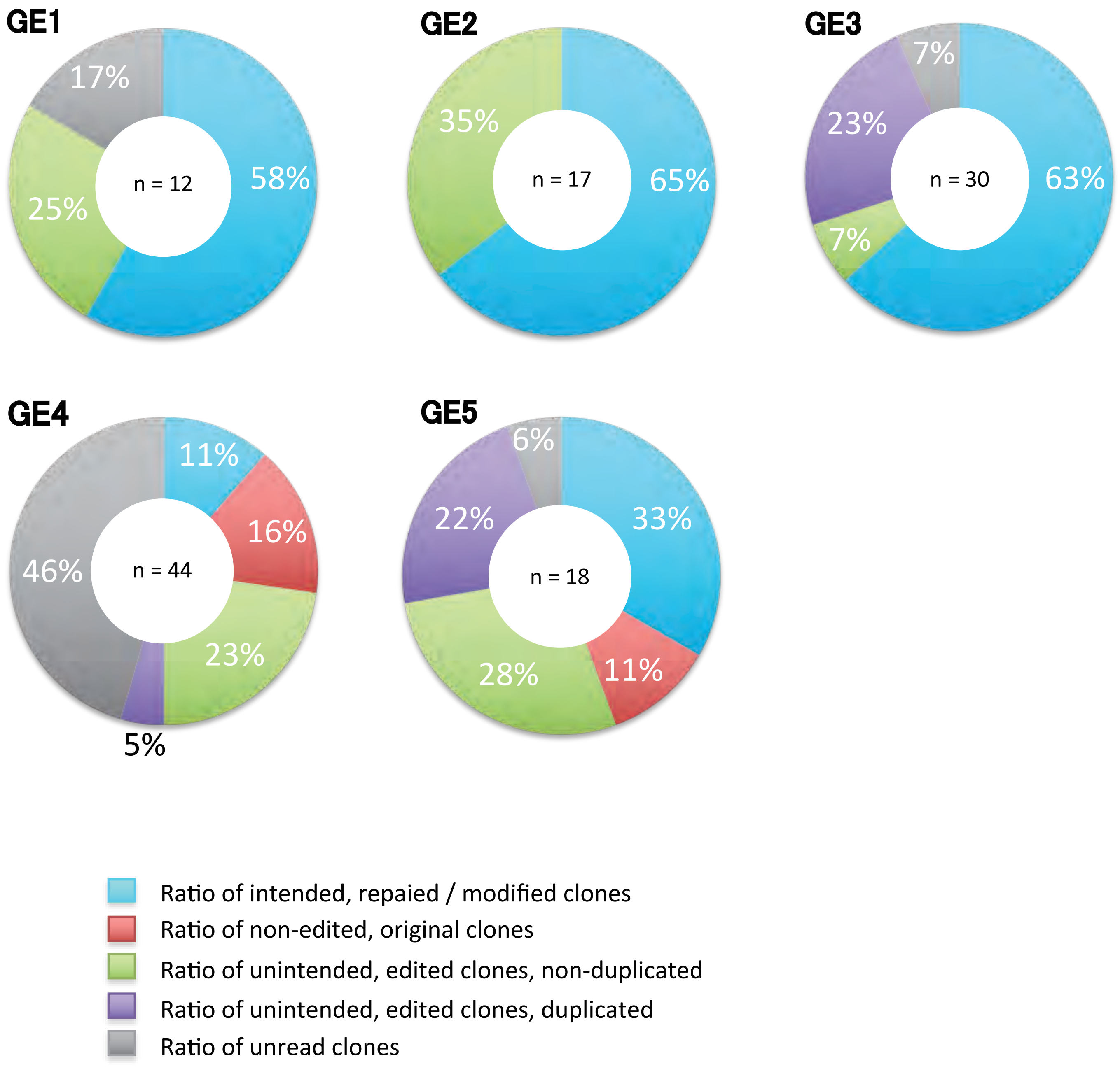

Supplementary Figure S3.

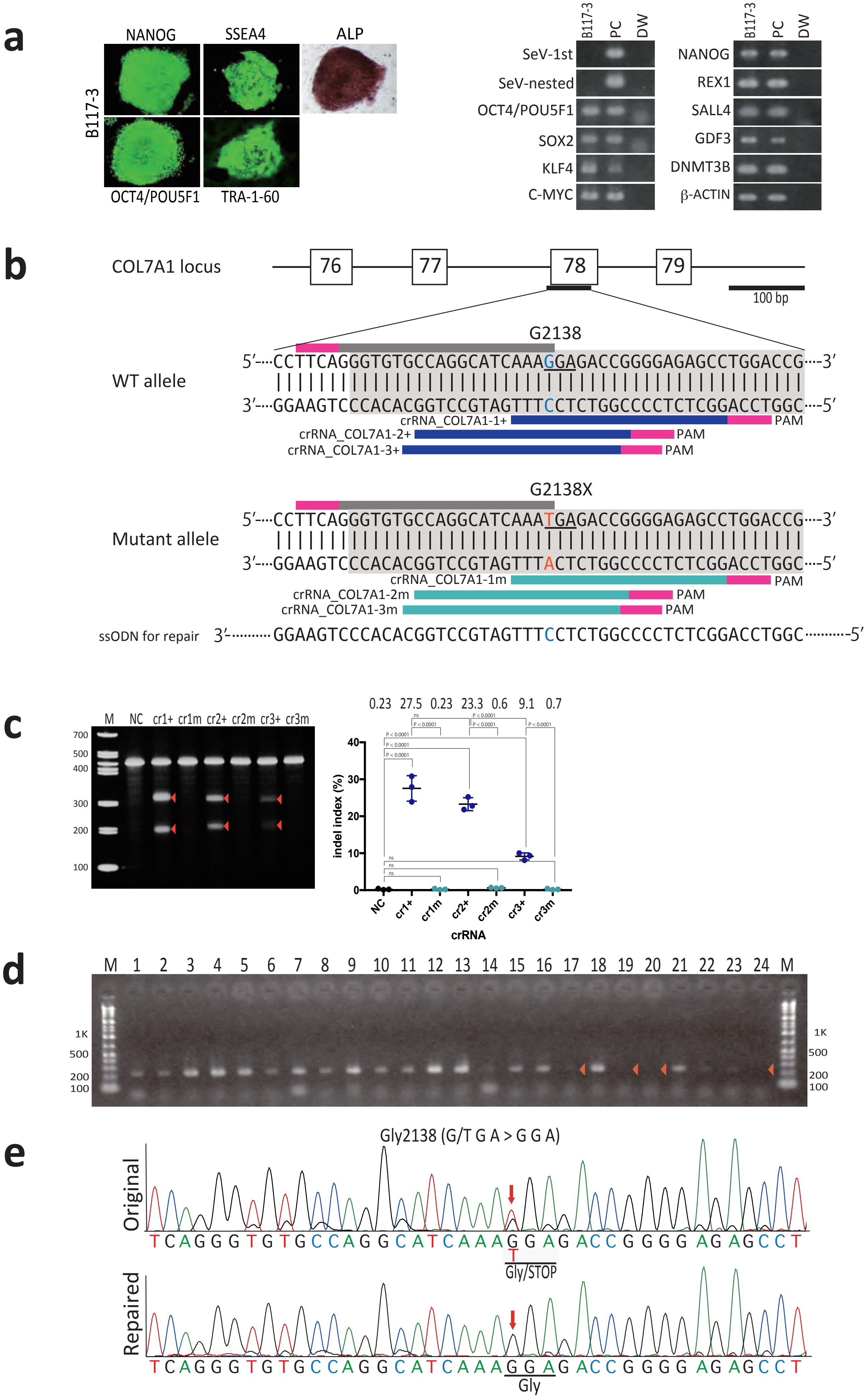

Supplementary Figure S4.

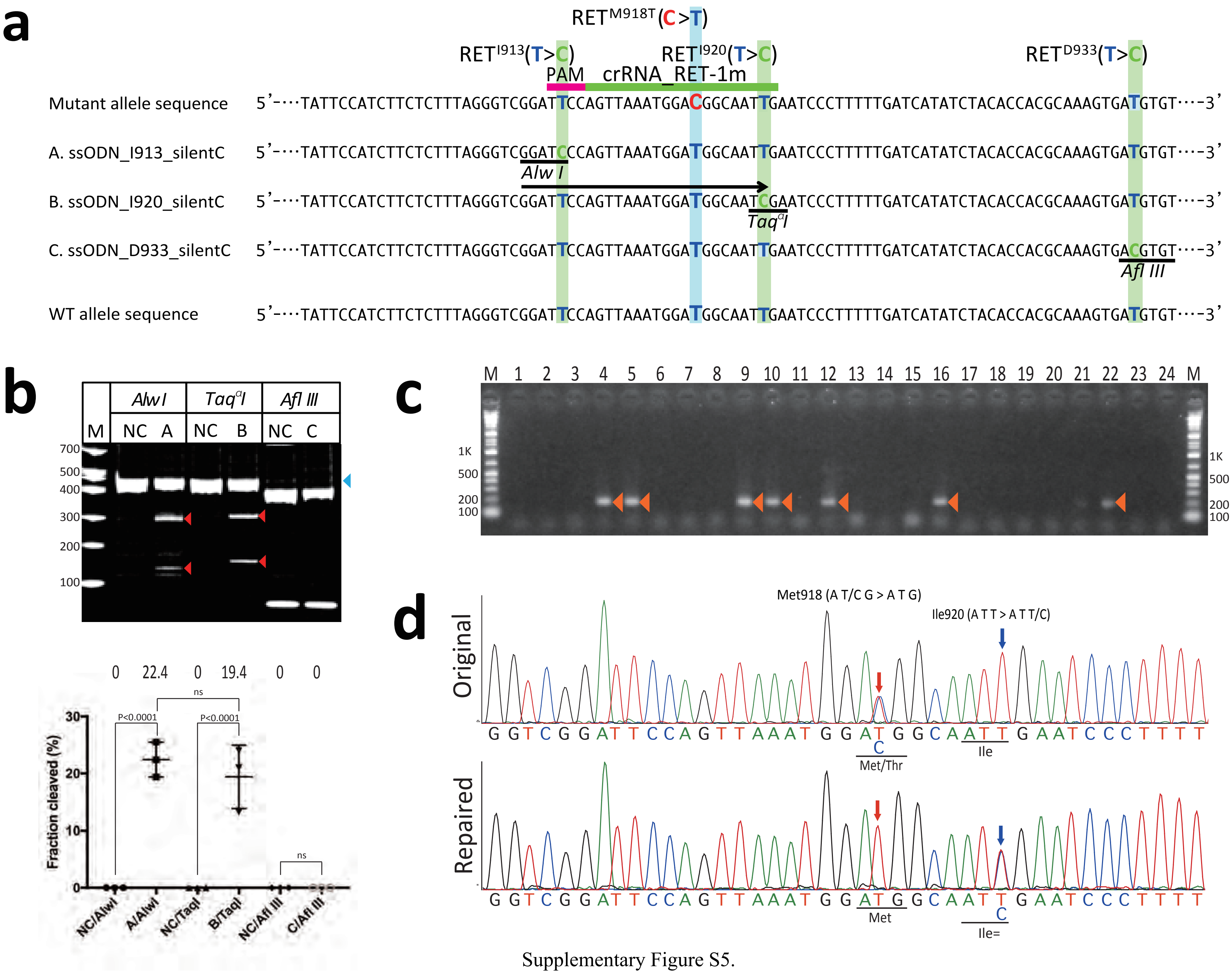

Supplementary Figure S5.

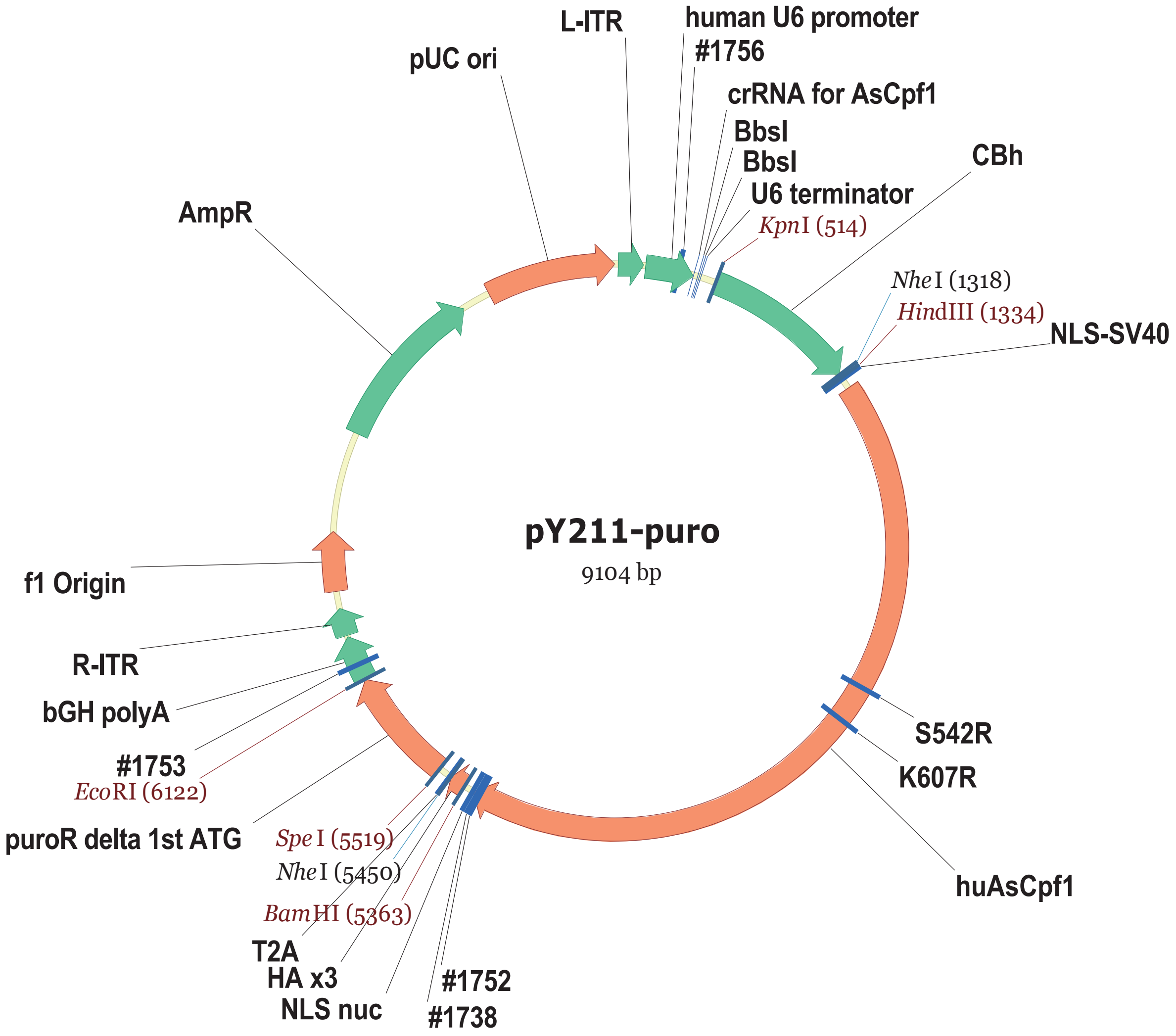

Supplementary Figure S6.

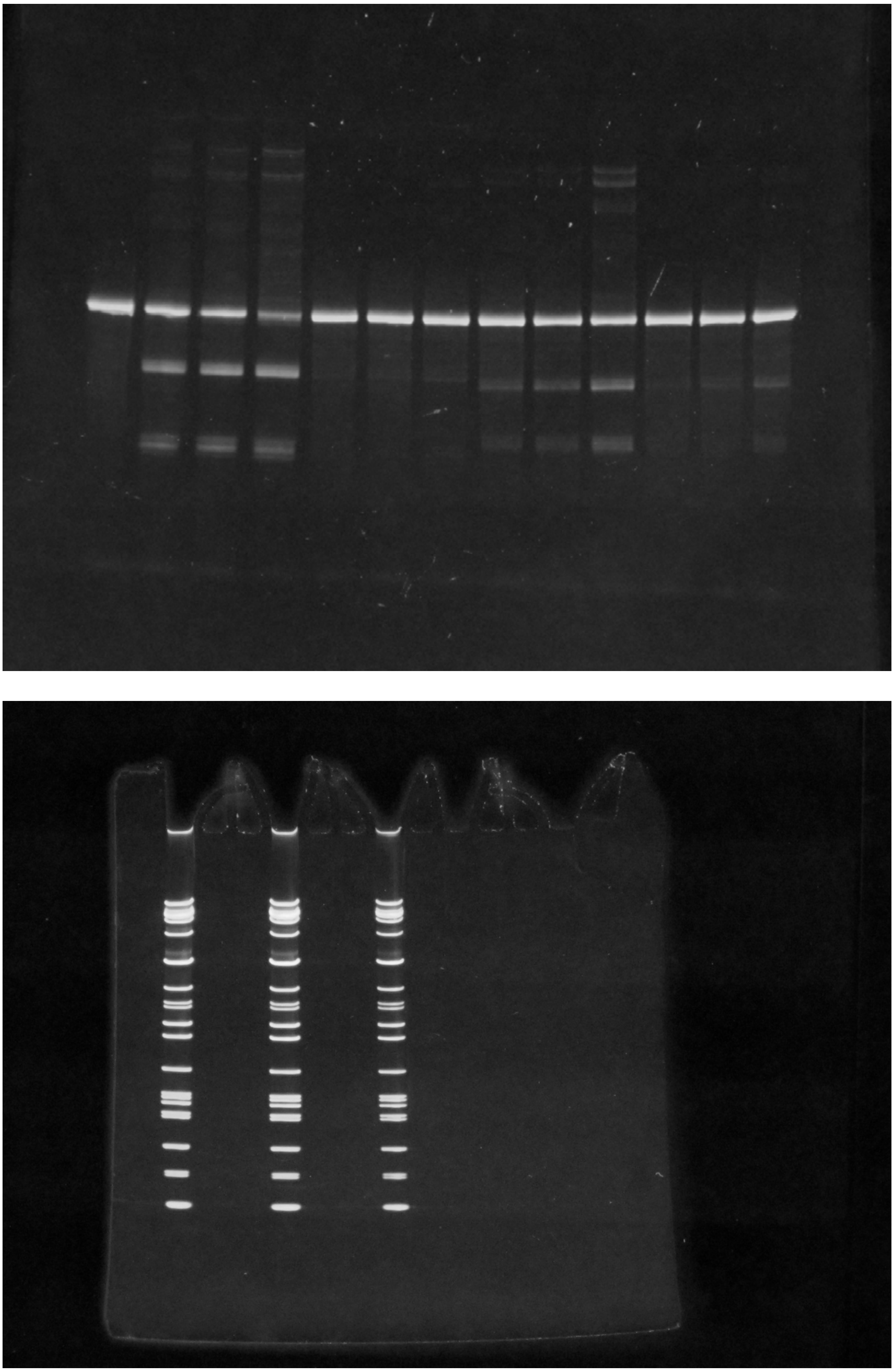

Supplementary Figure S7.

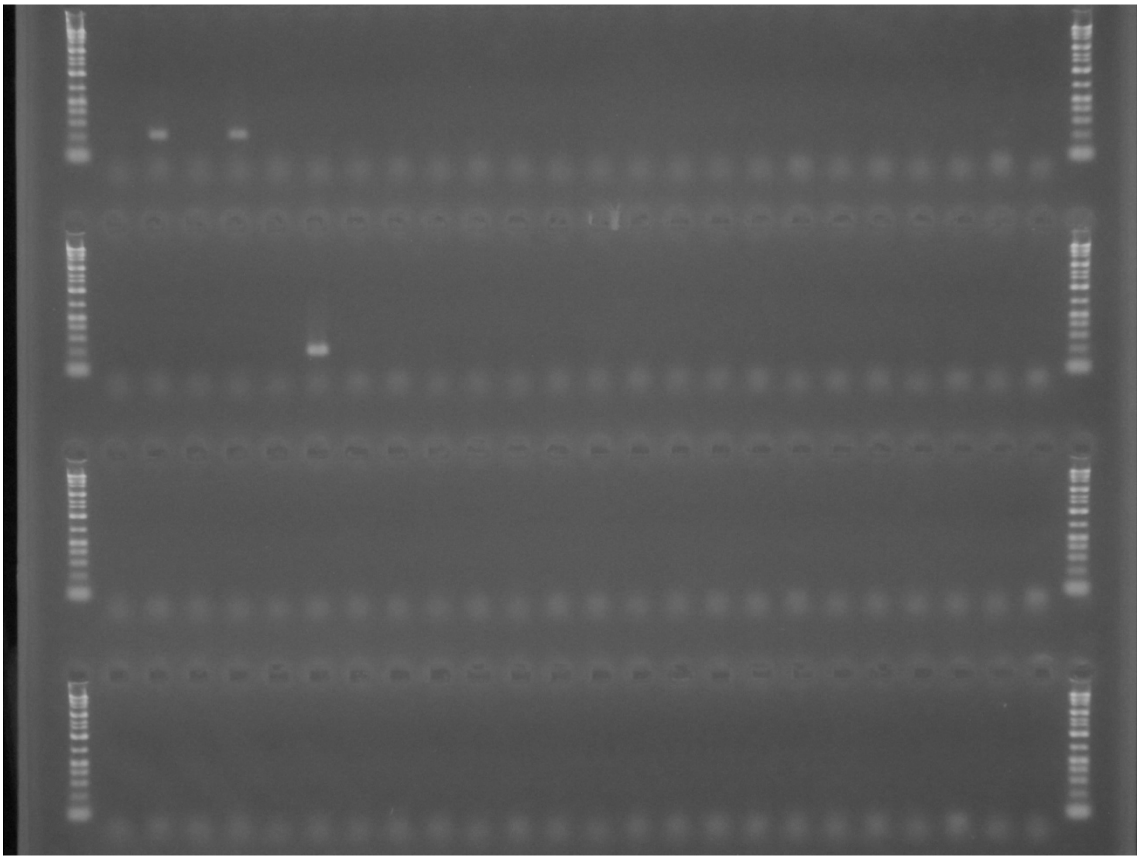

Supplementary Figure S8.

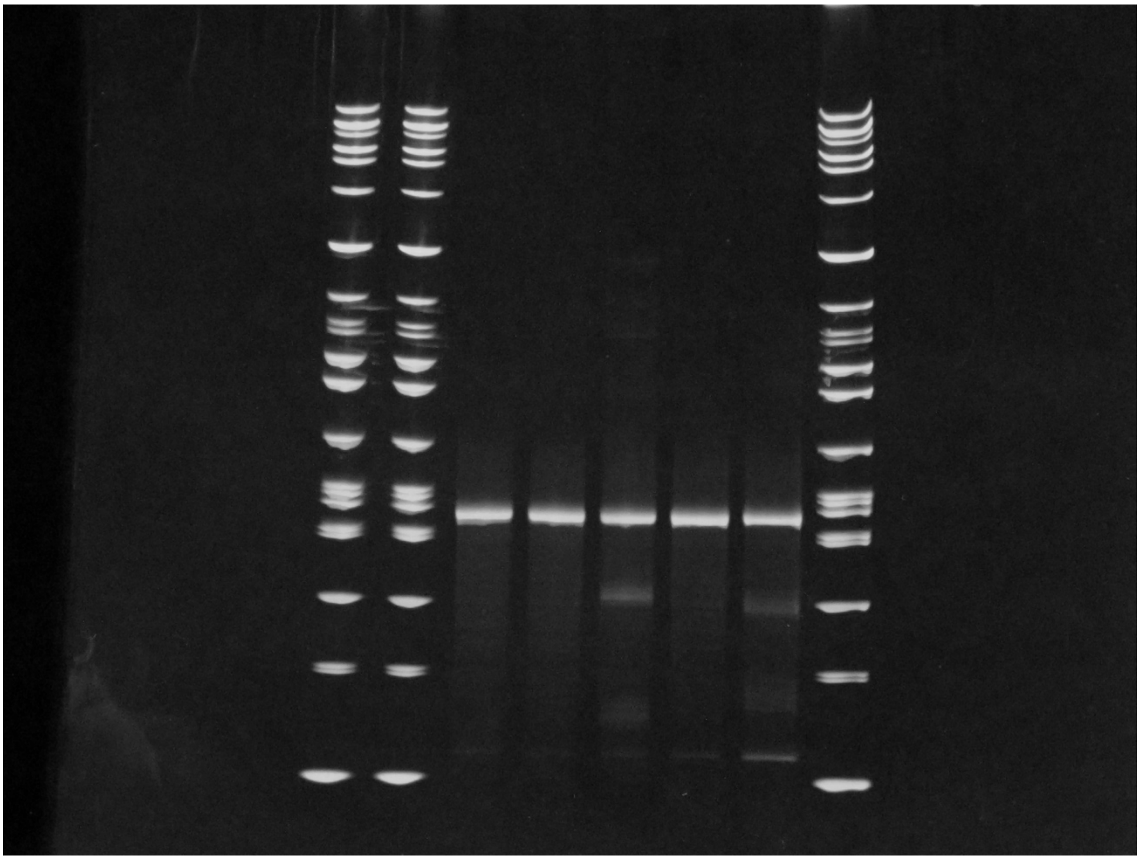

Supplementary Figure S9.

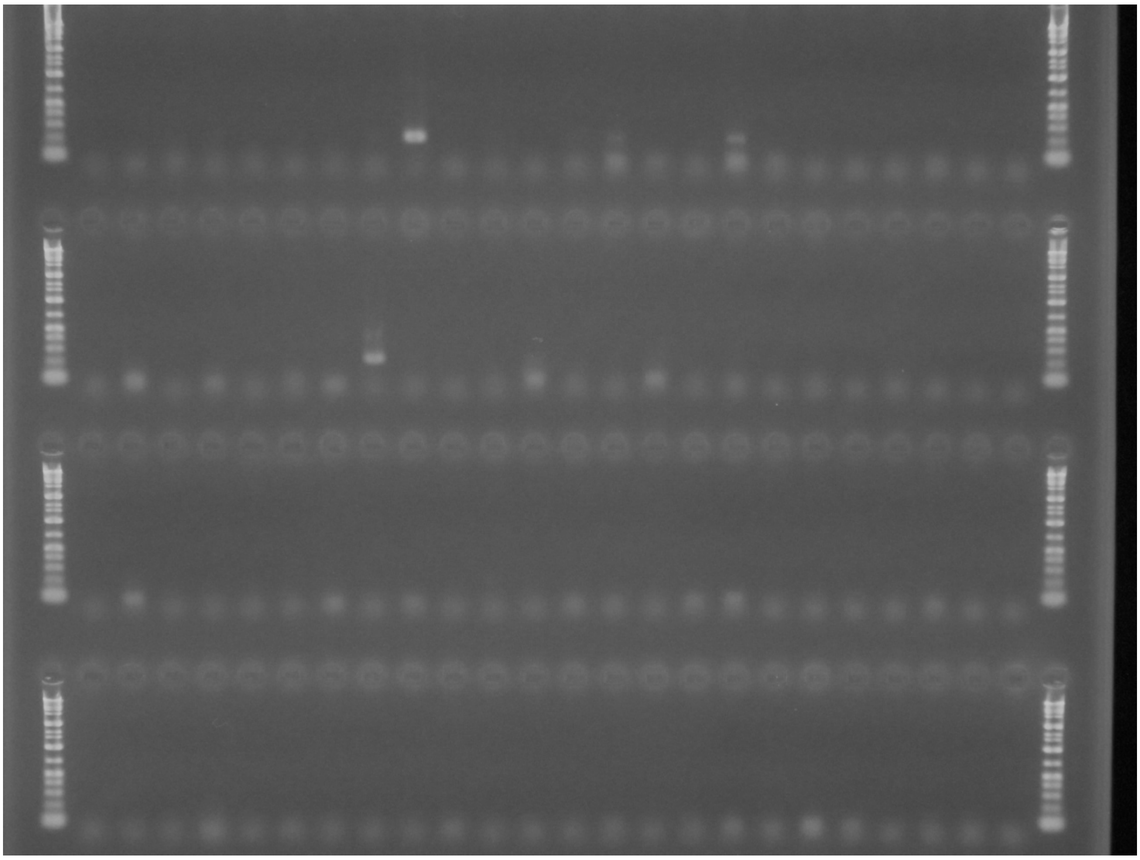

Supplementary Figure S10.

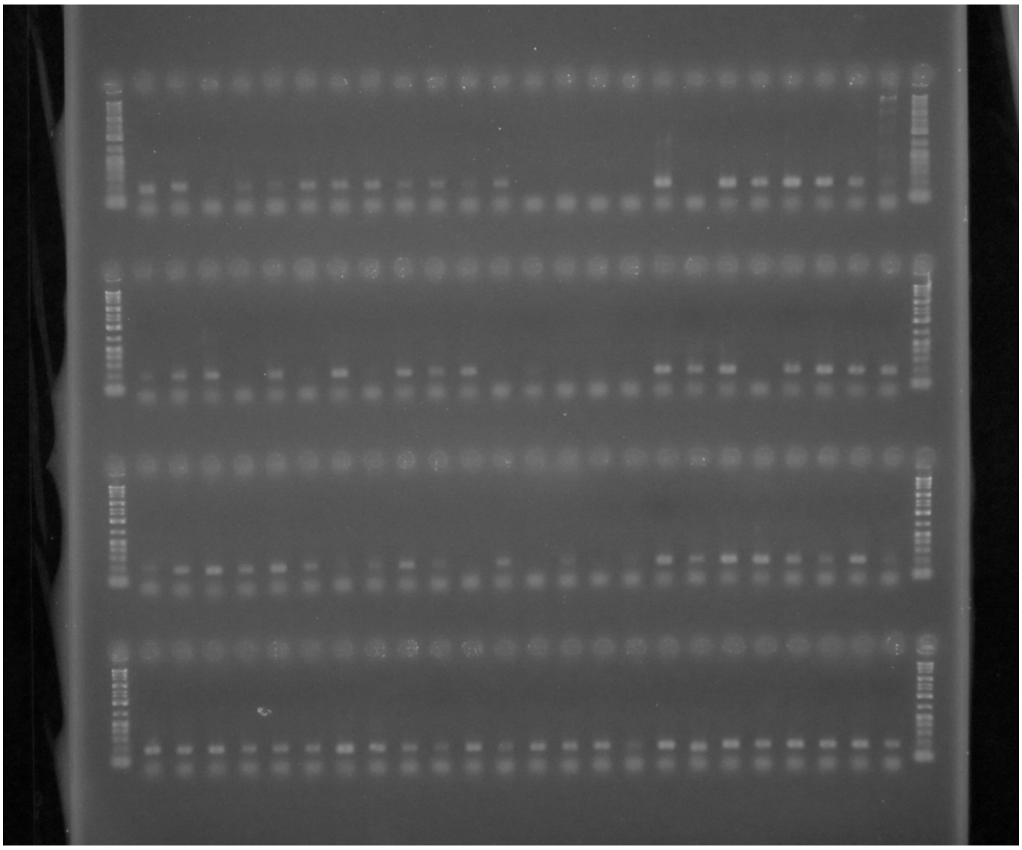

Supplementary Figure S11.

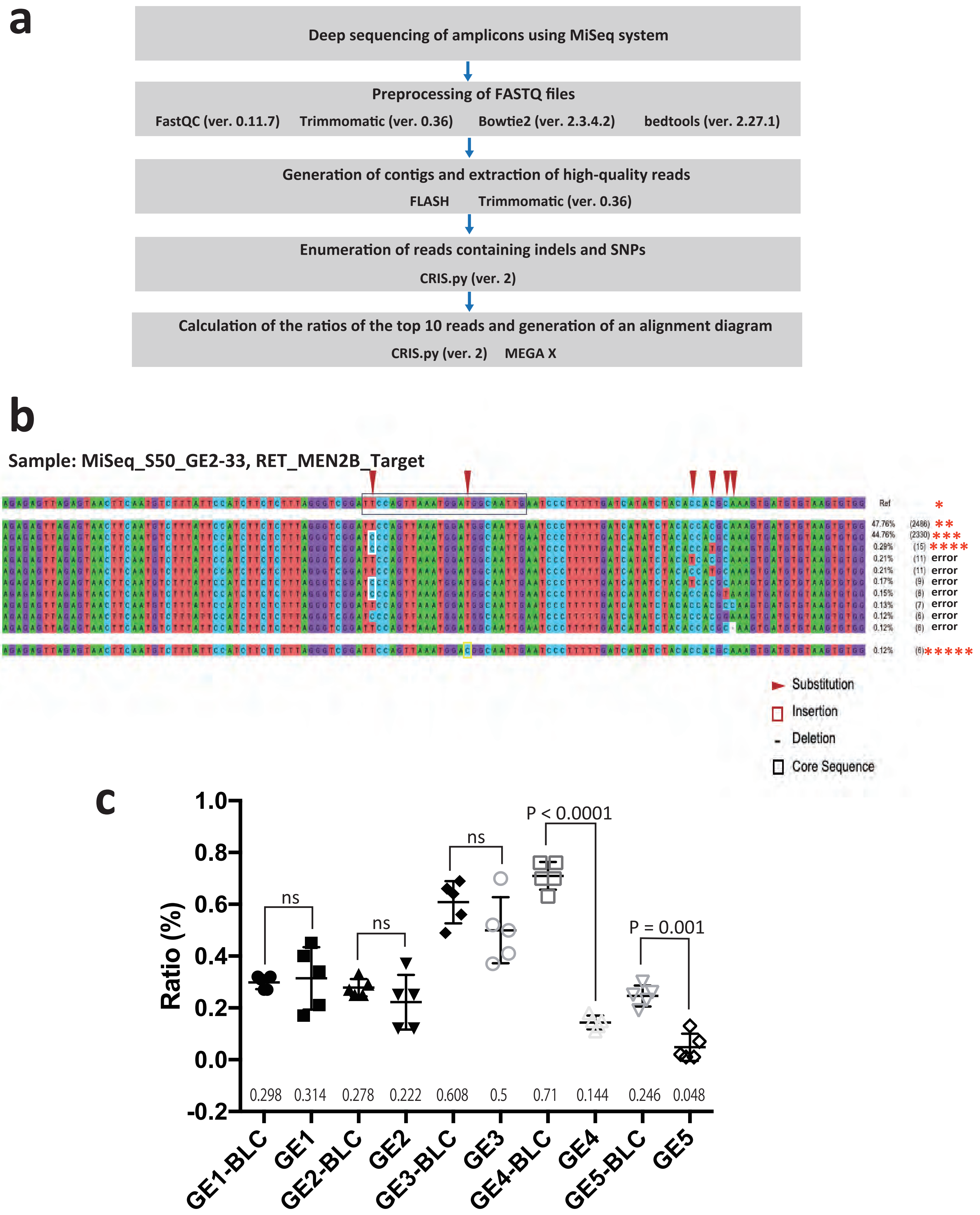

Supplementary Figure X1.

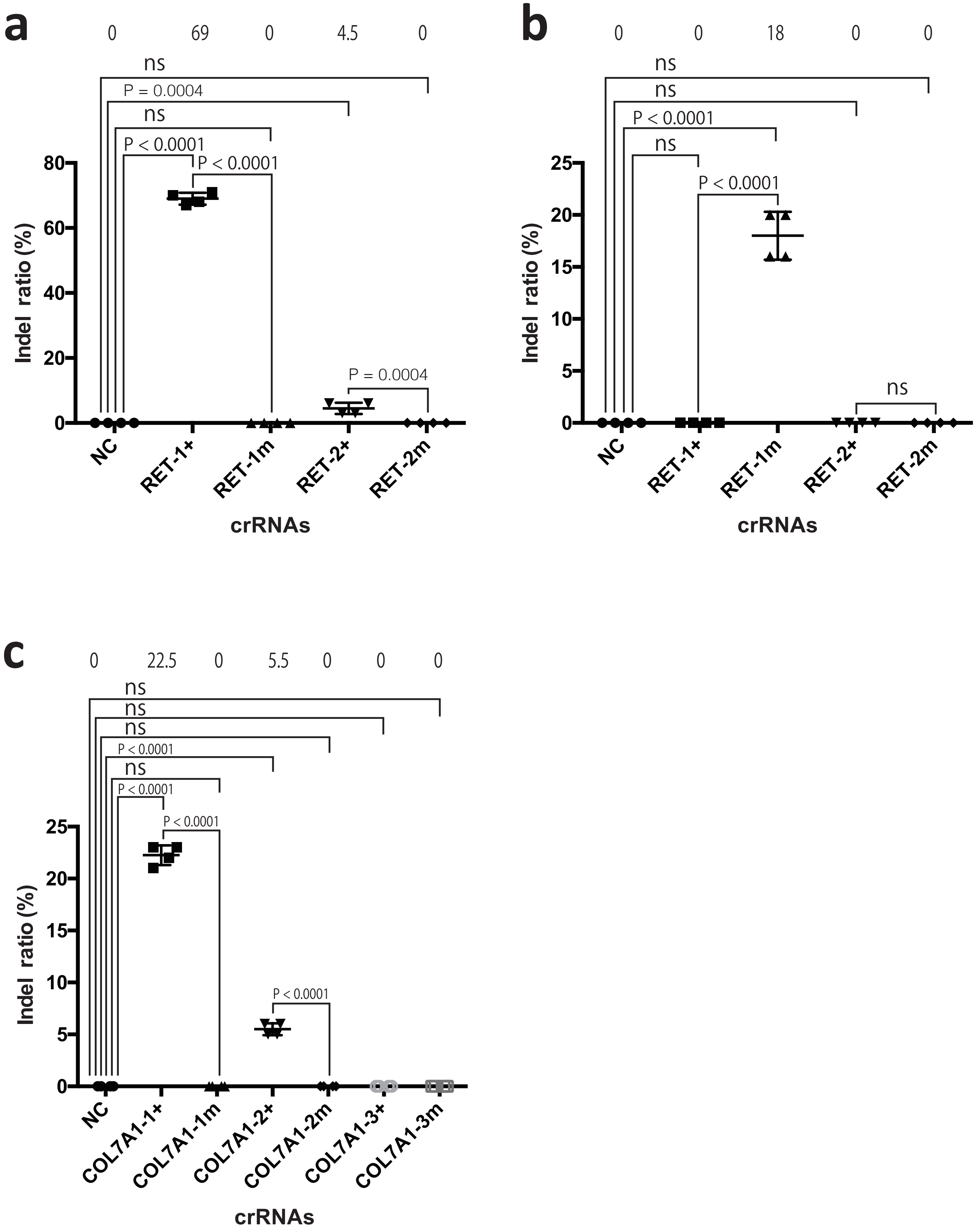

Supplementary Figure X2.

a

```
     5′AGGGTCGGATTCCAGTTAAATGGATGGCAATTGAATCCCTTTTTGATCATATCTACACCA3′(Ref)
GE1re
 1   AGGGTCGGATTCCAGTTAAATGGACGGCAATTGAATCCCTTTTTGATCATATCTACACCA (Mut)   (52.7%)
 2   AGGGTCGGATTCCAGTTAAATGGATGGCAATTGAATCCCTTTTTGATCATATCTACACCA (WT)    (18.3%)
 3   AGGGTCGGATTCCAGTTAAATGGATGG------AATCCCTTTTTGATCATATCTACACCA (-6)    (4.74%)
 4   AGGGTCGGATCCCAGTTAAATGGACGGCAATTGAATCCCTTTTTGATCATATCTACACCA (Mod)   (2.54%)
 5   AGGGTCGGATTCCAGTTAAATGGA-----------TCCCTTTTTGATCATATCTACACCA (-11)   (1.70%)
 6   AGGGTCGGATTCCAGTTAAATGGATGGC----GAATCCCTTTTTGATCATATCTACACCA (-4)    (1.69%)
 7   AGGGTCGGATTCCAGTTAAATGGATGGC------ATCCCTTTTTGATCATATCTACACCA (-4)    (1.54%)
 8   AGGGTCGGATTCCAGTTAAATGGATG-------AATCCCTTTTTGATCATATCTACACCA (-6)    (1.20%)
 9   AGGGTCGGATTCCAGTTAAATGGATGG--------TCCCTTTTTGATCATATCTACACCA (-7)    (0.95%)
10   AGGGTCGGATTCCAGTTAAATGGATGGCA----AATCCCTTTTTGATCATATCTACACCA (-4)    (0.90%)
```

b

```
     5′AGGGTCGGATTCCAGTTAAATGGACGGCAATTGAATCCCTTTTTGATCATATCTACACCA3′(Ref*)
GE2re
 1   AGGGTCGGATTCCAGTTAAATGGATGGCAATTGAATCCCTTTTTGATCATATCTACACCA (WT)    (52.4%)
 2   AGGGTCGGATTCCAGTTAAATGGACGGCAATTGAATCCCTTTTTGATCATATCTACACCA (Mut)   (18.6%)
 3   AGGGTCGGATCCCAGTTAAATGGATGGCAATTGAATCCCTTTTTGATCATATCTACACCA (Rep)   (2.45%)
 4   AGGGTCGGATTCCAGTTAAATGGACGG------AATCCCTTTTTGATCATATCTACACCA (-6)    (2.15%)
 5   AGGGTCGGATTCCAGTTAAATGGACGGCA------TCCCTTTTTGATCATATCTACACCA (-6)    (2.00%)
 6   AGGGTCGGATTCCAGTTAAATGGACGGCAA-----TCCCTTTTTGATCATATCTACACCA (-5)    (1.02%)
 7   AGGGTCGGATTCCAGTTAAATGGACGGC---------CCTTTTTGATCATATCTACACCA (-9)    (0.76%)
 8   AGGGTCGGATTCCAGTTAAATGGACGGCAA------CCCTTTTTGATCATATCTACACCA (-6)    (0.67%)
 9   AGGGTCGGATTCCAGTTAAATGGACGG---------------------ATATCTACACCA (-21)   (0.50%)
10   AGGGTCGGATTCCAGTTAAATGGACGG--------TCCCTTTTTGATCATATCTACACCA (-8)    (0.47%)
```

c

```
     5′AGGGTCGGATTCCAGTTAAATGGACGGCAATTGAATCCCTTTTTGATCATATCTACACCA3′(Ref*)
GE3re
 1   AGGGTCGGATTCCAGTTAAATGGATGGCAATTGAATCCCTTTTTGATCATATCTACACCA (WT)    (54.3%)
 2   AGGGTCGGATTCCAGTTAAATGGACGGCAATTGAATCCCTTTTTGATCATATCTACACCA (Mut)   (22.0%)
 3   AGGGTCGGATTCCAGTTAAATGGATGGCAATCGAATCCCTTTTTGATCATATCTACACCA (Rep)   (5.44%)
 4   AGGGTCGGATTCCAGTTAAATGGACGGAA------TCCCTTTTTGATCATATCTACACCA (-6)    (1.67%)
 5   AGGGTCGGATTCCAGTTAAATGGACGGCAAT-----CCCTTTTTGATCATATCTACACCA (-5)    (0.82%)
 6   AGGGTCGGATTCCAGTTAAATGGACGGC----------CTTTTTGATCATATCTACACCA (-10)   (0.46%)
 7   AGGGTCGGATTCCAGTTAAATGGATGGCAATTGAATCCCTTTTTGATCATATCTACATCA (error)(0.39%)
 8   AGGGTCGGATTCCAGTTAAATGGATGGCAATTGAATCCCTTTTTGATCATATCTACACCA (error)(0.39%)
 9   AGGGTCGGATTCCAGTTAAATGGAC----------TCCCTTTTTGATCATATCTACACCA (-10)   (0.33%)
10   AGGGTCGGATTCCAGTTAAATGGACGGCA------TCCCTTTTTGATCATATCTACACCA (-6)    (0.29%)
```

d

```
     5′AGGGTCGGATTCCAGTTAAATGGACGGCAATTGAATCCCTTTTTGATCATATCTACACCA3′(Ref*)
GE4re
 1   AGGGTCGGATTCCAGTTAAATGGATGGCAATTGAATCCCTTTTTGATCATATCTACACCA (WT)    (63.1%)
 2   AGGGTCGGATTCCAGTTAAATGGACGGCAATTGAATCCCTTTTTGATCATATCTACACCA (Mut)   (18.6%)
 3   AGGGTCGGATTCCAGTTAAATGGACGG------AATCCCTTTTTGATCATATCTACACCA (-6)    (2.36%)
 4   AGGGTCGGATTCCAGTTAAATGGA----------------------TCATATCTACACCA (-22)   (0.79%)
 5   AGGGTCGGATTCCAGTTAAATGGACG-CAATTGAATCCCTTTTTGATCATATCTACACCA (-1)    (0.71%)
 6   AGGGTCGGATTCCAGTTAAATGGACGGCA------TCCCTTTTTGATCATATCTACACCA (-6)    (0.59%)
 7   AGGGTCGGATTCCAGTTAAATGGACGGC---------CCTTTTTGATCATATCTACACCA (-9)    (0.57%)
 8   AGGGTCGGATTCCAGTTAAATGGACGGCAAT-----CCCTTTTTGATCATATCTACACCA (-5)    (0.49%)
 9   AGGGTCGGATTCCAGTTAAATGGACGGCAA----ATCCCTTTTTGATCATATCTACACCA (-4)    (0.39%)
10   AGGGTCGGATTCCAGTTAAATGGACGGCA-----------TTTTGATCATATCTACACCA (-11)   (0.39%)
```

e

```
     5′CTCTGCCTTCAGGGTGTGCCAGGCATCAAATGAGACCGGGGAGAGCCTGGACCGAGGGGT3′(Ref* )
     3′GAGACGGAAGTCCCACACGGTCCGTAGTTTACTCTGGCCCCTCTCGGACCTGGCTCCCCA5′(Comp*)
GE5re
 1   CTCTGCCTTCAGGGTGTGCCAGGCATCAAAGGAGACCGGGGAGAGCCTGGACCGAGGGGT (WT)    (51.4%)
 2   CTCTGCCTTCAGGGTGTGCCAGGCATCAAATGAGACCGGGGAGAGCCTGGACCGAGGGGT (Mut)   (30.0%)
 3   CTCTGCCTTCAGGGTGTGCCAGGCAT-----GAGACCGGGGAGAGCCTGGACCGAGGGGT (-5)    (5.08%)
 4   CTCTGCCTTCAGGGTGTGCCAGGCATCA-----GACCGGGGAGAGCCTGGACCGAGGGGT (-5)    (2.13%)
 5   CTCTGCCTTCAGGGTGTGCCAGGCATCA--TGAGACCGGGGAGAGCCTGGACCGAGGGGT (-2)    (0.57%)
 6   CTCTGCCTTCAGGGTGTGCCAGGCATC----GAGACCGGGGAGAGCCTGGACCGAGGGGT (-4)    (0.41%)
 7   CTCTGCCTTCAGGGTGTGCCAGG----------------GGAGAGCCTGGACCGAGGGGT (-16)   (0.41%)
 8   CTCTGCCTTCAGGGTGTGCCAGGCATCAA----GACCGGGGAGAGCCTGGACCGAGGGGT (-4)    (0.41%)
 9   CTCTGCCTTCAGGGTGTGCCAGGCATCAAA-----CCGGGGAGAGCCTGGACCGAGGGGT (-5)    (0.41%)
10   CTCTGCCTTCAGGGTGTGCCAGGCATCAA-TGAGACCGGGGAGAGCCTGGACCGAGGGGT (-1,+2)(0.33%)
                                   tt
```

Supplementary Figure X3.

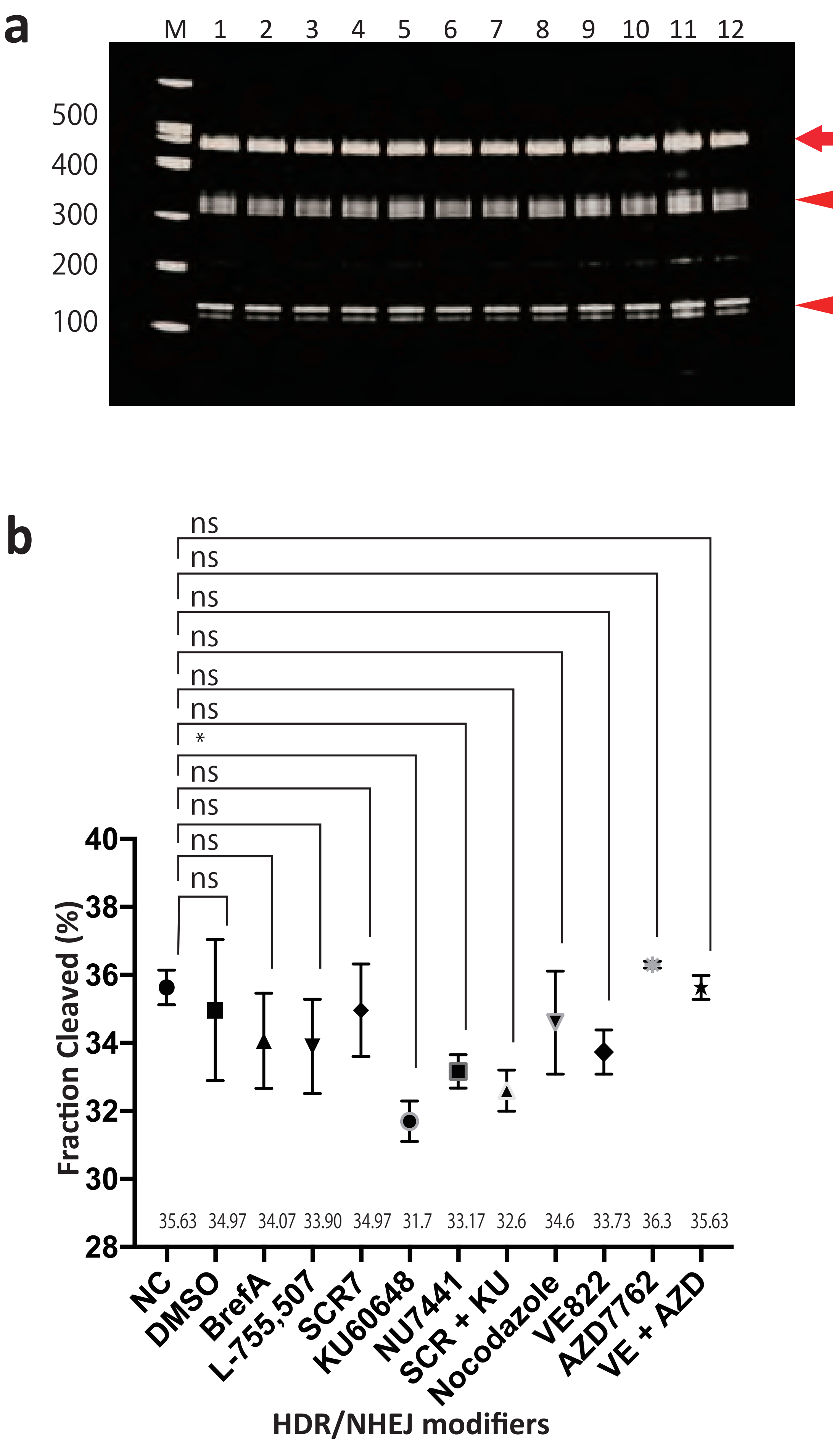

Supplementary Figure X4.

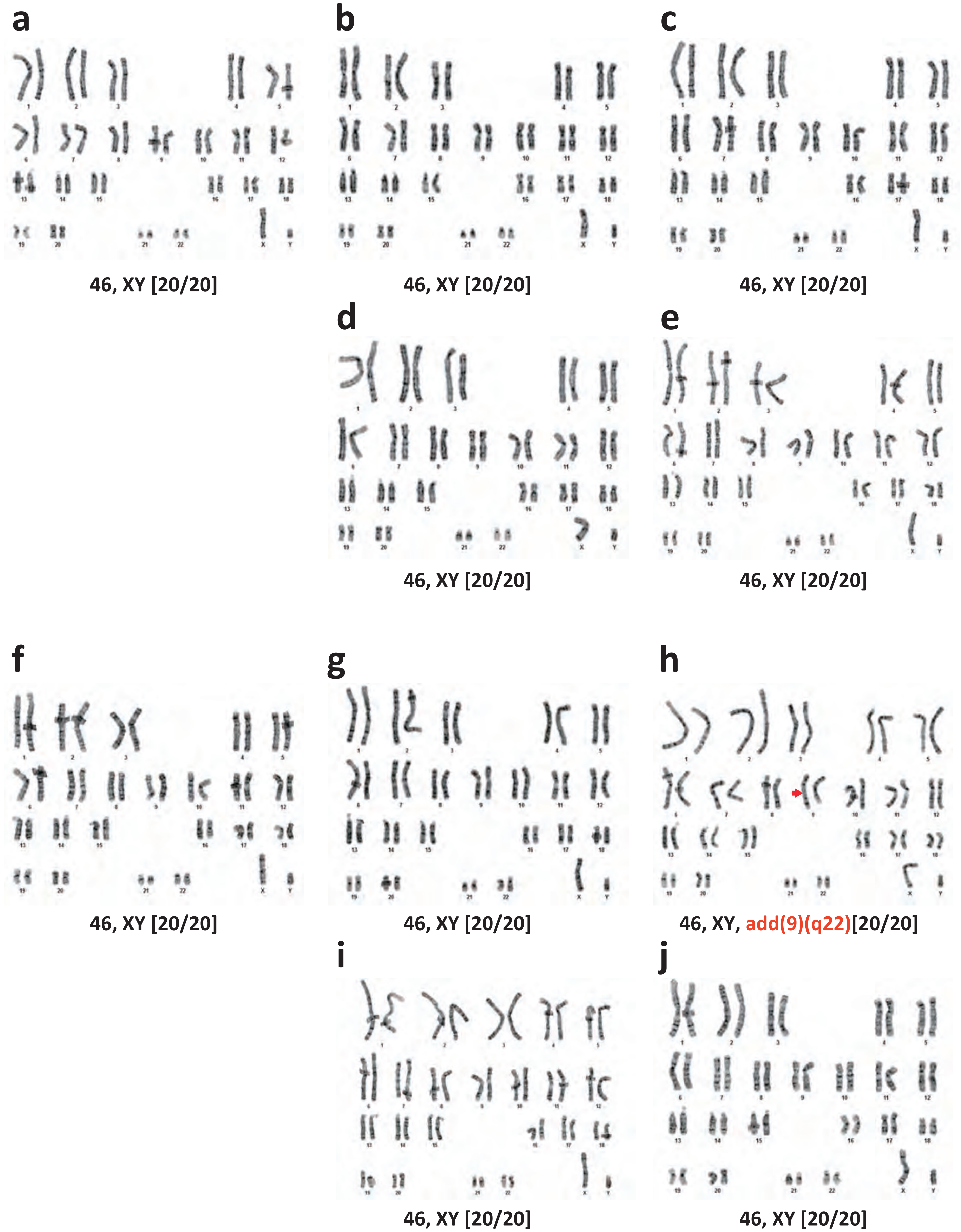

Supplementary Figure X5.

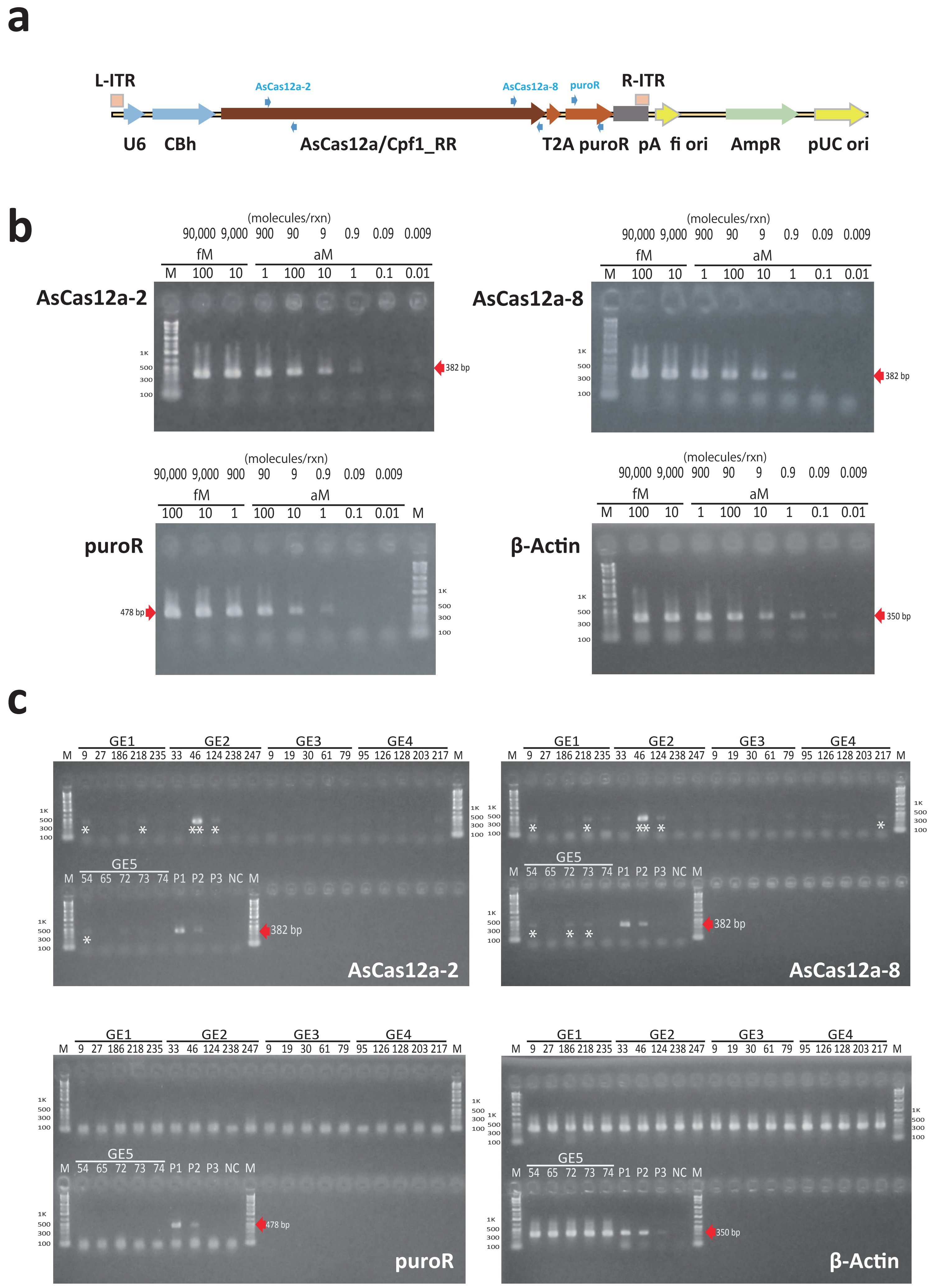

Supplementary Figure X6.

a **FB4-14**

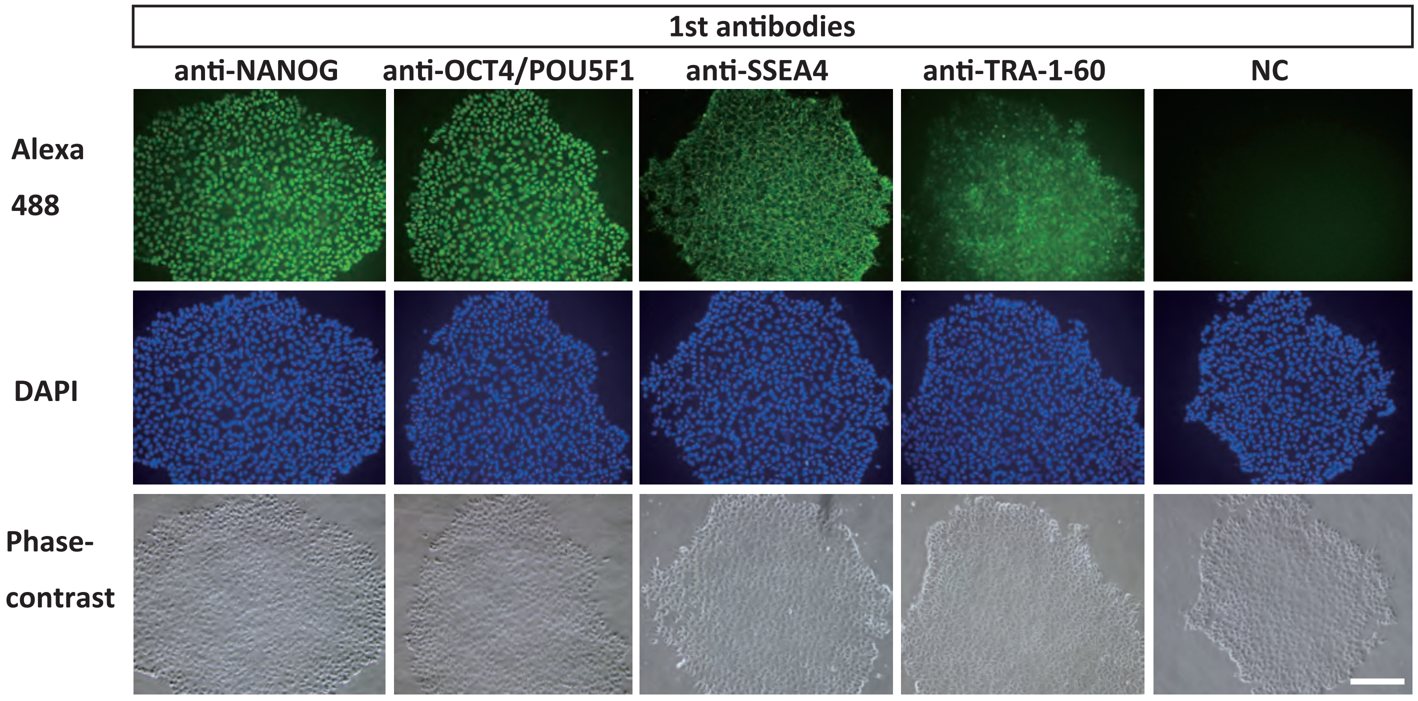

b **B117-3**

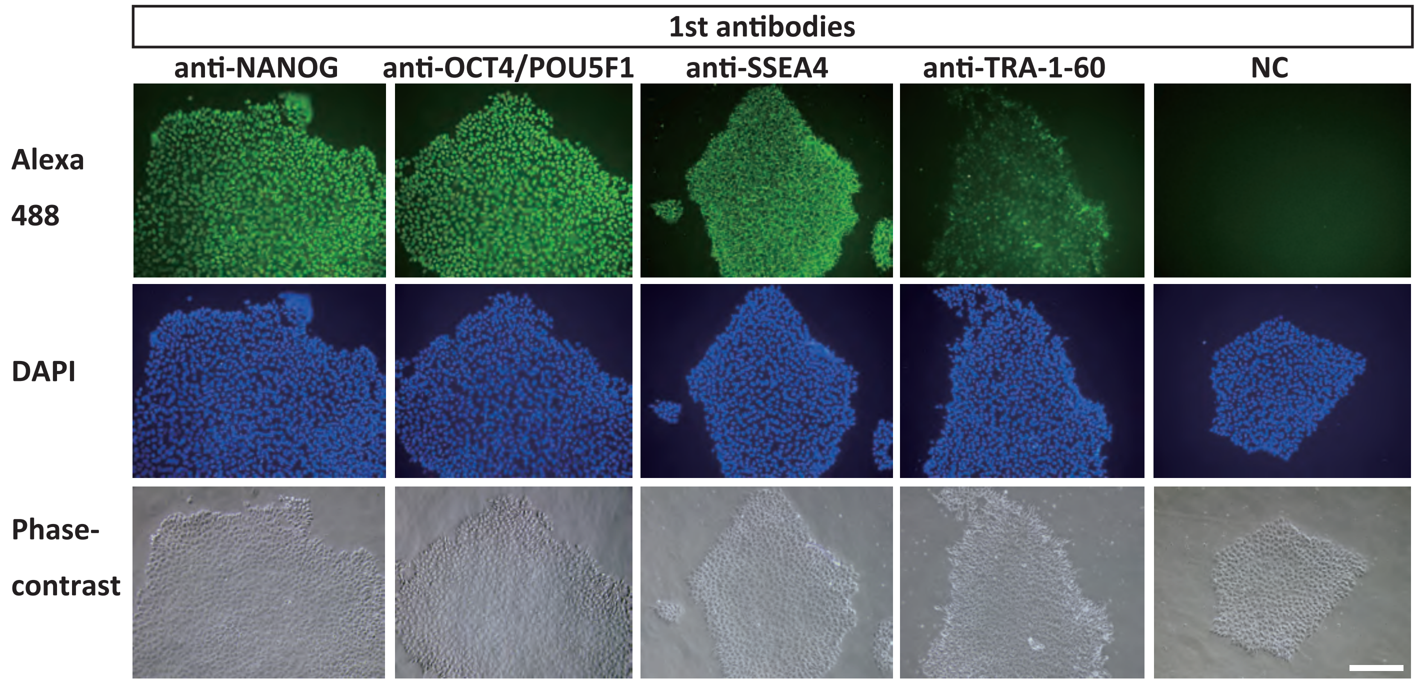

Supplementary Figure X7.

a GE2

	Sequence	Classification	N	ID No.
1	WT: 5′ AGGGTCGGATTCCAGTTAAATGGATGGCAATTGAATCCCTTTTTGATCATATCTACACCACGCAAAG 3′<br>Target: 5′ AGGGTCGGATTCCAGTTAAATGGACGGCAATTGAATCCCTTTTTGATCATATCTACACCACGCAAAG 3′	original	x0	
2	AGGGTCGGATTCCAGTTAAATGGATGGCAATTGAATCCCTTTTTGATCATATCTACACCACGCAAAG<br>AGGGTCGGATCCCAGTTAAATGGATGGCAATTGAATCCCTTTTTGATCATATCTACACCACGCAAAG	repaired	x11	#33, 46, 70, 124, 238, 245, 247, 292, 297, 309, 329
3	AGGGTCGGATTCCAGTTAAATGGATGGCAATTGAATCCCTTTTTGATCATATCTACACCACGCAAAG<br>AGGGTCGGATCCCAGTTAAATGGATGGCA------TCCCTTTTTGATCATATCTACACCACGCAAAG	repaired, -6	x1	#22
4	AGGGTCGGATTCCAGTTAAATGGATGGCAATTGAATCCCTTTTTGATCATATCTACACCACGCAAAG<br>AGGGTCGGATCCCAGTTAAATGGATGGCA-TTGAATCCCTTTTTGATCATATCTACACCACGCAAAG	repaired, -1	x1	#193
5	AGGGTCGGATTCCAGTTAAATGGATGGCAATTGAATCCCTTTTTGATCATATCTACACCACGCAAAG<br>AGGGTCGGATTCCAGTTAAATGGACGGCAAT-----CCCTTTTTGATCATATCTACACCACGCAAAG	-5	x3	#101, 111, 290
6	AGGGTCGGATTCCAGTTAAATGGATGGCAATTGAATCCCTTTTTGATCATATCTACACCACGCAAAG<br>AGGGTCGGATTCCAGTTAAATGGACGGCAA--------CTTTTTGATCATATCTACACCACGCAAAG	-8	x1	#161

Note. Forward primer for SNMD-PCR: #1856 CCATCTTCTCTTTAGGGTCGGATC

b GE4

	Sequence	Classification	N	ID No.
1	WT: 5′ AGGGTCGGATTCCAGTTAAATGGATGGCAATTGAATCCCTTTTTGATCATATCTACACCACGCAAAG 3′<br>Target: 5′ AGGGTCGGATTCCAGTTAAATGGACGGCAATTGAATCCCTTTTTGATCATATCTACACCACGCAAAG 3′	original	x7	#98, 103,111, 112, 124 220, 224
2	AGGGTCGGATTCCAGTTAAATGGATGGCAATTGAATCCCTTTTTGATCATATCTACACCACGCAAAG<br>AGGGTCGGATTCCAGTTAAATGGATGGCAATTGAATCCCTTTTTGATCATATCTACACCACGCAAAG	repaired	x5	#95, 126, 128, 203, 217
3	AGGGTCGGATTCCAGTTAAATGGATGG-----------CTTTTTGATCATATCTACACCACGCAAAG<br>AGGGTCGGATTCCAGTTAAATGGAt-----------CCCTTTTTGATCATATCTACACCACGCAAAG	-11/-12, +1	x2	#8, 11
4	AGGGTCGGATTCCAGTTAAATGGATGGCAATTGAATCCCTTTTTGATCATATCTACACCACGCAAAG<br>AGGGTCGGATTCCAGTTAAATGGACGGCAAT-------------------------ACCACGCAAAG	-25	x1	#104
5	AGGGTCGGATTCCAGTTAAATGGATGGCAATTGAATCCCTTTTTGATCATATCTACACCACGCAAAG<br>AGGGTCGGATTCCAGTTAAATGGACGGC----GAATCCCTTTTTGATCATATCTACACCACGCAAAG	-4	x1	#127
6	AGGGTCGGATTCCAGTTAAATGGATGGCAATTGAATCCCTTTTTGATCATATCTACACCACGCAAAG<br>AGGGTCGGATTCCAGTTAAATGGACG------------------------------------CAAAG	-36	x1	#136
7	AGGGTCGGATTCCAGTTAAATGGATGGCAATTGAATCCCTTTTTGATCATATCTACACCACGCAAAG<br>AGGGTCGGATTCCAGTTAAATGGACGGCA------TCCCTTTTTGATCATATCTACACCACGCAAAG	-6	x1	#144
8	AGGGTCGGATTCCAGTTAAATGGATGGCAATTGAATCCCTTTTTGATCATATCTACACCACGCAAAG<br>AGGGTCGGATTCCAGTTAAATGGACGGCAAT-----CCCTTTTTGATCATATCTACACCACGCAAAG	-5	x1	#165
9	AGGGTCGGATTCCAGTTAAATGGATGGCAATTGAATCCCTTTTTGATCATATCTACACCACGCAAAG<br>AGGGTCGGATTCCAGTTAAATGGAC-----------CCCTTTTTGATCATATCTACACCACGCAAAG	-11	x1	#79
10	AGGGTCGGATTCCAGTTAAATGGATGGCAATTGAATCCCTTTTTGATCATATCTACACCACGCAAAG<br>AGGGTCGGATTCCAGTTAAATGGA----------ATCCCTTTTTGATCATATCTACACCACGCAAAG	-10	x1	#216
11	AGGGTCGGATTCCAGTTAAATGGATGGCAATTGAATCCCTTTTTGATCATATCTACACCACGCAAAG<br>AGGGTCGGATTCCA---CACCACGCAAAG	-41	x1	#226
12	AGGGTCGGATTCCAGTTAAATGGATGGCAATTGAATCCCTTTTTGATCATATCTACACCACGCAAAG<br>AGGGTCGGATTCCAGTT------------------------TTTGATCATATCTACACCACGCAAAG	-24	x1	#230
13	AGGGTCGGATTCCAGTTAAATGGATGGCAATTGAATCCCTTTTTGATCATATCTACACCACGCAAAG<br>AGGGTCGGATTCCA---AG	-51	x1	#231

Note. Forward primer for SNMD-PCR: #1749 GGGTCGGATTCCAGTTAAATGGAC

Supplementary Figure X8.

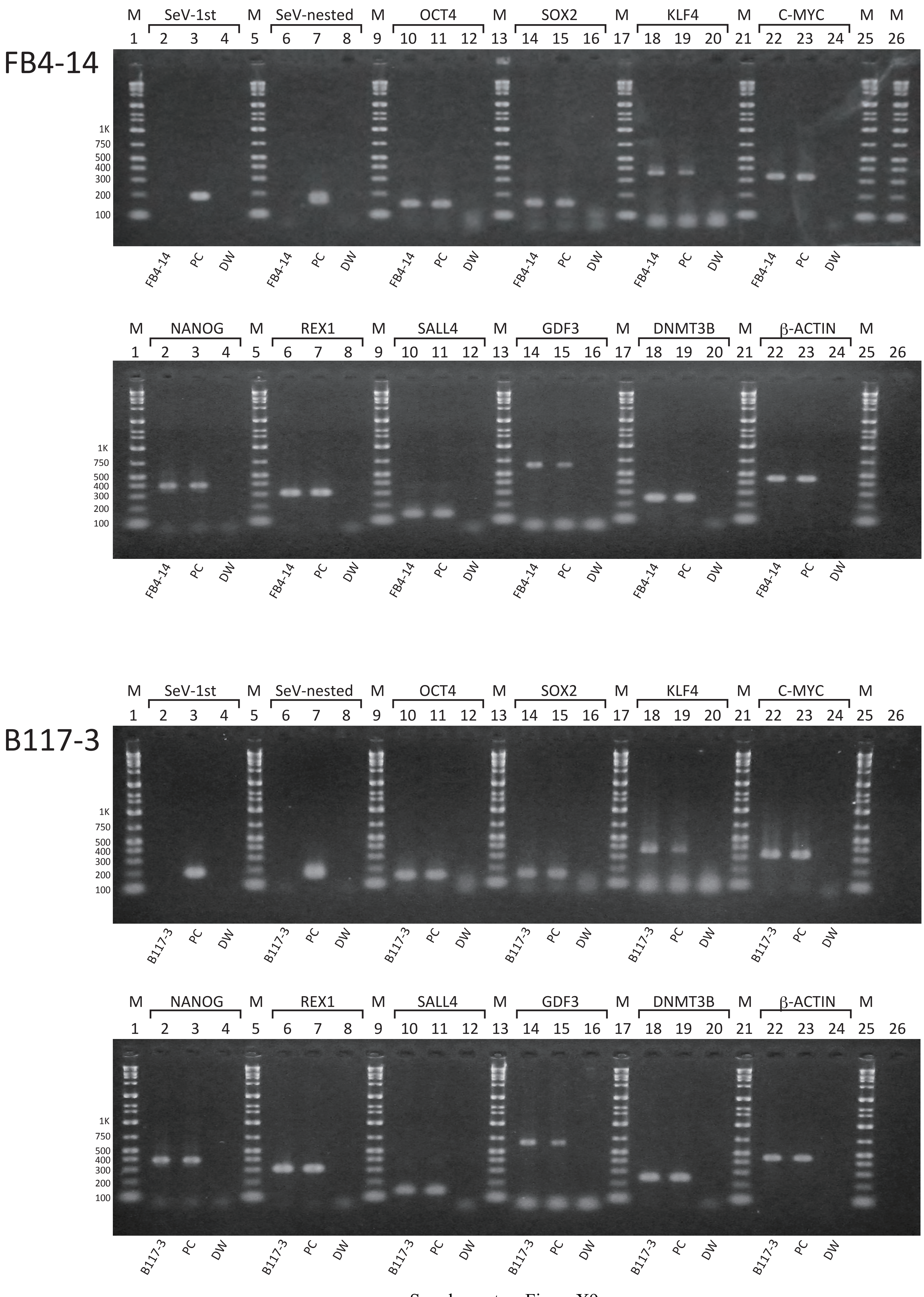

Supplementary Figure X9.
